# ASCL1-regulated DARPP-32 and t-DARPP stimulate small cell lung cancer growth and neuroendocrine tumour cell proliferation

**DOI:** 10.1038/s41416-020-0923-6

**Published:** 2020-06-05

**Authors:** Sk. Kayum Alam, Li Wang, Yanan Ren, Christina E. Hernandez, Farhad Kosari, Anja C. Roden, Rendong Yang, Luke H. Hoeppner

**Affiliations:** 1 The Hormel Institute, University of Minnesota, Austin, MN USA; 2 Department of Molecular Medicine, Mayo Clinic, Rochester, MN USA; 3 Department of Laboratory Medicine and Pathology, Mayo Clinic, Rochester, MN USA; 4 Masonic Cancer Center, University of Minnesota, Minneapolis, MN USA

**Keywords:** Small-cell lung cancer, Mechanisms of disease

## Abstract

**Background:**

Small cell lung cancer (SCLC) is the most aggressive form of lung cancer, and new molecular insights are necessary for prognostic and therapeutic advances.

**Methods:**

Dopamine and cAMP-regulated phosphoprotein, Mr 32000 (DARPP-32) and its N-terminally truncated splice variant, t-DARPP, were stably overexpressed or ablated in human DMS-53 and H1048 SCLC cells. Functional assays and immunoblotting were used to assess how DARPP-32 isoforms regulate SCLC cell growth, proliferation, and apoptosis. DARPP-32-modulated SCLC cells were orthotopically injected into the lungs of SCID mice to evaluate how DARPP-32 and t-DARPP regulate neuroendocrine tumour growth. Immunostaining for DARPP-32 proteins was performed in SCLC patient-derived specimens. Bioinformatics analysis and subsequent transcription assays were used to determine the mechanistic basis of DARPP-32-regulated SCLC growth.

**Results:**

We demonstrate in mice that DARPP-32 and t-DARPP promote SCLC growth through increased Akt/Erk-mediated proliferation and anti-apoptotic signalling. DARPP-32 isoforms are overexpressed in SCLC patient-derived tumour tissue, but undetectable in physiologically normal lung. Achaete-scute homologue 1 (ASCL1) transcriptionally activates DARPP-32 isoforms in human SCLC cells.

**Conclusions:**

We reveal new regulatory mechanisms of SCLC oncogenesis that suggest DARPP-32 isoforms may represent a negative prognostic indicator for SCLC and serve as a potential target for the development of new therapies.

## Background

Lung cancer is the leading cause of cancer death among both men and women in developed countries worldwide.^1^ Small cell lung cancer (SCLC) unfortunately kills ~250,000 people worldwide annually and comprises 15% of the incidences of lung cancer.^2^ Cigarette smoking is the most common cause of SCLC, and SCLC patients have a smoking history in 95% of cases.^3^ SCLC treatment options have not substantially changed since the introduction of cisplatin and etoposide decades ago.^3^ SCLC is the most aggressive form of lung cancer due to its rapid doubling time and early widespread metastasis.^4^ Most cases of SCLC are tumours of neuroendocrine origin with frequent inactivation of *TP53* and *RB1* as well as disruption of several molecular pathways, including Notch signalling.^2^ SCLC patients typically present with advanced disease, respond to initial systemic chemotherapy, and then treatment refractory progression usually occurs within one year due to acquired drug resistance. Consequently, the median survival time of SCLC patients is only 9 to 20 months and merely 7% of SCLC patients survive beyond five years.^4,5^ The frequent, rapid, and pronounced biological transition from chemotherapy-sensitive to chemotherapy-resistant SCLC underscores the importance of identifying therapeutically targetable molecular drivers of acquired resistance.

Dopamine and cyclic adenosine monophosphate-regulated phosphoprotein, Mr 32000 (DARPP-32) is an effector molecule that plays an important role in dopaminergic neurotransmission. Upstream of DARPP-32, dopamine D2 receptor agonists have been shown to inhibit lung tumour angiogenesis,^6^ and clinical trials of selective dopamine D2 and D3 receptor antagonists have demonstrated anti-cancer efficacy in several cancer types other than lung.^7^ Recent reports suggest aberrant DARPP-32 overexpression promotes oncogenesis in lung,^8^ gastric,^9^ colon,^10^ prostate,^11^ oesophagus^12^ and breast adenocarcinomas^13^ through regulation of proliferation,^14^ survival,^15^ migration,^8^ invasion,^16^ and angiogenesis.^17^ However, the role of DARPP-32 in neuroendocrine tumours remains unexplored. In the early 2000s, El-Rifai et al. discovered that DARPP-32 and its novel transcriptional splice variant are frequently amplified and upregulated in gastric cancer.^9,18^ The N-terminally truncated isoform of DARPP-32, named t-DARPP, uses a unique alternative first exon located within intron 1 of DARPP-32. DARPP-32 and t-DARPP are translated from a gene termed *phosphoprotein phosphatase-1 regulatory subunit 1B (PPP1R1B)* because full-length DARPP-32 inhibits protein phosphatase 1 (PP-1) activity following PKA-mediated phosphorylation at threonine-34 (T34) position. In turn, DARPP-32 inhibits PKA upon phosphorylation of its T75 residue by cyclin-dependent kinase 5 (Cdk5).^19^ Because t-DARPP lacks the first 36 amino acids of DARPP-32, including the T34 phosphorylation residue, t-DARPP is unable to inhibit PP-1.^9^ Overexpression of t-DARPP in breast cancer has been shown to activate oncogenic PI3K/Akt signalling.^20^ The dual function of DARPP-32 as either a kinase or a phosphatase inhibitor enables it to precisely modulate dopaminergic neurotransmission^19,21^ as well as regulate oncogenic signalling when its isoforms are aberrantly overexpressed in tumour cells.

We recently demonstrated that DARPP-32 and t-DARPP promote non-small cell lung cancer (NSCLC) growth in orthotopic mouse models, reduce apoptosis, activate Akt and Erk signalling, and enhance IKKα-mediated lung tumour cell migration.^8^ Immunostaining of 62 human lung adenocarcinoma tissues showed that t-DARPP expression is elevated with increasing tumour staging score, a metric of tumour progression and growth. Bioinformatics analysis revealed upregulation of t-DARPP correlates with advanced tumour stage and poor overall survival of NSCLC patients.^8^ Other groups have reported that t-DARPP promotes cancer cell survival by upregulation of Bcl2 in an Akt-dependent manner and causes drug resistance by activation of the Akt signalling pathway in breast cancer cells.^15,22^ Studies have demonstrated that activation of Akt signalling by DARPP-32 and t-DARPP in breast and oesophageal adenocarcinoma causes resistance to Herceptin (trastuzumab),^20,22–24^ a monoclonal antibody against HER2 commonly used in combination with chemotherapy to treat HER2-positive cancer. In breast cancer cells, DARPP-32 isoforms have been shown to promote resistance to lapatinib, a small molecule dual inhibitor of HER2/EGFR,^13^ as well as EGFR inhibitors, erlotinib and gefitinib.^25^ Most recently, it has been reported that activation of insulin-like growth factor-1 receptor (IGF1R) signalling in breast cancer cells initiates trastuzumab resistance by t-DARPP.^26^ DARPP-32-mediated activation of IGF1R was also found to promote STAT3 signalling that contributes to gastric tumorigenesis.^27^ Another study demonstrates that DARPP-32 interacts with ERBB3, activates Akt signalling, and exhibits resistance to gefitinib in gastric cancer.^28^

Given the multifaceted role of DARPP-32 and t-DARPP proteins in the oncogenesis of numerous cancer types,^29^ including NSCLC,^8^ we sought to determine whether DARPP-32 isoforms promote SCLC growth. Here, we demonstrate for the first time that DARPP-32 isoforms are regulated by Notch signalling to drive SCLC oncogenesis. Specifically, we show Notch target, achaete-scute homologue 1 (ASCL1), transcriptionally activates the DARPP-32 promoter. The pathogenesis of SCLC commonly involves inactivating Notch mutations, which lead to downstream ASCL1 activation.^30^ Comprehensive genomic profiling of 110 SCLC patients revealed inactivating mutations in Notch family genes in 25% of human SCLC.^31^ Correspondingly, activation of Notch signalling in a SCLC mouse model reduced tumour growth and extended survival of the mutant mice.^31^ Concurrent Notch inactivation and ASCL1 overexpression in SCLC^32^ activates pulmonary neuroendocrine differentiation and stimulates tumour progression.^33,34^ Our results support a model in which Notch inactivation leads to ASCL1-mediated activation of DARPP-32 isoforms, which we demonstrate promote tumour growth in an orthotopic mouse model of human SCLC by stimulating proliferation, Akt/Erk-mediated survival and anti-apoptotic signalling.

## Methods

### Cell culture

Human SCLC cell lines, DMS-53 and H1048, were purchased from The European Collection of Authenticated Cell Cultures (ECACC, Salisbury, UK) via Sigma-Aldrich (St. Louis, MO) and American Type Culture Collection (ATCC, Manassas, VA), respectively, and maintained in RPMI-1640 medium (Corning, Manassas, VA) supplemented with 10% foetal bovine serum (FBS; Millipore, Burlington, MA) and antibiotics. Human 293T cells obtained from ATCC were cultured in Dulbecco’s modified Eagle’s medium (DMEM; Corning). All cells were grown in a humidified chamber at 37 °C supplied with 5% CO_2_ in media supplemented with 10% FBS, 1% Penicillin/Streptomycin antibiotics (Corning), and 25 µg/mL plasmocin (Invivogen, San Diego, CA). All cell lines were certified prior to purchase and were subsequently authenticated by morphologic inspection on a regular basis.

### Generation of stable cell lines

Expression constructs of human DARPP-32 and t-DARPP cDNA in pcDNA3.1 were generous gifts from Dr. Wael El-Rifai at the University of Miami in Florida.^17^ The Flag-tagged coding sequences of DARPP-32 and t-DARPP were subcloned into the retroviral vector, pMMP. The pMMP vector and its corresponding pMMP-LacZ control construct were kindly provided by Dr. Debabrata Mukhopadhyay at Mayo Clinic in Jacksonville, Florida.^35^ Retrovirus was produced by transfecting 293T cells with packaging plasmids and pMMP vectors encoding DARPP-32 isoforms. The retrovirus was collected at 48 and 72 h post-transfection and concentrated using Retro-X concentrator (Takara, Mountain View, CA) according to the manufacturer’s protocol. The concentrated virus was then used to transduce DMS-53 and H1048 cell lines as previously described.^36^

Control pLKO.1-LacZ shRNA and lentiviral shRNA pLKO.1 plasmids designed to silence DARPP-32 and ASCL1 protein expression were purchased from Sigma-Aldrich. To prepare the lentivirus, shRNA pLKO.1 plasmids along with their corresponding packaging plasmids were transfected in 293T cells. After collecting the media at 48- and 72-h post-transfection, the lentivirus was concentrated using Lenti-X concentrator (Takara) according to the manufacturer’s protocol. DMS-53 and H1048 cells transduced with concentrated lentivirus were used for experiments after 72 hours of puromycin (Sigma-Aldrich) selection.

To determine tumour growth in orthotopic murine models, DMS-53 and H1048 SCLC cells were transduced with lentivirus containing luciferase genes. Briefly, MSCV Luciferase PGK-hygro plasmid obtained as a gift from Dr. Scott Lowe (Addgene plasmid #18782; http://n2t.net/addgene:18782; RRID:Addgene_18782) was transfected in 293T cells with its corresponding packaging plasmids to generate lentivirus. Lenti-X concentrator (Takara) was used to concentrate lentivirus using media at 48 and 72 h post transfection. After transduction with lentivirus following 72 hours of hygromycin (Sigma-Aldrich) selection, luciferase-labelled stable human SCLC cells were used for experiments.

### Antibodies and reagents

Antibodies (200 µg/ml) purchased from Santa Cruz Biotechnology (SCBT, Dallas, TX) were used to detect DARPP-32 (Polyclonal; Cat no.: sc-11365; Dilution 1:100 and monoclonal; Cat no.: sc-398360; Dilution 1:200) and α-Tubulin (Cat no.: sc-5286; Dilution 1:500). Polyclonal DARPP-32 antibody was used to detect DARPP-32 isoforms in the PARP-I and Caspase-3 immunoblotting study and to generate all DARPP-32 immunohistochemistry data. Alternatively, all remaining immunoblotting experiments were conducted using the monoclonal DARPP-32 antibody. Antibodies (1 µg/µl) against PARP-I (Cat no.: 9542; Dilution 1:1000), Caspase-3 (Cat no.: 9662; Dilution 1:1000), Cleaved Caspase-3 (Cat no.: 9664; Dilution 1:1000), phosphorylated Akt (S473; Cat no.: 4060; Dilution 1:1000), total Akt (Cat no.: 4691; Dilution 1:1000), phosphorylated p44/42 MAPK (T202/Y204; Cat no.: 4370; Dilution 1:1000) and total p44/42 MAPK (Cat no.: 4695; Dilution 1:1000) were obtained from Cell Signaling Technology (Danvers, MA). Horseradish peroxidase (HRP)-conjugated anti-rabbit (Cat no.: 7074; Dilution 1:5000) and anti-mouse (Cat no.: 7076; Dilution 1:5000) secondary antibodies (1 µg/µl) purchased from Cell Signaling Technology were used to detect primary antibodies in immunoblotting studies. Human recombinant tumour necrosis factor (TNF)-related apoptosis-inducing ligand (TRAIL) was purchased from BioVision Inc. (Milpitas, California).

### Immunoblotting

DMS-53 and H1048 human SCLC cells lysed in RIPA buffer (Millipore) containing protease inhibitor cocktail (Roche, Mannheim, Germany) and phosphatase inhibitor (Millipore) were separated via 4–20% gradient SDS-PAGE (Bio-Rad, Hercules, CA) and transferred to polyvinyl difluoride membranes (PVDF; Millipore). Membranes blocked with 5% bovine serum albumin (Sigma-Aldrich) were incubated with primary and secondary antibodies overnight and for 2 h, respectively. Antibody-reactive protein bands were detected by adding chemiluminescence substrate (Thermo Fisher Scientific, Rockford, IL) to the membrane in the dark. The chemiluminescence output was captured electronically using an ImageQuant™ LAS 4000 instrument (GE Healthcare, Chicago, IL).

### Cell growth assay

To measure cell growth by time lapse, 5x10^3^ human SCLC cells were seeded in a 96-well cell culture plate (Cat no.: 655180; Greiner Bio-One, Frickenhausen, Germany). After allowing cells to adhere during incubation at 37 °C for 4 h, plates were placed in the IncuCyte S3 Live-Cell Analysis System (Essen BioScience, Ann Arbor, MI). Images of each well were captured with a ×4 magnification objective at 8-h intervals for up to 72 h. Captured images were binarised and further processed using IncuCyte S3 2018B software (Essen BioScience). The segmentation threshold was set to 0.8 to achieve the steepest gradient in intensity between background and foreground cells. Cell confluency percentage was calculated and plotted against time of incubation.

### Cell survival assay

Human SCLC cell lines plated in a 96-well microplate at a concentration of 5 × 10^3^ cells/well were used to determine cell viability after 72 h of incubation using CellTiter 96^®^ AQueous One System (Promega, Madison, WI). Epoch microplate spectrophotometer (BioTek, Winooski, VT) was utilised to record absorbance at 490 nm. The absorbance reading was normalised and plotted as percentage of total viable cells. The average of three independent experiments has been reported.

### Apoptosis analysis

DMS-53 cells transduced with DARPP-32 shRNAs or exogenously overexpressed DARPP-32 isoforms were stained with Annexin V-FITC antibodies (BD Biosciences, San Jose, CA) to assess apoptosis. To distinguish early apoptotic cells (Annexin-positive and propidium-iodide-negative) from late apoptotic cells (Annexin-positive and propidium-iodide-positive), cells were exposed to propidium iodide (BD Biosciences). Cells undergoing apoptotic cell death were quantified by Annexin V-FITC positivity using flow cytometry. Flow cytometry analysis was performed using FlowJo software.

### BrdU proliferation assay

LacZ control shRNA- or DARPP-32 shRNA-transduced DMS-53 cells were plated at a density of 1 × 10^5^ cells per 60-mm plate. After overnight incubation, 30 µM BrdU (Sigma-Aldrich) containing fresh medium was added to the plate for 30 min. BrdU-labelled cells were then harvested, fixed, and incubated with primary monoclonal mouse antibodies (Roche) that recognise bound BrdU in the DNA. BrdU-positive cells were detected using flow cytometry following incubation with secondary FITC-conjugated goat anti-mouse antibody (Invitrogen, Carlsbad, CA). Subsequently, propidium iodide staining was performed to differentiate between viable and dead cells. FlowJo software was used for analysis. The average of three separate experiments has been documented in the graphs while one representative experiment has been depicted in the main figure.

### Immunohistochemistry

We obtained human small cell lung cancer whole tissue specimens from six SCLC patients at Mayo Clinic in Rochester, MN, in accordance with institutional review board-approved protocols. Formalin-fixed, paraffin-embedded whole tissues were serially sectioned, mounted on glass slides, immunostained using a C-terminal epitope antibody that recognises both DARPP-32 and t-DARPP (Santa Cruz Biotechnology, cat no.: sc-11365; 1:100) as previously described.^8^ To detect nuclei, slides were counterstained with haematoxylin (IHC World, Woodstock, MD). Tissue microarrays that contained human normal lung tissue from six healthy individuals were purchased from US Biomax Inc. (Rockville, MD). Immunohistochemistry was performed using DARPP-32 antibody as described above. A pulmonary pathologist (A.C.R.) scored each lung tumour specimen by observing the intensity and prevalence of DARPP-32 staining under the microscope.

The lungs of euthanised tumour bearing mice were intracardially perfused with PBS, extirpated, and fixed in neutral buffered 10% formalin at room temperature for 48 h. Formalin-fixed lung tissue samples were sent to Mayo Clinic in Scottsdale, Arizona for processing, embedding in paraffin, and sectioning. Sections were deparaffinised, rehydrated, permeabilised, and then subjected to retrieval of antigens to detect DARPP-32 expression using a polyclonal antibody that detects C-terminal epitope of both isoforms. Stable diaminobenzidine (DAB) solution was used as a chromogen substrate. The sections were counterstained with a haematoxylin solution to visualise nuclei.

### RNA-Seq data processing

RNA-Seq data of 29 human SCLC patients and 23 corresponding matched normal lung tissue were downloaded from the European Genome-phenome Archive (EGAS00001000334).^37^ All sequencing reads were mapped to the UCSC human reference genome (GRCh38/hg38) using publicly available HISAT2 software with default parameters.^38^ Mapped reads were sorted and indexed by SAMtools software.^39^ A read summarisation function from Subread software, namely featureCounts, was applied to quantify gene expression using transcript per million (TPM).^40^ Using the same methodology, expression of long and short isoforms of *PPP1R1B* gene was calculated. Differential gene expression analysis was performed with R packages, edgeR^41^ and limma.^42^ The differentially expressed genes (DEGs) with an absolute value of log_2_FC ≥ 0 and FDR ≤ 0.05 were considered for future analysis. DEGs were ranked based on the highest to lowest fold change values (log_2_FC).

### Pathway enrichment analysis

To understand the enriched pathways of the DEGs, Gene Set Enrichment Analysis (GSEA) was performed by GSEA Desktop V3.0 with default parameters using the gene set obtained from c2.cp.kegg.v6.2.symbols.gmt and exported from MSigDB (the Molecular Signatures Database, version 6.2).^43,44^ The RNA-Seq read counts of Notch family members enriched in GSEA were identified using the same methodology described above. A heat map generated in GraphPad Prism 8 software was created by plotting log values of RNA-Seq read counts for denoted genes involved in Notch signalling.

### In vivo orthotopic lung cancer model

Pathogen-free SCID/NCr mice purchased from Charles River Laboratories (Wilmington, MA) were allowed one week to acclimate to their surroundings, bred, maintained under specific pathogen-free (SPF) conditions in a temperature-controlled room with alternating 12-hour light/dark cycles, and fed a standard diet. Eight- to twelve-week-old male and female mice were first anesthetised with pharmaceutical grade ketamine (90–120 mg/kg) and xylazine (5–10 mg/kg) via intraperitoneal injection under a laminar flow hood in an SPF room within the animal facility. Each fully anesthetised mouse was placed in the right lateral decubitus position and the left lateral chest was sterilised. In all, 1 × 10^6^ luciferase-labelled human SCLC cells, DMS-53 or H1048, suspended in an 80 μl PBS and Matrigel^®^ mixture (5:3 ratio; each from Corning) were orthotopically injected into the left thorax of anesthetised mice. Eye lubrication to prevent dry eyes and heating pads to ensure proper body temperature were used during continuously monitored recovery from anaesthesia. To obtain weekly tumour growth assessments, mice were luciferase imaged using an In Vivo Xtreme xenogen imaging system (Bruker, Billerica, MA). Briefly, mice were intraperitoneally injected with 100 μl of 30 mg/ml luciferin (Gold Biotechnology, St. Louis, MO) and transferred to a chamber using 4% isoflurane for anaesthesia induction. Mice were imaged for <2–3 min in the xenogen imaging system, which maintains 1.5–2% isoflurane-induced anaesthesia through nose cones, and closely observed during recovery. Upon completion of the tumour study, mice were euthanised using asphyxiation by CO_2_ inhalation to effect with a flow rate displacing less than 30% of the chamber volume per minute in accordance with Institutional Animal Care and Use Committee (IACUC) euthanasia guidelines and consistent with recommendations of the Panel of Euthanasia of the American Veterinary Medical Association. Following euthanasia, lungs were perfused, harvested, and preserved in formalin for immunohistochemistry. Bruker molecular imaging software was used to calculate luciferase intensity (total photons count) of tumour cells in each mouse. Tumour growth was determined by plotting average luciferase intensity over time in GraphPad Prism 8 software. All animal studies were performed in accordance with protocols approved by the University of Minnesota IACUC.

### Transient transfection

Human SCLC cell lines, DMS-53 or H1048, were plated in six-well cell culture plates at a concentration of 1 × 10^5^ cells per well. The following day, cells were washed with PBS and suspended in OPTI-MEM reduced serum medium (Gibco, Grand Island, NY) for transfection. Polyfect transfection reagent (Qiagen) was used to transfect 1 µg of ASCL1 cDNA plasmids in H1048 cells according to the protocols from the manufacturer. After 4 h, antibiotic containing complete RPMI 1640 medium was added to the cells. Cells were harvested, and subsequent experiments were performed 48 h post transfection.

### In vitro promoter luciferase assay

Luciferase reporter of the DARPP-32 promoter was a generous gift from Dr. Wael El-Rifai at University of Miami Health System in Florida.^45^ Briefly, investigators amplified the DARPP-32 promoter region (−96 to −3115 from transcription start site) by PCR and then cloned it into the pGL3-basic vector to produce the pGL3-DARPP-32 promoter luciferase reporter. SCLC cells were seeded in 96-well cell culture plates at a density of 1 × 10^4^ DMS-53 or H1048 cells per well. After 24 hours post-plating, cells were transfected with 500 ng of pGL3-DARPP-32 plasmids and 50 ng of pRL-SV40 plasmids (renilla; internal reference control) using Polyfect transfection reagent (Qiagen). Lastly, cells were washed with PBS (Corning), lysed with supplied cell culture lysis buffer (Promega), and subjected to dual-luciferase assay 48 h post-transfection using Dual-Luciferase Reporter Assay System (Promega). Luminescence signals from firefly luciferase and renilla luciferase were measured in a multiplate reader, namely Synergy Neo 2 (BioTek).

### Site-directed mutagenesis

A pair of partially mismatched primers were designed for each ASCL1 binding sites on DARPP-32 promoter using QuikChange Primer Design software (https://www.agilent.com/store/primerDesignProgram.jsp). Primer sequences are available in Supplementary Table 1. Briefly, pGL3-DARPP-32 plasmids were used as templates in PCR reactions to synthesise plasmids containing mutated ASCL1 binding sites by annealing and amplifying site-directed mutagenesis primers using QuikChange Lightning Site-Directed Mutagenesis Kit (Agilent, Cedar Creek, TX). Digestion with *Dpn* I enzyme was carried out to eliminate methylated parental plasmid strands. Newly synthesised unmethylated plasmids were then transformed into competent *E. coli* XL-10 gold bacteria for nick repair and subsequent amplification. Lastly, plasmids containing mutated ASCL1 binding sites were isolated and verified using Sanger sequencing (Genewiz, Minneapolis, MN).

### Statistics

Statistical comparisons performed with one- or two-way analysis of variance (ANOVA) in GraphPad Prism 8 software were considered significant when values of *P* < 0.05. To make comparisons between groups, Dunnett or Sidak multiple comparison tests were performed in all applicable experiments after one- or two-way ANOVA, respectively. Data are expressed as mean ± SEM and representative of at least three independent experiments.

### Preprint

A previous version of this paper was published as a preprint [10.1101/703975].

## Results

### DARPP-32 and t-DARPP promote small cell lung cancer cell growth and proliferation

**Fig. 1:**
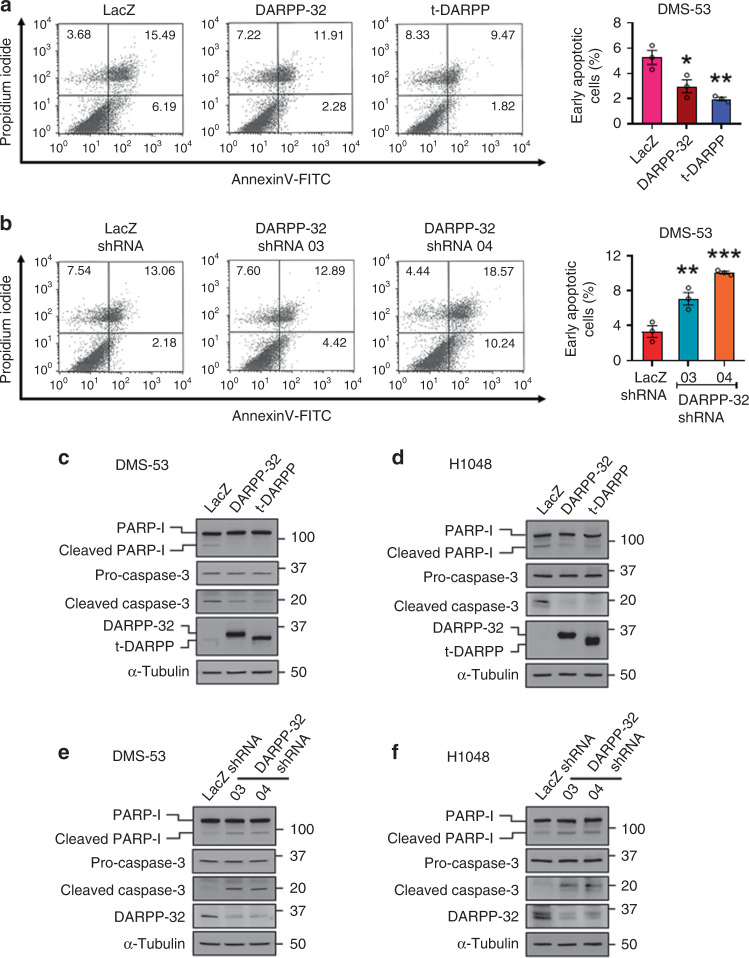
DARPP-32 and t-DARPP decrease cell death by reducing apoptosis. **a** Retrovirus containing control- (LacZ), DARPP-32- or t-DARPP-overexpressing clones and **b** lentivirus encoding control (LacZ) or DARPP-32 shRNAs were transduced in DMS-53 cells. Cells were incubated with anti-annexin V antibodies conjugated with FITC followed by propidium iodide incorporation. Flow cytometry-based apoptosis assays were performed to determine the total number of annexin V-positive cells. The average number of annexin V-positive cells of three independent experiments were plotted in a bar graph. Each open circle on a graph represents an independent experiment. The numerical values on quadrants of the scatter plots represent the percentage of total cells in one single representative experiment. **c** DMS-53 and **d** H1048 cells were transduced with control-, DARPP-32- or t-DARPP-overexpressing clones. Cell lysates were collected and immunoblotted with antibodies to detect cleaved and uncleaved PARP-I, cleaved and uncleaved (i.e., pro-) caspase-3, DARPP-32 and α-tubulin (loading control). **e** DMS-53 and **f** H1048 cells were transduced with lentivirus encoding control or DARPP-32 shRNAs. Cleaved and uncleaved PARP-I, cleaved and uncleaved (i.e., pro-) caspase-3, DARPP-32 and α-tubulin (loading control) proteins were detected by immunoblotting of cell lysates. Immunoblots are representative of three independent experiments. All bar graphs represent mean ± SEM of three independent experiments. *P < 0.05, **P < 0.01 and ***P < 0.001, one-way ANOVA followed by Dunnett’s test for multiple comparison.

Numerous studies have reported the oncogenic role of DARPP-32 isoforms in breast and gastric malignancies.^29,46^ Recently, we demonstrated that overexpression of DARPP-32 isoforms in NSCLC promotes tumour growth.^8^ Based on these findings, we sought to investigate whether DARPP-32 isoforms contribute to the oncogenesis of SCLC. To begin to assess the role of DARPP-32 isoforms in SCLC, we transduced DMS-53 human SCLC cells with retroviral plasmids to overexpress exogenous DARPP-32, t-DARPP, or corresponding control LacZ protein (Fig. 1c and Supplementary Fig. 1a: left panel). To determine how DARPP-32 isoform overexpression affects DMS-53 growth in vitro, we assessed cell confluency over time using the IncuCyte^®^ S3 live cell analysis system and found DARPP-32 isoform overexpression increases the growth rate of DMS-53 cells (Supplementary Fig. 2a). We next sought to determine the effect of DARPP-32 isoforms on cell viability. Colorimetry-based MTS cell viability assays were performed using DMS-53 cells transduced with retrovirus overexpressing DARPP-32 isoforms. DMS-53 cells overexpressing DARPP-32 or t-DARPP protein exhibited increased viability compared to corresponding LacZ-transduced control cells (Supplementary Fig. 3a). We replicated these studies using another human SCLC cell line, H1048. Stable retroviral overexpression of DARPP-32 and t-DARPP in H1048 cells (Fig. 1d and Supplementary Fig. 1b: left panel) resulted in increased cell growth (Supplementary Fig. 2b) and viability (Supplementary Fig. 3b). Next, we silenced endogenous DARPP-32 protein expression through lentiviral shRNA-mediated transduction in DMS-53 cells using two different shRNAs targeting distinct regions of DARPP-32 (Fig. 1e and Supplementary Fig. 1a: right panel). In accordance with our DARPP-32 overexpression findings, we observed decreased DMS-53 growth (Supplementary Fig. 2c) and reduced cell viability (Supplementary Figure 3c) upon DARPP-32 protein ablation using live cell analysis and MTS1-based cell viability assays, respectively. Similarly, shRNA-mediated DARPP-32 protein knockdown in H1048 cells (Fig. 1f and Supplementary Fig. 1b: right panel) resulted in a reduced cellular growth rate (Supplementary Fig. 2d) and fewer viable cells (Supplementary Fig. 3d) relative to corresponding LacZ shRNA transduced control SCLC cells. Collectively, our findings suggest DARPP-32 and t-DARPP positively regulate SCLC cell growth and proliferation.

### DARPP-32 isoforms negatively regulate apoptosis

Given that DARPP-32 isoforms promote SCLC cell growth and proliferation, we sought to determine whether DARPP-32 isoforms regulate apoptosis in SCLC cells. We first performed flow cytometry-based annexin V apoptosis assays in DMS-53 cells retrovirally transduced with control-, DARPP-32- or t-DARPP-overexpressing vectors. We observed a reduced number of annexin V-positive cells (Fig. 1a: lower right quadrant; annexin V-FITC positive and propidium iodide negative) in DARPP-32- and t-DARPP-overexpressing DMS-53 cells, which suggests DARPP-32 isoform overexpression causes a reduction in apoptosis (Fig. 1a: right). We next assessed apoptosis in DARPP-32-depleted DMS-53 cells using flow cytometry-based annexin V assays. As expected, DARPP-32 ablation caused an increase in annexin V-positive cells relative to controls (Fig. 1b). To determine how DARPP-32 regulates the expression of apoptosis-associated proteins, we performed immunoblotting studies in human DMS-53 and H1048 SCLC cells overexpressing DARPP-32 isoforms or corresponding control LacZ. Briefly, caspase proteins are cysteine-dependent aspartate-specific proteases that play central role in mediating apoptosis. An initiator caspase (e.g. Caspase-8, -9) proteolytically cleaves an effector caspase (e.g. Caspase-3, -6) upon binding to specific oligomeric activator protein (e.g. Apaf-1). The active effector caspases then proteolytically degrade a host of intracellular proteins (e.g. PARP-I) to carry out the cell death programme.^47^ We observed reduced expression of cleaved poly (ADP-ribose) polymerase-I (PARP-I) and caspase-3 proteins in DARPP-32- and t-DARPP-overexpressing cell lines relative to controls (Fig. 1c, d), suggesting exogenously overexpressed DARPP-32 isoforms decrease apoptosis in SCLC cells. Conversely, shRNA-mediated depletion of DARPP-32 isoforms resulted in increased caspase-3 and PARP-I cleavage in DMS-53 (Fig. 1e) and H1048 (Fig. 1f) human SCLC cells, which is indicative of increased apoptosis upon silencing of DARPP-32 proteins. We next sought to explore the pro-survival role of DARPP-32 isoforms in human SCLC cells. We challenged human SCLC DMS-53 cells with TNF-related apoptosis-inducing ligand (TRAIL) and measured cleaved PARP-I and caspase-3 expression. It has been shown that knockdown of endogenous DARPP-32 increases TRAIL-induced apoptosis in gastric cancer cell lines through activation of SRC/STAT3 signalling,^48^ which contributed to our rationale for selecting TRAIL as an inducer of apoptosis. In line with the previous study,^48^ we observed that overexpression of DARPP-32 and t-DARPP proteins protects SCLC cells from TRAIL-induced apoptosis based on our immunoblotting results showing reduced caspase-3 and PARP-I cleavage in TRAIL-treated DMS-53 cells overexpressing DARPP-32 or t-DARPP relative to LacZ control cells (Supplementary Fig. 4). In summary, DARPP-32 isoforms promote SCLC cell survival and negatively regulate apoptosis under basal conditions and in the presence of an inducer of apoptosis.

### Akt/Erk-mediated cell proliferation is regulated by DARPP-32 in SCLC

**Fig. 2:**
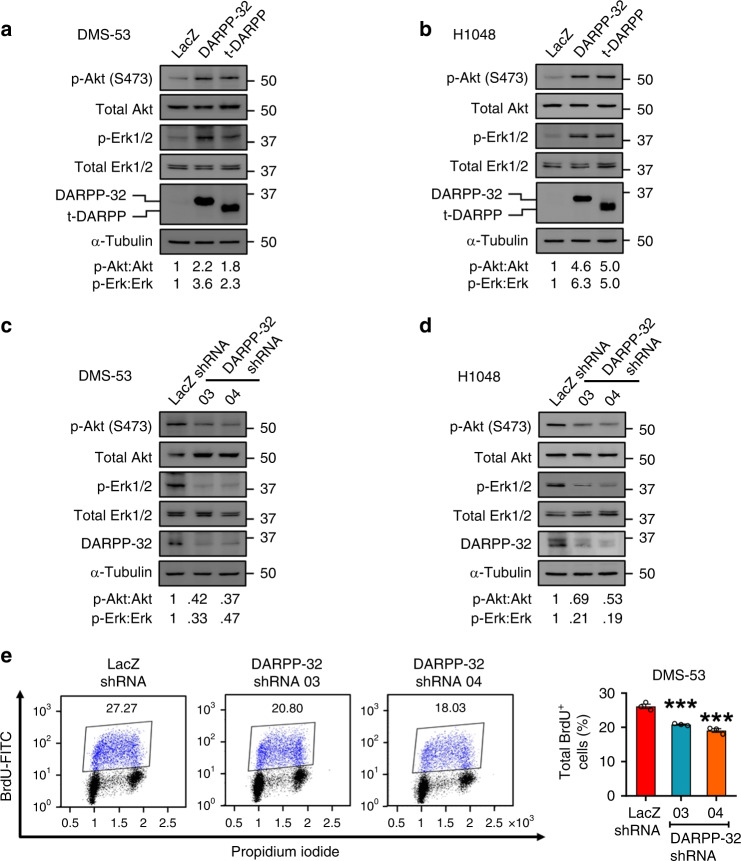
DARPP-32 and t-DARPP positively regulate cell survival through Akt and Erk1/2. **a** Lysates of DMS-53 and **b** H1048 cells overexpressing control, DARPP-32 or t-DARPP clones were immunoblotted using antibodies against phosphorylated Akt (p-Akt; S473), total Akt, phosphorylated Erk1/2 (p-Erk1/2, T202/Y204), total Erk1/2, DARPP-32 and α-tubulin (loading control). **c** DMS-53 and **d** H1048 cells transduced with control or DARPP-32 shRNAs were subjected to western blotting using antibodies against phosphorylated Akt (p-Akt; S473), total Akt, phosphorylated Erk1/2 (p-Erk1/2), total Erk1/2, DARPP-32 and α-tubulin (loading control). All Immunoblots are representative of three independent experiments. Densitometry analysis was performed using Image J software and numerical values are reported below each immunoblot. **e** Flow cytometry-based BrdU cell proliferation assays were performed in DMS-53 cells transduced with control or DARPP-32 shRNAs following incubation with anti-BrdU antibodies conjugated with FITC. DNA intercalating fluorescent agent (propidium iodide) was used to quantify total DNA in the cells. The bar graph represents mean ± SEM of at least 3 independent experiments. ***P < 0.001, one-way ANOVA followed by Dunnett’s test for multiple comparison.

Based on prior findings that Akt and Erk1/2 signalling pathways contribute to DARPP-32 mediated breast and gastric oncogenesis, we sought to elucidate their potential role in SCLC tumour growth. We first evaluated the phosphorylation status of Akt and Erk in human SCLC cells by immunoblotting. Stable retroviral overexpression of exogenous DARPP-32 and t-DARPP substantially enhanced phosphorylation levels of Akt and Erk in DMS-53 and H1048 cells (Fig. 2a, b). Corresponding total Akt and Erk1/2 protein levels in SCLC cells overexpressing DARPP-32 isoforms remained consistent by immunoblotting (Fig. 2a, b). We next performed immunoblotting studies using human DMS-53 and H1048 SCLC cells transduced with lentivirus encoding DARPP-32 shRNAs or control LacZ shRNA. We found ablation of endogenous DARPP-32 decreases phosphorylation levels of Akt and Erk1/2 relative to LacZ shRNA-transduced controls, while the expression of corresponding total Akt and Erk1/2 protein remains unchanged upon DARPP-32 modulation (Fig. 2c, d). Given Akt and Erk activation promote cell proliferation,^8^ we next performed flow cytometry-based BrdU cell proliferation assays in DMS-53 cells transduced with DARPP-32 or control shRNA. We observed reduced BrdU staining in DARPP-32-depleted DMS-53 cells (Fig. 2e), suggesting DARPP-32 isoforms activate Akt/Erk signalling and regulate SCLC cell proliferation.

### DARPP-32 and t-DARPP promote SCLC growth in orthotopic mouse xenograft models

**Fig. 3:**
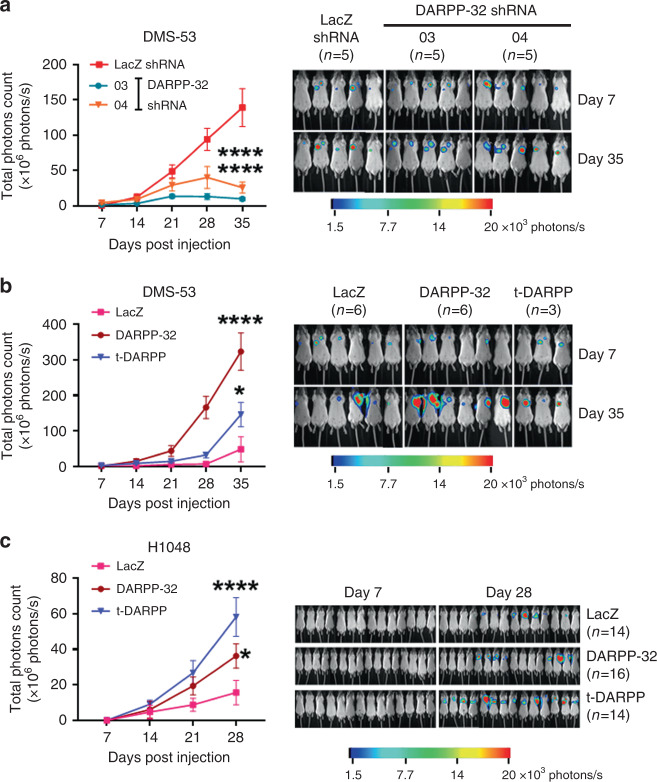
DARPP-32 isoforms promote growth of human SCLC cells in mouse xenograft models. **a** Luciferase-labelled human DMS-53 cells transduced with lentivirus encoding control (LacZ) or DARPP-32 shRNAs were orthotopically injected into the left thorax of SCID mice and imaged for luminescence on indicated days. **b** Luciferase-labelled human DMS-53 and **c** H1048 cells overexpressing control (LacZ), DARPP-32 or t-DARPP clones were injected into the left thorax of SCID mice and imaged for luminescence on the indicated days. In vivo images of SCID mice bearing luciferase-labelled tumours showing luminescence on the indicated days are depicted. Total luminescence intensity (photons count) was calculated using molecular imaging software. The numerical value of luminescence was represented by the colour bar. The average luminescence intensity of each time point was plotted. Error bars indicate SEM. *P < 0.05 and ****P < 0.0001, one-way ANOVA followed by Dunnett’s test for multiple comparison.

Based on our results that SCLC cell growth and proliferation is regulated by DARPP-32 isoforms, we sought to determine whether DARPP-32 drives SCLC growth in vivo. To this end, we utilised an orthotopic lung cancer xenograft mouse model.^8^ Specifically, we injected luciferase-labelled DARPP-32-depleted human DMS-53 SCLC cells into the left thorax of anesthetised SCID mice. After establishment of the lung tumour, we xenogen-imaged the mice regularly over the course of 5 weeks. Mice challenged with DARPP-32-ablated DMS-53 cells showed a substantial decrease in lung tumour growth compared to mice challenged with human SCLC cells transduced with control LacZ shRNA (Fig. 3a) suggesting that DARPP-32 promotes human SCLC growth in mouse xenograft models. We next sought to determine whether overexpression of DARPP-32 isoforms promotes SCLC tumour growth in vivo. Briefly, 1 × 10^6^ luciferase-labelled human DMS-53 SCLC cells stably overexpressing exogenous DARPP-32 or t-DARPP were injected into the left thorax of anesthetised SCID mice, and we observed increased tumour growth in mice harbouring DMS-53 cells overexpressing DARPP-32 or t-DARPP relative to mice that received control DMS-53 cells overexpressing LacZ (Fig. 3b). Correspondingly, we demonstrated increased tumour growth in mice challenged with H1048 cells overexpressing DARPP-32 and t-DARPP proteins (Fig. 3c). To confirm DARPP-32 overexpression in xenografted tumour tissues, we performed immunohistochemistry studies using a C-terminal epitope antibody that detects both DARPP-32 and t-DARPP proteins. As expected, human DMS-53 xenografts overexpressing either DARPP-32 or t-DARPP showed high DARPP-32 isoform expression compared to DMS-53 LacZ control xenografts (Supplementary Fig. 5a). Additionally, by immunohistochemistry we observed less DARPP-32 expression in DARPP-32-depleted human DMS-53 xenografts relative to LacZ shRNA-transduced controls (Supplementary Fig. 5b). Taken together, our data suggest DARPP-32 isoforms promote SCLC growth in orthotopic mouse xenograft models.

### DARPP-32 isoforms are overexpressed in SCLC tumours, but undetectable in normal lung tissue

**Fig. 4:**
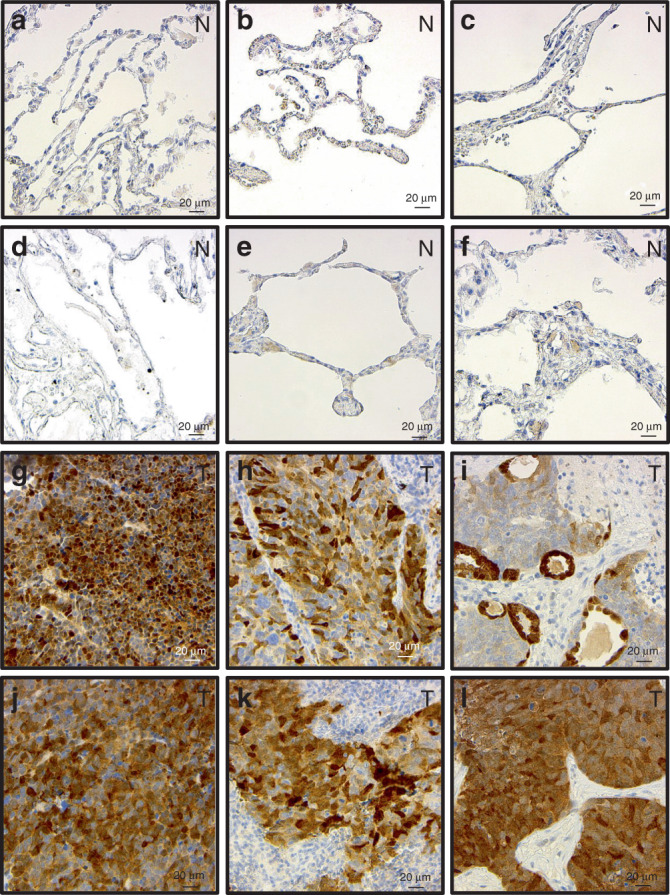
Expression of DARPP-32 isoforms is elevated in human SCLC patients. **a–f** Normal lung tissues (*n* = 6) and **g–l** tumour tissues from SCLC patients (*n* = 6) were subjected to immunohistochemistry using an antibody that recognises both DARPP-32 and t-DARPP isoforms. Nuclei were counterstained using haematoxylin stain. N and T denotes normal and tumour, respectively. Scale bar indicates 20 µm.

Given that DARPP-32 ablation reduces tumour growth in mouse models of human SCLC, we aimed to elucidate the clinical relevance of DARPP-32 in SCLC patients. Based on published reports that indicate upregulation of DARPP-32 and t-DARPP have been associated with breast, gastric, colorectal and non-small cell lung cancer,^29,46^ we sought to assess DARPP-32 and t-DARPP protein expression in SCLC patients. Immunohistochemistry was performed to detect DARPP-32 isoforms in tissue specimens obtained from small cell lung carcinoma patients. Briefly, we obtained serial whole tissue sections of formalin-fixed paraffin embedded tumour tissue blocks corresponding to each patient and immunostained with an antibody that detects both DARPP-32 and t-DARPP via a C-terminal epitope present in both isoforms. Using the same antibody, we also immunostained normal human lung tissue specimens (antibody specificity controls are shown in Supplementary Fig. 6a, b). We observed strong expression of DARPP-32 isoforms in human SCLC specimens, whereas DARPP-32 and t-DARPP protein expression was virtually undetectable in physiologically normal human lung tissue (Fig. 4). The percentage of tumour cells positive for DARPP-32 isoforms and their staining intensity was scored by a pulmonary pathologist (A.C.R.) using a scale of 0–3 (i.e. 0 = none, 1 = weak, 2 = moderate, 3 = strong expression). Based on the percentage of tumour cells staining positive and the staining intensity in those cells, we calculated an immunohistochemistry score (IHC score = percentage of tumour cells × staining intensity) for each specimen using the pathological scoring (Supplementary Table 2). Specifically, we observed strong DARPP-32 staining intensity (i.e. 3) in all six SCLC patient-derived specimens examined, and the percentage of DARPP-32 positive tumour cells was high (i.e. ≥80%) in four of six SCLC tissues and detectable (i.e. 10–30%) in the other two samples (Fig. 4g–l and Supplementary Table 2). While these immunostaining studies of lung specimens derived from six SCLC patients and six healthy individuals lack a sufficiently large number of samples to draw definitive conclusions, our initial pilot data suggests DARPP-32 isoforms are aberrantly overexpressed in human SCLC and additional comprehensive immunohistochemistry studies are warranted.

### Transcripts associated with Notch signalling are upregulated in a subset of SCLC tumours expressing high levels of t-DARPP

**Fig. 5:**
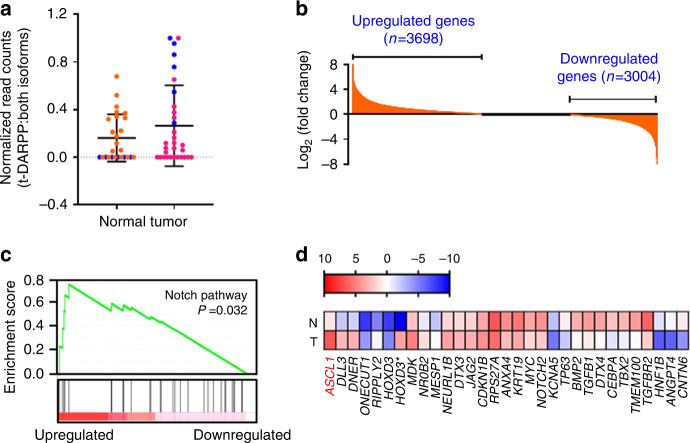
Transcripts associated with Notch signalling are upregulated in a subset of SCLC tumours expressing high levels of t-DARPP. **a** Expression of t-DARPP transcripts was quantified using a previously published RNA-Seq database.^37^ Blue points indicate individual SCLC patients (*n* = 6) with an elevated t-DARPP expression (t-DARPP: both DARPP-32 isoforms) in tumour tissue compared to mRNA derived from corresponding adjacent normal tissue. **b** Quantification of mRNA transcripts in a subset of SCLC patients (*n* = 6; blue points) expressing high level of t-DARPP isoforms in tumour relative to normal tissue. **c** Gene set enrichment analysis (GSEA) was performed to identify cellular pathways regulated in a subset of SCLC patients with elevated t-DARPP levels in tumour tissue. Notch signalling was enriched by GSEA. **d** Heat map showing the expression of Notch family member genes identified through GSEA. N and T denotes normal and tumour tissue, respectively. Red colour represents highest fold change in expression in tumour tissue relative to normal. Colour bar represents normalised read counts in a log scale.

We sought to elucidate the molecular mechanisms through which DARPP-32 isoforms promote human SCLC. First, we assessed DARPP-32 and t-DARPP transcript levels in a RNA-Seq dataset consisting of 29 human SCLC patients, 23 of which also contained corresponding matched normal lung tissue.^37^ We found that t-DARPP transcripts were elevated in SCLC relative to normal lung tissue (Fig. 5a). Interestingly, we identified six SCLC patients within this cohort with high levels of t-DARPP transcript in tumour tissue, but undetectable t-DARPP in corresponding normal tissue (blue data points in Fig. 5a). Using differential gene expression to analyse the high tumoural t-DARPP subset, we identified 3698 upregulated genes (fold change threshold ≥ 1.7) and 3004 downregulated genes (fold change threshold ≤ 0.55) in tumour relative to matched normal tissue (Fig. 5b and Supplementary Tables 3 and 4). Based on GSEA, we identified five cellular pathways that are correlated with high t-DARPP expression in human SCLC: Notch, bone morphogenic protein (BMP), calcium, G protein coupled receptor (GPCR), and mitogen-activated kinase (MAPK; Fig. 5c, Supplementary Fig. 7, Supplementary Tables 5 and 6). We chose to focus our investigation on whether genes associated with Notch signalling control regulation of t-DARPP based on the well-established role of Notch family members in SCLC. To that end, we examined differential tumour vs. normal expression of 31 Notch signalling associated transcripts that comprised the gene set (Supplementary Table 5). We found that achaete-scute homologue 1 (ASCL1), a main driver of SCLC, was increased nearly 50-fold in tumour tissues compared to matched normal specimens (Fig. 5d, Supplementary Table 5). Physiologically, ASCL1 is predominantly expressed in neural progenitor cells and supports growth and development of nervous tissue.^49^ Pathologically, ASCL1 is overexpressed in SCLC and acts as a neuroendocrine lineage-specific oncogene whose expression is inhibited by active Notch signalling.^50^ Most ASCL1-high SCLCs (i.e. SCLC-A molecular subtype) exhibit a gene expression profile suggestive of low Notch pathway activity and high expression of DLK1, a non-canonical inhibitor of Notch signalling.^31,51^

### ASCL1 transcriptionally regulates DARPP-32 expression

**Fig. 6:**
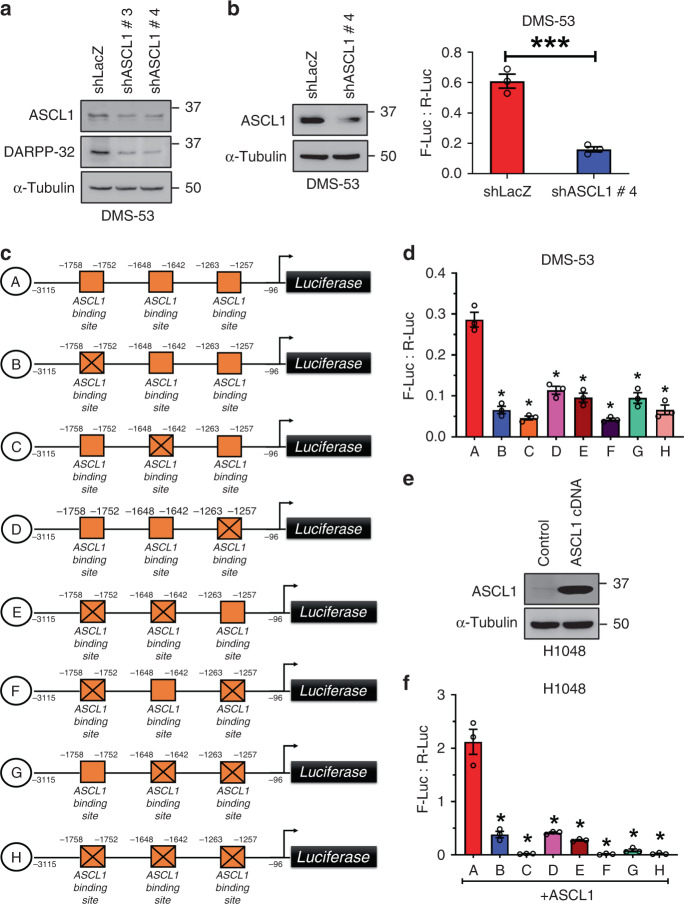
ASCL1 positively regulates DARPP-32 expression in SCLC cells. **a** DMS-53 cells stably transduced with lentivirus encoding either LacZ shRNAs (control) or ASCL1 shRNAs (clone #3 and #4) were lysed using RIPA cell lysis buffer. Immunoblotting was performed with antibodies against ASCL1, DARPP-32 and α-Tubulin (loading control). Immunoblot is representative of three independent experiments. **b** Dual-luciferase assays (right) were performed in DMS-53 cells transduced with either control or ASCL1 shRNAs following transient transfection of pGL3-DARPP-32-Luc (Firefly) and pRL-SV40 (Renilla) plasmids. Firefly and renilla luminescence were measured and plotted as a ratio. The bar graph represents mean ± SEM of three independent experiments, two-tailed unpaired *t*-test. Immunoblotting (left) was performed in parallel to confirm shRNA-mediated knockdown of ASCL1 in DMS-53 cells. **c** Three potential ASCL1 binding sites are present within the DARPP-32 promoter (orange boxes). ASCL1 binding sites on the DARPP-32 promoter have been mutated by site-directed mutagenesis. The crossed boxes refer to the mutated sites. **d** DMS-53 cells transfected with either unmodified pGL3-DARPP-32-Luc plasmids (A) or site-directed mutagenesis pGL3-DARPP-32-Luc constructs (indicated as B to H) were subjected to dual-luciferase assay. **e** Immunoblotting was performed in H1048 cells transfected with ASCL1 expressing plasmids (cDNA). Immunoblotting results represent three independent experiments. **f** Transient transfection of either unmodified pGL3-DARPP-32-Luc plasmids (A) or site-directed mutagenesis pGL3-DARPP-32-Luc constructs (indicated as B to H) was carried out in H1048 cells exogenously overexpressing ASCL1. Dual-luciferase assays were performed after 48 h post-transfection. Each open circle on a graph represents an independent experiment. Bar graph represents an average of three independent experiments. Error bars indicate SEM. *P < 0.0001, one-way ANOVA followed by Dunnett’s test for multiple comparison.

Given ASCL1 is frequently overexpressed in SCLC,^52^ we sought to determine the role of ASCL1 in regulating DARPP-32 expression. We first performed immunoblotting experiments to assess endogenous expression of ASCL1 and DARPP-32 in DMS-53 and H1048 cells. Based on our results, we classified DMS-53 and H1048 cells as ASCL1-positve and -negative cells, respectively (Supplementary Fig. 8). Given that DARPP-32 isoforms (Figs. 4 and 5a) and ASCL1 (Fig. 5d) are both overexpressed in SCLC and ChIP-seq studies suggest ASCL1 may interact with the *PPP1R1B* promoter,^32^ we investigated whether ASCL1 regulates DARPP-32. Upon lentiviral shRNA-mediated ASCL1 stable knockdown in DMS-53 cells, we observed decreased DARPP-32 protein expression (Fig. 6a). We assessed DARPP-32 promoter activity via dual-luciferase assays in the DMS-53 cells transduced with either ASCL1 shRNA or control (LacZ) shRNA. Briefly, the DARPP-32 promoter (−3115 to −95 from transcription start site) was previously cloned into the pGL3 luciferase reporter vector and characterised by El-Rifai and colleagues.^45^ We transfected DMS-53 cells with pGL3-DARPP-32 (firefly luciferase) and pRL-SV40 (renilla luciferase; acts as transfection control) plasmids to assess DARPP-32 promoter activity by measuring luminescence at 48 h post-transfection. We observed a reduction in DARPP-32 promoter activity in ASCL1 ablated DMS-53 cells (Fig. 6b). To understand how ASCL1 regulates DARPP-32 expression, we individually mutated each of three putative ASCL1 binding sites on DARPP-32 promoter (i.e. CAGCTG motif at −1758 to −1752, −1648 to −1642 and −1263 to −1257) using a site-directed mutagenesis approach. We also generated every combination of double and triple mutated ASCL1 binding sites to net a total of seven ASCL1 binding site mutants (Fig. 6c). To test whether the putative ASCL1 binding sites on the DARPP-32 promoter are required for ASCL1-mediated transcriptional activation of DARPP-32, we performed dual-luciferase assays in DMS-53 cells transfected with either wild-type (pGL3-DARPP-32; construct A) or ASCL1 binding site-mutated (constructs B to H) DARPP-32 promoter constructs (Fig. 6c). Mutation of any of the ASCL1 binding sites reduced DARPP-32 promoter activity (Fig. 6d), suggesting these sites play a role in ASCL1-mediated transcriptional regulation of DARPP-32. To further confirm that ASCL1 transcriptionally regulates DARPP-32, we next performed dual-luciferase assays in ASCL1-negative H1048 cells in which we exogenously overexpressed ASCL1 (Fig. 6e). As expected, ASCL1 overexpression in H1048 cells increases wildtype DARPP-32 promoter (A) activity, whereas exogenous ASCL1 has little effect on its mutant counterparts (B to H; Fig. 6f). Taken together, we identify three novel ASCL1 binding sites on the DARPP-32 promoter through which ASCL1 transcriptionally activates DARPP-32.

## Discussion

While growing evidence supports the oncogenic role of DARPP-32 and t-DARPP in a wide variety of adenocarcinomas,^29,46^ we are the first to report that DARPP-32 isoforms promote neuroendocrine oncogenesis, as we observe DARPP-32 and t-DARPP drive SCLC growth through anti-apoptotic mechanisms and pro-survival signalling via Akt and Erk. Neuroendocrine tumours arise from cells of endocrine and neural origin and have been reported in the pancreas,^53,54^ gastrointestinal tract,^53,54^ lung,^55,56^ breast,^57^ genitourinary tract,^58^ liver,^59^ gall bladder,^60^ and glands, including the thymus.^61^ Pulmonary neuroendocrine tumours are categorised as low-grade and high-grade based on clinical and molecular factors.^62^ SCLCs and large cell neuroendocrine carcinomas comprise high-grade neuroendocrine tumours of the lung,^63^ which are characterised by a strong association with smoking, high mitotic indices, rapid growth, metastasis, frequent *TP53* and *RB1* mutations, and poor survival.^2^ High grade neuroendocrine tumours frequently exhibit Notch inactivation and overexpression of achaete-scute homolog 1 (ASCL1), a transcription factor that functions as a master regulator of neuroendocrine programming and acts as a lineage-specific oncogene.^30^ Here, we show that ASCL1 transcriptionally activates DARPP-32 isoforms in human SCLC cells. Correspondingly, we observe aberrantly high expression of DARPP-32 proteins in SCLC patient-derived tumour specimens and undetectable levels in normal lung, a finding supported by our genomic analysis of a matched SCLC tumour and adjacent normal tissue RNA-Seq dataset.^37^ Genetic ablation of DARPP-32 isoforms in orthotopic SCLC xenografts decreases tumour growth, whereas overexpression of DARPP-32 or t-DARPP in human DMS-53 or H1048 SCLC cells orthotopically injected into mice increases SCLC growth. Collectively, our results suggest upregulation of ASCL1 in SCLC cells transcriptionally activates the DARPP-32 promoter contributing to overexpression of DARPP-32 and t-DARPP proteins, which promote SCLC growth through anti-apoptotic and pro-survival signalling.

Given the primary function of DARPP-32 proteins in neurons and their oncogenic role in non-brain cancers,^29^ we hypothesised that DARPP-32 and t-DARPP contribute to neuroendocrine tumour growth in SCLCs. Greengard discovered DARPP-32 as a neuronal phosphoprotein that inhibits PP-1 by mediating dopamine signalling through dopamine D1 and D2 receptors and glutamate signalling through *N*-methyl-d-aspartate receptor (NMDAR).^19,64–66^ The binding of dopamine, glutamate, nicotine, ethanol, cocaine, and amphetamines to these receptors alters the phosphorylation status of DARPP-32, enabling it to regulate neurotransmission by controlling PP-1 activity.^67,68^ Specifically, phosphorylation of DARPP-32 at T34 by PKA inhibits PP-1 activity, whereas Cdk5-mediated phosphorylation of DARPP-32 at T75 prevents PKA from phosphorylating DARPP-32 at T34 resulting in active PP-1.^29^ As a master regulator of PP-1, overexpression of DARPP-32 isoforms in tumour cells enables DARPP-32 and its truncated t-DARPP isoform to control oncogenic cellular functions.^8,14,16,17,29,46,69^ For example, t-DARPP, which lacks T34 and the ability to inhibit PP-1, activates pro-survival Akt signalling in breast^20^ and gastric cancers.^15^ Likewise, we demonstrated that aberrant upregulation of t-DARPP protein in NSCLC patient-derived tumour tissue correlates with increased lung adenocarcinoma tumour growth (i.e. by T staging score) and that DARPP-32 and t-DARPP promote NSCLC growth via IKKα-induced migration and Akt/Erk-mediated tumour cell survival.^8^ Considering our findings in SCLC that DARPP-32 isoforms promote Akt/Erk-mediated proliferation, survival, and protection from apoptosis relative to the collective evidence implicating DARPP-32 and t-DARPP in drug resistance, a future direction beyond the scope of the studies presented here involves investigating the role of DARPP-32 isoforms in SCLC cells acquiring resistance to chemotherapy. Paired chemotherapy-naive and chemotherapy-resistant SCLC patient-derived xenograft mouse models that would be amenable to addressing this future question have been generated and reported.^70^

Generally speaking, both DARPP-32 and t-DARPP isoforms stimulate tumour cell proliferation across a variety of cancer types, which suggests that the cancer-associated domain exists within a shared region of DARPP-32 and t-DARPP proteins.^29^ One notable exception is a report demonstrating that t-DARPP, but not DARPP-32, contributes to trastuzumab resistance in Her2+ breast cancer.^20^ However, another study presented contrasting data showing that t-DARPP and DARPP-32 can each confer resistance to trastuzumab in breast cancer cells.^24^ DARPP-32 exclusively promotes gastric cell resistance to doxorubicin,^71^ but both DARPP-32 and t-DARPP are overexpressed in gastric cancer^9^ and stimulate upregulation of the angiogenesis-inducing protein, angiopoietin 2.^17^ Cell context is likely an important factor in the modulation of t-DARPP and DARPP-32 downstream activities. The vast majority of cancer studies demonstrate that DARPP-32 and t-DARPP share similar oncogenic functions,^29^ which corresponds to our in vitro functional data (Figs. 1 and 2) and orthotopic mouse model results (Fig. 3), suggesting that DARPP-32 and t-DARPP overexpression promotes SCLC growth.

The Notch signalling pathway has a complex role in SCLC, but most frequently acts as a tumour suppressor by negatively regulating neuroendocrine differentiation. Activation of Notch signalling is initiated by the interaction of extracellularly expressed canonical delta-like ligands DLL1, DLL3 and DLL4 and jagged ligands (JAG1 and JAG2) with Notch-1, −2, −3, or −4 receptors expressed on the surface of adjacent cells. Ligand binding stimulates receptor cleavage and release of the Notch intercellular domain (NICD), which associates with nuclear transcriptional effectors to regulate transcription of Notch target genes, including activation of hairy and enhancer of split 1 (HES1) and hairy and enhancer of split-related protein 1 (HEY1), transcriptional repressors of ASCL1. The basic helix-loop-helix transcription factor ASCL1 is expressed in ~75% of SCLCs, and genetic profiling distinguishes “classic” ASCL1-positive SCLC from “variant” SCLC expressing neurogenic differentiation factor 1 (NEUROD1), another neuronal master regulator that stimulates a neuroendocrine target gene set distinct from that of ASCL1. ASCL1 positively regulates pro-oncogenes associated with SCLC progression and survival, such as Bcl2, SRY-box 2 (SOX2), RET, and nuclear factor I B (NFIB). Concordantly with ASCL1 overexpression, Notch is inactivated in most SCLCs by mutations in Notch pathway genes or expression of Notch inhibitors DLK1 and DLL3.^31^ A DLL3-targeted antibody-drug conjugate named Rovalpituzumab tesirine (ROVA-T) was shown to have single-agent anti-cancer activity in DLL3-expressing lung neuroendocrine carcinomas and clinical trials in SCLCs are ongoing.^72–74^ These results exemplify the highly complex role of Notch signalling in SCLC, which may offer multiple therapeutic targets, and underscore the need to more fully understand the mechanisms of ASCL1-regulated DARPP-32 and Notch pathway associated signalling in SCLC.

Here, we identify ASCL1 as a transcriptional regulator of DARPP-32. In line with our observations, it has been reported that ASCL1 (and not NEUROD1) is physically recruited on DARPP-32 promoter in ASCL1-high human SCLC cell lines.^32^ In a subset of SCLC patients with high tumoural t-DARPP expression, we show that the Notch signalling gene set was highly regulated, among which ASCL1 was the top differentially expressed transcript. We demonstrate that ASCL1 transcriptionally activates DARPP-32 using reporter assays, site-directed mutagenesis of ASCL1 binding sites on the well-characterised DARPP-32 promoter,^45^ and ASCL1 modulation. Molecular crosstalk between DARPP-32 isoforms and the Notch signalling pathway in the context of cancer has yet to be reported. However, Notch/RBP-J signalling plays a key role in the regulation of dopamine responsive as neuron-specific loss of Notch/RBP-J signalling has been shown to cause a deficit in dopamine-dependent instrumental avoidance learning and hyper-responsiveness to apomorphine and SKF38393, a D1 agonist.^75^ Brain-derived neurotrophic factor (BDNF) transcriptionally regulates DARPP-32 expression in neurons, as exemplified by delayed and decreased neuronal DARPP-32 expression in BDNF knockout mice, a phenotype that could be rescued ex vivo through the addition of BDNF to cultured neurons derived from BDNF null mice.^76^ BDNF and ASCL1 were dysregulated and accompanied by aberrant expression of DARPP-32, one of their common transcriptional targets, in striatal neural stem cells derived from a hypoxanthine-guanine phosphoribosyltransferase (HPRT) knockout mouse model of Lesch-Nyhan Syndrome, a neurodevelopmental disorder caused by mutations in the gene encoding HPRT.^77^ Based on prior reports in neurons and our evidence in SCLC that ASCL1 regulates DARPP-32 expression, one could surmise that DARPP-32 serves a modulator of neuroendocrine signalling through its control of protein phosphatase activity. Additional studies are necessary to better understand the functional consequences of ASCL1-regulated DARPP-32 activation and to precisely define how DARPP-32 activates downstream signalling, such as Akt, in the context of neuroendocrine tumorigenesis. There is strong collective evidence that t-DARPP activates Akt phosphorylation in cancer, but identification of DARPP-32 and t-DARPP binding partners is necessary to understand exactly how t-DARPP activates Akt through S473 phosphorylation.^29^

SCLC represents a particularly aggressive and deadly form of lung cancer. While the addition of immunotherapy to standard chemotherapy has shown recent promise in the treatment of extensive-stage SCLC,^78^ identifying targetable driver mutations and oncogenic pathways in SCLC as the basis for effective molecular targeted therapies has proven challenging.^52^ A better molecular understanding SCLC progression will provide a foundation for the discovery of new prognostic tools and therapeutic approaches to improve the clinical outlook and quality of life of patients afflicted with this deadly disease. Here we provide new molecular insights into the mechanisms of SCLC growth. We show that DARPP-32 and t-DARPP promote SCLC proliferation, evasion from caspase-3-dependent apoptosis, activation of Akt and Erk, and increased tumour growth in mice receiving lung xenografts of human SCLC cells stably transduced to overexpress or ablate DARPP-32 isoforms. Patient-derived SCLC tumour specimens exhibit aberrantly high DARPP-32 and t-DARPP protein expression relative to normal lung, in which DARPP-32 isoforms are virtually undetectable by immunostaining. Based on the clinical significance of DARPP-32 and t-DARPP in lung, breast, gastric and other cancers as well as their role in Akt activation and drug resistance, DARPP-32 isoforms may represent a potential therapeutic target.^29^ Furthermore, the cancer-specific expression profile of DARPP-32 isoforms lends itself well to safe targeting and minimal toxicity to non-malignant tissue. In order to develop effective pharmacological inhibitors that target the shared region of DARPP-32 and t-DARPP proteins, the tertiary structure of t-DARPP must be determined. To date, tertiary structures of DARPP-32 isoforms have not been reported because their acidic glutamine- and aspartate-rich central regions likely hinder crystallisation of their elongated monomer secondary structure, consisting of 12% α helices, 29% β strands, 24% β turns and 35% random coils.^79^ However, advances in single-particle cryo-electron microscopy have demonstrated that proteins considerably smaller than the theoretical limit of 50 kDa for cryo-EM can be visualised at near-atomic resolution (i.e. 3.5–5 Å), suggesting it may now be technically feasible to solve the tertiary structure of DARPP-32/t-DARPP.^80^ Other future directions that will expedite exploration of targeting DARPP-32 and/or t-DARPP signalling as a potential anti-cancer therapy include understanding the exact mechanism(s) through which DARPP-32 isoforms activate Akt and identification of proteins that bind directly to DARPP-32 and/or t-DARPP. It is currently unknown whether most identified upstream and downstream signalling molecules interact with DARPP-32 isoforms directly or indirectly.^29^ These future opportunities, the collective prior studies reporting the role of DARPP-32 and t-DARPP in oncogenesis and resistance to therapy, and our findings that upregulation of DARPP-32 isoforms in human SCLC promote growth, anti-apoptotic mechanisms and pro-survival signalling in lung tumour cells of neuroendocrine origin underscore the promise of pursuing DARPP-32 and t-DARPP as potential therapeutic targets or prognostic indicators^81^.

## Supplementary information

Supplementary Figures 1-8 & Supplementary Tables 1-6

## Data Availability

Data pertaining to this study are included in the manuscript and Supplemental Tables. Additional datasets used and/or analysed during the current study are available from the corresponding author upon request.
