# Supplementary material for: ASCL1-regulated DARPP-32 and t-DARPP stimulate small cell lung cancer growth and neuroendocrine tumour cell proliferation
Source: Br J Cancer. 2020 Jun 5;123(5):819–32. doi: 10.1038/s41416-020-0923-6 (PMC7463034; doi:10.1038/s41416-020-0923-6)
Supplement: Supplementary file 1 — Supplementary Figures 1-8 & Supplementary Tables 1-6 [file 41416_2020_923_MOESM1_ESM.pdf]

Supplementary Figures and Tables:

Supplementary Figures 1-8:	p. 2 - 9
Supplementary Table 1: List of primers used in this study	p. 10
Supplementary Table 2: Immunostaining scores of patient-derived human SCLC tissue samples	p. 11
Supplementary Table 3: List of upregulated genes with fold change relative to normal	p. 12 - 84
Supplementary Table 4: List of downregulated genes with fold change relative to normal	p. 85 - 148
Supplementary Table 5: List of Notch pathway genes enriched in GSEA	p. 149
Supplementary Table 6: List of genes from 4 pathways enriched in GSEA	p. 150 - 161

Supplementary Figure 1

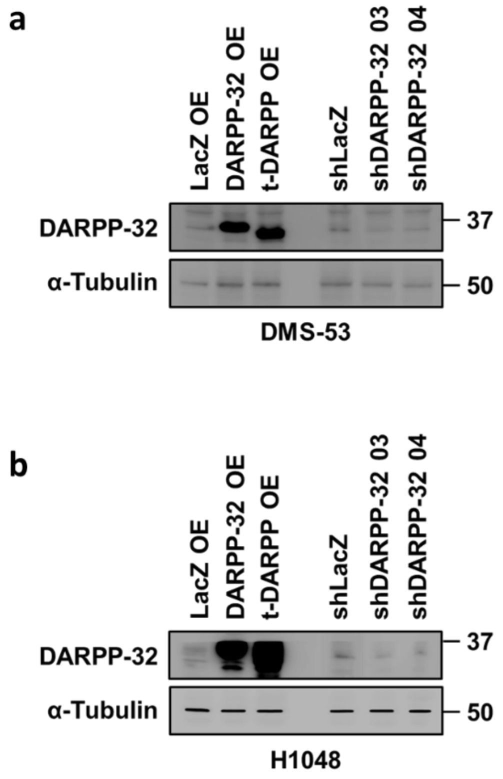

Supplementary Figure 1: Depletion and overexpression of DARPP-32 isoforms in SCLC cells. **a-b** DMS-53 (**a**: left 3 lanes) and H1048 cells (**b**: left 3 lanes) transduced with retrovirus encoding LacZ (control), DARPP-32, and t-DARPP cDNAs were subjected to western blot using antibodies against DARPP-32 and α-Tubulin (loading control). Cell lysates from DMS-53 (**a**: right 3 lanes) and H1048 (**b**: right 3 lanes) cells transduced with shRNA encoded to knockdown LacZ or DARPP-32 (i.e. shDARPP-32 03 and shDARPP-32 04 represent two distinct shRNAs) were used to detect DARPP-32 expression.

Supplementary Figure 2

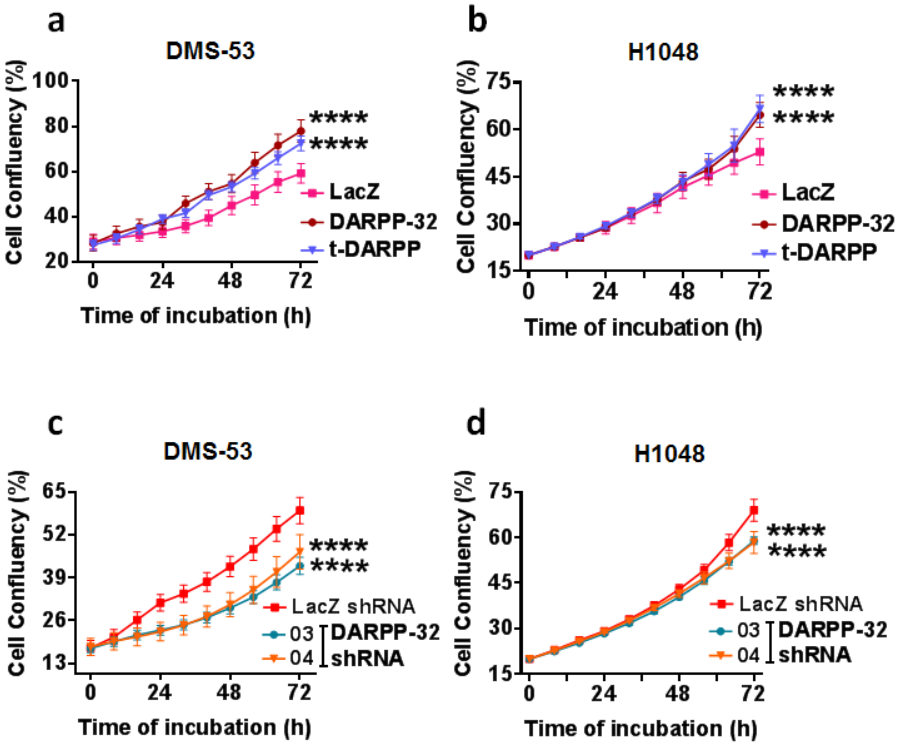

Supplementary Figure 2: DARPP-32 isoforms promote cell growth and proliferation. a-b DMS-53 (a) and H1048 (b) cells expressing control-, DARPP-32- or t-DARPP-overexpressing clones were seeded into 96-well cell culture plates. Images were captured at every 6h interval using IncuCyte® live cell analysis imaging system. Cell confluency was determined by measuring the area occupied by the cells in each well. **c-d** DMS-53 (c) and H1048 (d) cells were transduced with lentivirus encoding control shRNA (LacZ) or DARPP-32 shRNAs (clone numbers 03 and 04). The cell-occupied area in each well was assessed using the IncuCyte® live cell analysis imaging system. The line graph represents average values of three independent experiments. Error bars indicate SEM. ****P<0.0001, 2-way ANOVA followed by Dunnett's test for multiple comparison.

Supplementary Figure 3

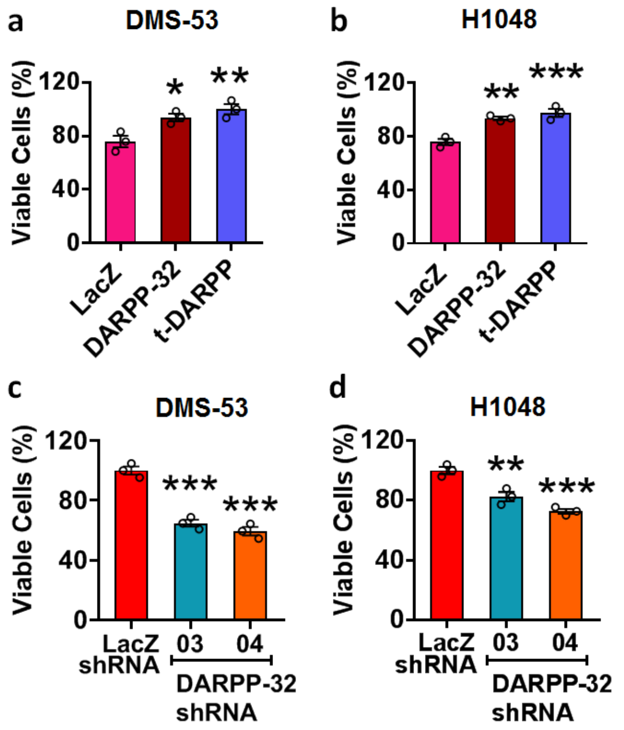

Supplementary Figure 3: Overexpression of DARPP-32 isoforms increases SCLC cell viability. **a-b** Human DMS-53 (a) and H1048 (b) cells transduced with control-, DARPP-32- or t-DARPP-overexpressing clones were plated into 96-well cell culture plates for 72h. MTS-1 reagents were used to quantify viable cells by measuring absorbance at 490 nm. **c-d** Colorimeter-based cell survival assays using MTS reagents were performed in DMS-53 (c) and H1048 (d) cells transduced with control shRNA or DARPP-32 shRNAs. Each open circle on a bar graph represents an independent experiment. Experiments were repeated three times. Error bars indicate SEM. *P<0.05, **P<0.01, and ***P<0.001, one-way ANOVA followed by Dunnett's test for multiple comparison.

Supplementary Figure 4

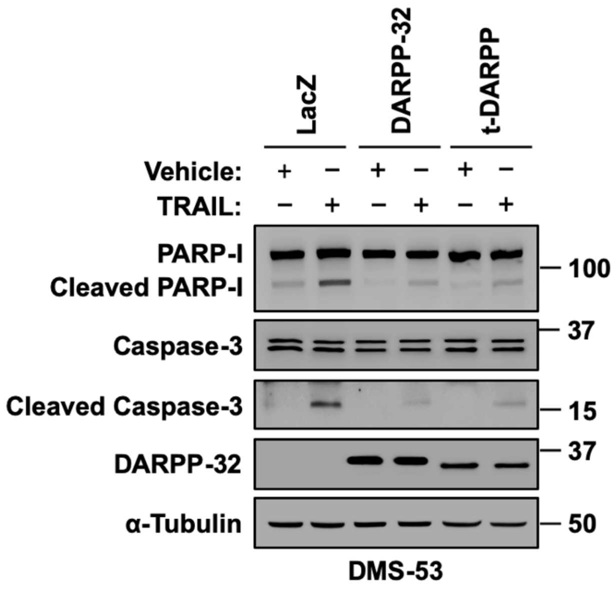

Supplementary Figure 4: Overexpression of DARPP-32 isoforms protects cells from TRAIL-induced apoptosis. Human SCLC DMS-53 cells transduced with retrovirus encoding LacZ (control), DARPP-32, and t-DARPP cDNA plasmids were treated with either vehicle (water) or TRAIL (250 nM) for 24h. Cells were lysed and immunoblotted with antibodies to detect cleaved and uncleaved PARP-I, cleaved and uncleaved (i.e., pro-) caspase-3, DARPP-32 and α-tubulin (loading control). Experiments were performed three times. Results of one representative experiment were shown.

Supplementary Figure 5

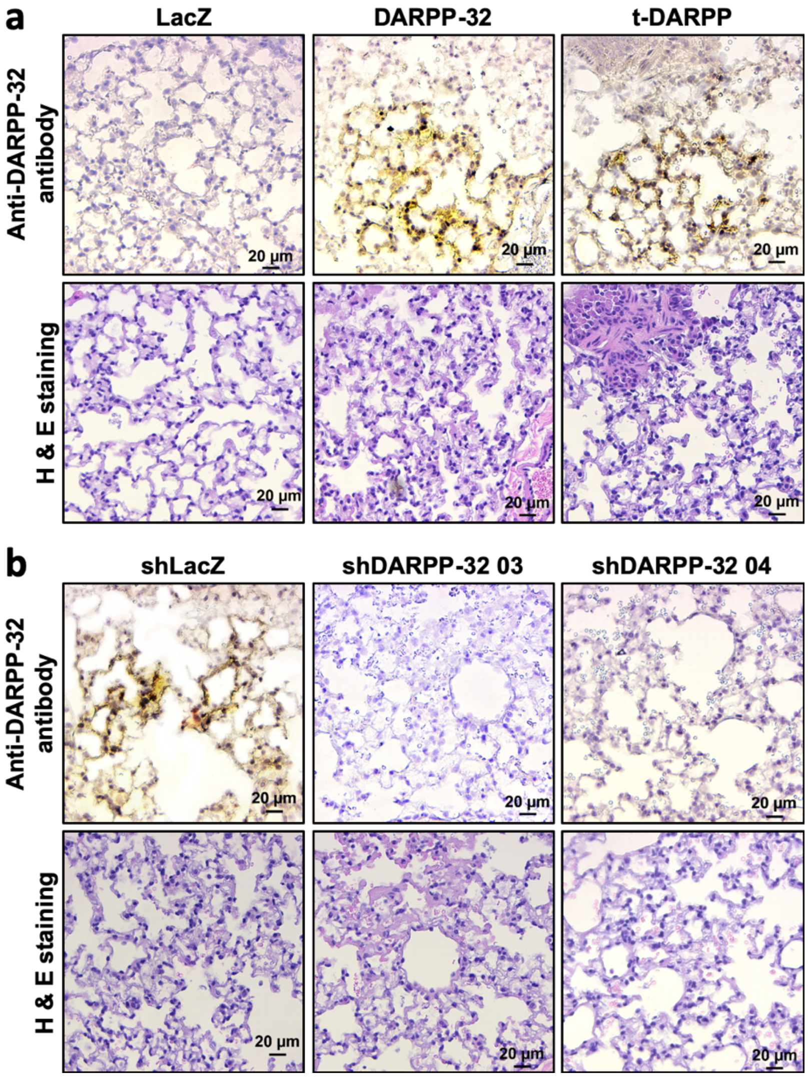

Supplementary Figure 5: Verification of DARPP-32 and t-DARPP expression in explanted tumor xenografts. a Human SCLC DMS-53 cells stably overexpress LacZ, DARPP-32, or t-DARPP proteins were orthotopically injected into SCID mice. The tumors were grown over the time in vivo and animals were sacrificed at the end of the experiment. Lung tissues harvested from mice were fixed in formaldehyde, embedded in paraffin, serially cross-sectioned, and subjected to immunohistochemistry (IHC) with an antibody that detects both DARPP-32 and t-DARPP protein. Nuclei and cytosol were stained with hematoxylin and eosin (H & E), respectively. **b** DARPP-32-ablated DMS-53 cells were orthotopically injected into SCID mice and explanted tumors were subjected to IHC using anti-DARPP-32 antibody. H & E staining were performed to visualize tissue morphology. Scale bar indicates 20 μm. IHC experiments were repeated in three mice lung tissues from each group. Results represent one single experiment.

Supplementary Figure 6

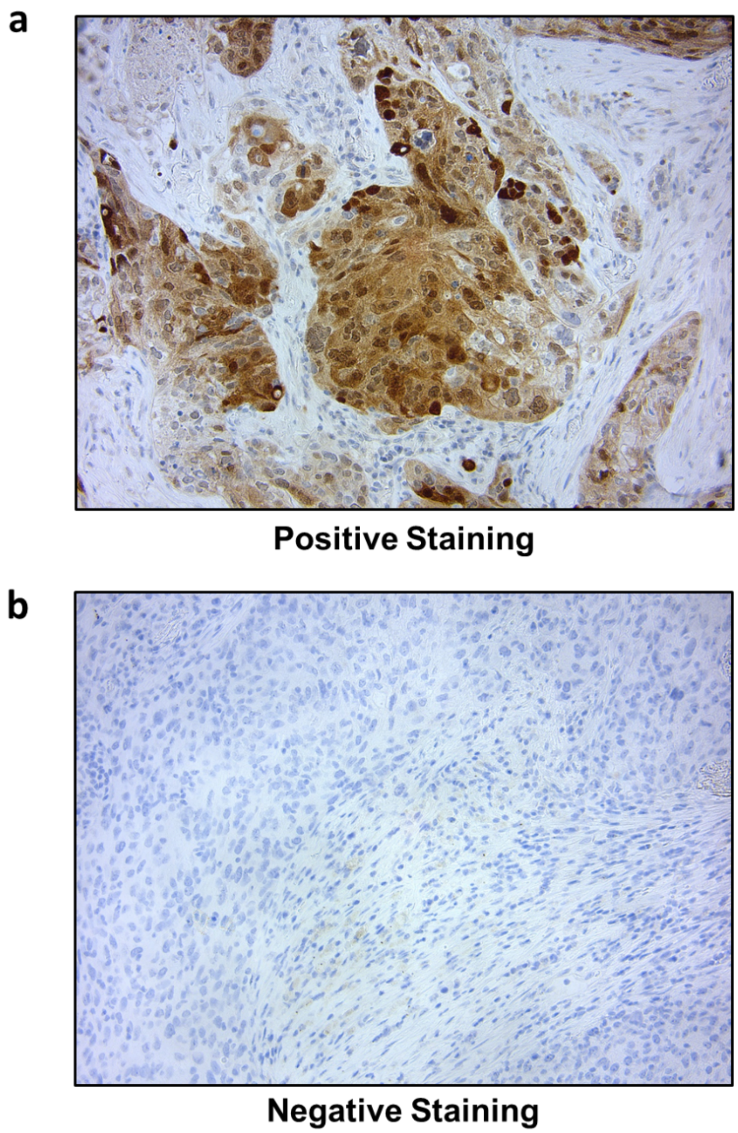

Supplementary Figure 6: Antibody specificity controls for DARPP-32 antibody used in the IHC studies. a. IHC was performed using a C-terminal DARPP-32 antibody that detects both DARPP-32 and t-DARPP (i.e. positive staining) and **b.** no primary antibody (i.e. negative straining). Serial sections of the same reference human SCLC tissues were used for antibody specificity controls shown in this figure.

Supplementary Figure 7

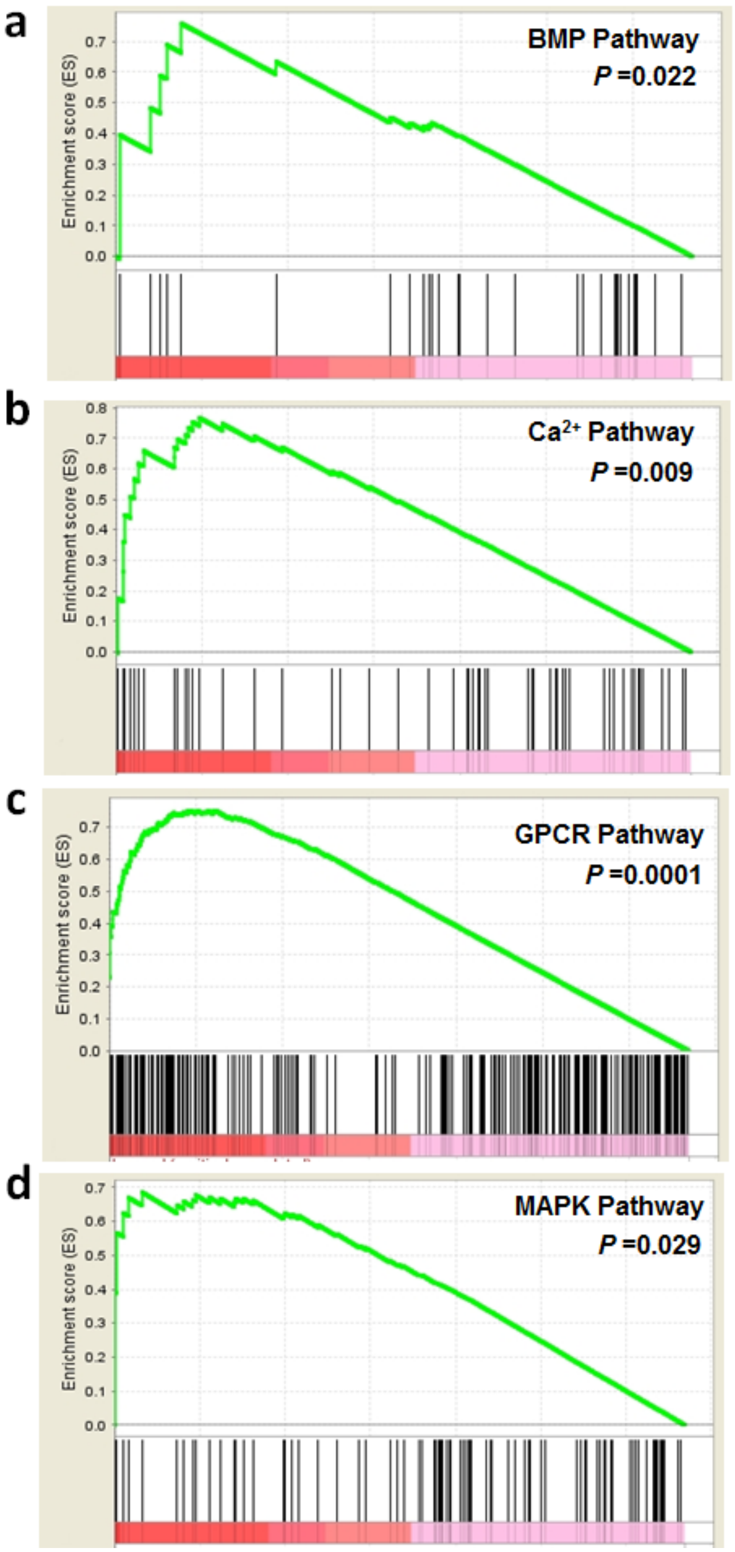

Supplementary Figure 7: Gene set enrichment analysis (GSEA). a Bone morphogenetic protein (BMP) pathway, **b** Calcium signaling (Ca2+) pathway, **c** G protein coupled receptor (GPCR) pathway and **d** mitogen-activated protein kinase (MAPK) pathway were shown to be enriched in a subset of SCLC patients with elevated t-DARPP levels in tumor tissue by performing GSEA.

Supplementary Figure 8

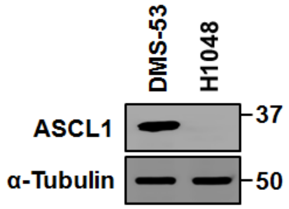

Supplementary Figure 8: ASCL1 is upregulated in DMS-53 cells. Two human SCLC cell lines, namely DMS-53 and H1048, were lysed and immunoblotted with antibodies against ASCL1 and α-tubulin (loading control).

Supplementary Table 1: List of primers used in this study

Primers used in site-directed mutagenesis

Name	Forward Primer (5'-3')	Reverse Primer (5'-3')
ASCL1-SDM01	GGAGGCTTCTCTGCCCTCATCTCCCTTCTACTGATCCCCAGCCTGGGATC	GATCCCAGGCTGGGGATCAGTAGAAGGGAGATGAGGGCAGAGAAGCCTCC
ASCL1-SDM02	ATAAGCCCTCCTGCTCAAATGGGGCTACTGATCCATGTTCCCTGAGGCCTTTTC	GAAAAGGCCTCAGGGAACATGGATCAGTAGCCCCATTTGAGCAGGAGGGCTTAT
ASCL1-SDM03	CCTACACCCCTGGGCCAACAACTTATCATCACTGATTTTCCCAACAAGTATATAAGTCAACAAACA	TGTTTGTTGACTTATATACTTGTTGGGAAAATCAGTGATGATAAGTTGTTGGCCCAGGGGTGTAGG

Primers used in sanger sequencing

Name	Forward Primer (5'-3')	Reverse Primer (5'-3')
DARPP-32 promoter	CCTCTGTCAGCCAGTCTTCC	TCTTCTGGAATTGGGGTCAG

Supplementary Table 2: Immunostaining scores of patient-derived human SCLC tissue samples

SCLC Patient No.	Figure No.	DARPP-32+ cells (%)	Intensity	IHC score
1	4G	95	3	285
2	4H	30	3	90
3	4I	10	3	30
4	4J	80	3	240
5	4K	>90	3	>270
6	4L	90	3	270

Supplementary Table 3: List of upregulated genes with fold change relative to normal

Gene Name	Fold change relative to normal
OR51E1	711.6
AMER3	283.8
FEZF2	261.8
FGF5	250.1
AC005307.3	229.3
ELAVL2	229.1
CALCA	229.0
AC005307.1	182.8
GABRG3	170.6
DLX6-AS1	163.6
C18orf42	145.0
GFY	137.4
RP11-51M18.1	128.2
SIM1	125.3
RP11-669N7.2	122.6
KLK12	122.1
EMX2OS	121.7
STMN2	118.7
MARCH11	117.4
CACNG5	114.3
MIR519A2	111.7
RP11-277I20.3	109.5
BRS3	108.0
RP11-139K4.2	104.6
RP11-665C14.2	102.5
TBXT	101.6
GAD2	94.4
RP11-438B23.2	91.2
ATP2B3	88.5
LCN15	88.2
IL1RAPL2	81.8
CNTNAP2	80.2
TUNAR	79.9
KCNH5	79.6
LRRC53	79.0
SYT4	75.5
CGA	71.9
RP11-1038A11.2	70.5
GABRG2	69.3
GRIK3	69.1
TPH1	67.9
CTNNA2	67.0
CASC18	66.5
EMX1	66.0
CPA5	65.5
RP11-52L5.6	65.2
FUT9	64.8
PDX1	63.7
CER1	63.6

Gene Name	Fold change relative to normal
RP11-64C1.1	63.2
LRTM2	62.3
EPHA6	61.6
MAGEA9	61.4
GABRQ	61.1
HMGB3P18	59.5
CNTN2	58.6
LINC01249	57.6
MAGEC1	57.4
NKX2-2	57.4
SOX1	56.8
KCNH6	56.2
RP11-474D1.3	56.0
DLX5	55.7
ATOH7	55.0
HAPLN1	54.6
SLC24A2	54.6
RP5-984P4.6	54.4
SYT6	54.3
SLITRK1	53.4
RET	52.8
POU3F2	52.3
CTD-2140G10.2	52.2
BARHL2	51.7
LINC00626	51.3
LINCR-0002	51.0
PEX5L	50.8
FGF12-AS2	50.3
KCNB2	50.2
RNF186	49.8
SLC18A1	49.5
PCSK1	49.4
NELL1	49.4
ADCY2	49.3
RP11-317N12.1	48.7
RP11-1C8.4	48.6
TEX101	48.6
AC012506.2	48.6
TACR3	48.2
DLX6	48.1
ZMAT4	48.1
CPLX2	47.4
VGF	47.3
GLYATL3	47.1
ASCL1	47.0
FLJ36000	45.8
STXBP5L	45.7
RP1-212P9.2	45.6
GRP	45.6
NXPH1	45.6
FOXD3	45.5

Gene Name	Fold change relative to normal
CACNA1E	45.0
POU4F1	44.7
RAX	44.6
RP11-48F14.2	44.6
TEX15	44.2
FOXD3-AS1	44.2
IGFBPL1	44.0
KCNC2	43.9
RP11-277I20.2	43.6
ZIC2	43.5
CALCB	43.4
HOXC13	43.4
CHGA	43.3
PNMA6D	43.2
NCAN	42.9
NEUROG3	42.8
SORCS3	42.7
KIRREL3	42.6
SELV	42.5
PTH2	42.4
NAT16	42.2
ZIC5	41.7
SYT14	41.7
ONECUT2	41.5
KRT83	41.0
RP11-114G22.1	41.0
CTD-2566J3.1	40.5
AC079466.1	40.5
MARCH4	40.2
CTD-2089N3.1	40.0
RP11-300M24.1	39.9
RP11-328N19.1	39.8
PTF1A	39.8
RP11-501O2.5	39.8
LMO1	39.7
TRH	39.5
SPAG11B	38.9
ACBD7	38.4
CADPS	38.4
RP11-81H3.2	38.0
MYO3A	38.0
OTOG	37.9
FOXL2	37.8
GNG8	37.8
TBX20	37.8
TMEM145	37.8
ZBTB8B	37.6
RTBDN	37.5
RP11-675F6.3	37.5
RP11-13A1.1	37.3
TPSP2	37.1

Gene Name	Fold change relative to normal
AL109763.2	36.8
DLK1	36.7
ACTL6B	36.6
UMODL1	36.6
SP8	36.6
SCN3A	36.6
FAM132B	36.0
BEST3	35.8
OPRD1	35.7
CST1	35.6
NLRP11	35.5
KCNK2	35.3
OLFM3	35.2
LINC00404	35.2
ATP4B	35.1
CHRNB2	34.9
NEUROD1	34.9
DCX	34.8
GPR6	34.5
PRKCG	34.5
LINC00461	34.5
LL22NC03-121E8.	34.5
RP11-1C8.7	34.2
SHOX2	34.0
ST8SIA5	34.0
DLX1	34.0
CASC20	34.0
RP11-153K16.1	33.7
DLX2	33.5
SVOP	33.5
CTD-2147F2.1	33.4
SOWAHA	33.4
PAQR9	33.3
NPC1L1	33.3
RP11-13K12.2	33.2
LINC00669	33.2
ISL1	33.1
ATCAY	33.1
SEZ6L	33.0
DCAF12L2	33.0
RP11-742D12.2	32.9
PHYHIPL	32.9
BRINP2	32.8
RP11-1103G16.1	32.8
CLVS2	32.7
AC012123.1	32.6
RP11-316M20.1	32.6
KCNC1	32.5
DLL3	32.3
SCG3	32.3
HTR1E	32.2

Gene Name	Fold change relative to normal
TMEM74	32.0
VIL1	32.0
OPCML	32.0
GNG4	32.0
LINGO2	31.9
TCERG1L	31.7
TMEM132B	31.7
BARX1-AS1	31.7
PCDHGC4	31.7
PCDH8	31.5
GRIN2C	31.4
RP11-429B14.4	31.4
RP11-30L8.1	31.4
KCNH7	31.3
HIST1H3J	31.2
GJD2	31.1
PTPRN	31.1
AF131216.5	30.9
AC113607.2	30.9
RAB3B	30.9
CEND1	30.8
PCDHA5	30.7
TRIM67	30.6
FAM163B	30.6
CHRNB4	30.6
KLHL1	30.6
RALYL	30.5
WNT16	30.5
MUC13	30.5
NRSN1	30.3
LINC00592	30.3
GNG13	30.3
FOXI3	30.1
AC012354.6	30.1
SLC25A48	30.1
RP13-786C16.1	30.0
RP11-675F6.4	30.0
GABBR2	30.0
HOXA13	30.0
RIMS2	29.8
CYP4F23P	29.7
BSND	29.7
DNER	29.7
FRRS1L	29.6
SSTR2	29.5
SYT13	29.5
DRD2	29.5
LINC00403	29.4
B3GALT5	29.4
ATRNL1	29.4
CNPY1	29.2

Gene Name	Fold change relative to normal
DAPL1	29.2
AC009410.1	29.2
INA	29.1
NDST4	29.0
SLC7A14	28.9
C8orf12	28.8
CAMK2B	28.8
CTD-2314G24.2	28.7
SLC6A15	28.7
ZDHHC22	28.7
C21orf88	28.6
RP11-85O21.2	28.6
LYPD1	28.5
SLC6A17	28.3
C1QL4	28.3
RP11-1070N10.5	28.3
CDK5R2	28.2
RP13-714J12.1	28.1
COX6B2	28.1
RP11-78L16.1	28.1
TRDN	28.0
LMX1B	27.9
HOXB8	27.8
XKR7	27.7
HOXB9	27.6
CTD-2203A3.1	27.6
RP11-398J5.1	27.4
RP11-87G24.6	27.4
GCK	27.3
MUC19	27.3
RP11-138J23.1	27.2
RP4-738P15.1	27.1
CTB-49A3.4	27.1
RP11-143K11.1	27.1
ONECUT1	26.9
KIF4B	26.9
SIX3-AS1	26.9
RP11-423G4.7	26.9
NGB	26.8
SLC35D3	26.8
KLK2	26.7
ELOVL2	26.6
ENTPD8	26.6
TRPM5	26.5
FOXN4	26.5
ATP4A	26.5
ELAVL4	26.4
CALML3-AS1	26.3
TAAR1	26.3
SHANK2-AS3	26.3
IGFL4	26.3

Gene Name	Fold change relative to normal
RGS7	26.2
AF064860.7	26.2
CTD-2021H9.3	26.1
CNTNAP5	26.1
RP11-89K21.1	26.1
RP11-844P9.2	26.0
CRYBA2	26.0
RP11-445F12.1	25.9
CABP7	25.9
CAMKV	25.8
RUNDC3A	25.7
HMGB3P13	25.7
DPYSL5	25.7
KCNK10	25.7
SEZ6	25.6
LINC00491	25.6
RP11-389G6.3	25.4
CACNA1A	25.3
GHRH	25.3
TUSC8	25.2
BEX1	25.2
RP11-531A24.3	25.1
AC069277.2	25.1
RP11-60A24.3	25.1
HIST1H2BH	25.1
TMSB15A	25.1
INSM1	25.1
KIF4A	25.0
TMSB15A	24.9
CTD-2292M14.1	24.9
RP11-478J18.2	24.9
COL22A1	24.9
C11orf87	24.9
FLRT1	24.8
MAGEC2	24.8
ZAN	24.7
KCNK9	24.7
KIF1A	24.6
CALB1	24.6
LINC00355	24.6
EPHA7	24.5
SCRT2	24.5
RP3-410B11.1	24.5
MTUS2-AS1	24.4
RP11-19O2.2	24.4
SMOC1	24.4
AC013733.3	24.4
C19orf45	24.3
ST18	24.3
SUCNR1	24.3
EPHB1	24.3

Gene Name	Fold change relative to normal
E2F7	24.2
HOXC-AS2	24.2
GAD1	24.2
NPPB	24.2
OXT	24.1
NKX2-5	24.1
FEZF1-AS1	24.0
SYT5	23.9
DGKB	23.9
NRXN1	23.8
CALY	23.8
ZYG11A	23.8
TRPM8	23.7
FSTL5	23.6
ASXL3	23.6
MSI1	23.6
BTBD17	23.5
KIF5C	23.5
KCNJ6	23.3
CLSTN2	23.3
AGMO	23.3
FOXE1	23.2
RPRML	23.2
MIR770	23.2
SYT1	23.2
NKAIN2	23.2
BEST2	23.1
GABRR1	23.1
PRLHR	23.1
RP11-8L2.1	23.1
SCGB1D2	23.1
MMP12	23.1
CTD-2006C1.6	23.1
PCP4	23.1
LINC01096	23.1
CRABP1	23.0
SOX2-OT	23.0
CTB-107G13.1	23.0
TAF7L	23.0
DMBX1	22.9
AC144833.1	22.9
PENK	22.8
MELK	22.8
RP13-766D20.4	22.8
SLC38A3	22.7
ELOVL4	22.6
RP11-734K21.5	22.5
LA16c-329F2.1	22.5
PAX1	22.5
RGS17	22.5
LINC00237	22.3

Gene Name	Fold change relative to normal
RP5-1063M23.2	22.3
AOC1	22.3
DAND5	22.3
HEPACAM2	22.3
TRIM9	22.3
NPPA	22.2
NCAM1	22.2
SPOCK3	22.2
RP11-424C20.2	22.2
PASD1	22.2
DEPDC1	22.2
MIR7-3HG	22.1
BRSK2	22.1
ATP1A3	22.0
LRFN2	22.0
GLYATL1	21.9
GLDC	21.9
TAGLN3	21.9
ZNF488	21.9
DPYSL4	21.9
DBX1	21.9
SHISA7	21.8
SCN8A	21.7
RIPPLY2	21.7
LINC01143	21.7
CHGB	21.6
HOXD10	21.6
RP11-57G22.1	21.5
BIRC5	21.5
FAM64A	21.5
RP11-120K24.5	21.5
ZNF695	21.5
KCNMB2	21.4
UNC79	21.4
UCHL1	21.4
FOXB1	21.4
TCEAL5	21.3
HOXD11	21.3
CH17-38B12.2	21.3
BBOX1-AS1	21.3
RP11-713C5.1	21.3
RP11-3B12.5	21.2
INSRR	21.2
NUF2	21.2
LRRC14B	21.1
GRM4	21.1
LIN28A	21.1
UNC80	21.1
LINC00381	21.1
ELAVL3	21.1
RP11-629G13.1	21.0

Gene Name	Fold change relative to normal
AC010975.1	21.0
OGDHL	21.0
ASPM	21.0
LINC00466	20.9
ENTPD2	20.9
RP11-227D2.3	20.9
SIX2	20.9
RP11-353N14.1	20.9
POU6F2-AS2	20.8
AP000251.3	20.8
ALX4	20.8
RAET1K	20.8
ELOVL2-AS1	20.8
RP11-527D7.1	20.7
SPSB4	20.7
LINC00928	20.7
LINC00648	20.7
KCNA1	20.6
CDH2	20.6
CTD-2320G14.2	20.6
HIST1H2AL	20.6
FAM111B	20.5
FEV	20.4
RP11-357K6.1	20.4
CLSPN	20.4
DPP10	20.4
AMH	20.4
RPS11P6	20.3
C12orf56	20.2
KLK3	20.2
RP11-438D14.2	20.2
C1orf95	20.1
INHBE	20.0
SNAI1P1	20.0
GPR144	20.0
DLGAP5	19.9
RP11-96H17.1	19.8
DRAXIN	19.8
SLC12A1	19.8
RP11-551L14.1	19.8
L1CAM	19.8
LHX2	19.8
C6orf183	19.7
ST8SIA3	19.7
OTOP3	19.7
PPM1E	19.7
RP5-984P4.4	19.7
UNC5A	19.7
RP11-1038A11.3	19.6
CNTD2	19.6
SOX2	19.6

Gene Name	Fold change relative to normal
RP11-883G14.1	19.5
GRM2	19.5
RPL32P33	19.4
RP5-823G15.5	19.4
RIMBP2	19.4
SPOCK1	19.4
RP11-728G15.1	19.4
NPTX2	19.4
RP11-117L6.1	19.3
RP11-74E22.8	19.3
DGCR9	19.3
RP11-385J1.2	19.3
CTD-2377D24.4	19.3
RP11-494H4.3	19.3
MNX1-AS1	19.2
RP11-397A16.1	19.2
SOX11	19.2
PROX1-AS1	19.2
MEX3A	19.2
C1QL1	19.1
RP11-108E14.1	19.1
RGS4	19.0
AP3B2	19.0
SSTR5-AS1	19.0
RP11-15G16.1	19.0
SYT10	19.0
RCOR2	19.0
TUBB2B	18.9
NMNAT2	18.9
RP11-74E22.4	18.9
HP09025	18.9
SLC5A12	18.8
TPH2	18.8
PAH	18.8
CCDC177	18.8
SCGN	18.7
CLGN	18.7
CDKN2A	18.7
RAD54L	18.7
PAX4	18.7
BAI2	18.7
AC108676.1	18.7
SV2A	18.6
AC007128.1	18.6
TOP2A	18.6
HIST1H1B	18.6
PROX1	18.6
GPR139	18.6
ALG1L3P	18.5
RP11-561B11.6	18.5
ADCYAP1	18.5

**Gene Name**	**Fold change relative to normal**
AC116614.1	18.5
FAM155B	18.4
HNF4G	18.4
HERC2P10	18.4
RP11-142C4.4	18.4
RP11-431M3.1	18.3
LL09NC01-254D1	18.3
CELF4	18.3
SALL1	18.3
NXPH4	18.3
DLGAP3	18.3
GDAP1L1	18.3
ASNSP3	18.3
HOXD12	18.2
INSM2	18.2
HIST1H2BF	18.2
GPRIN1	18.2
FAM19A1	18.2
FSD1	18.1
TERT	18.1
RP5-1119A7.17	18.1
MEIOB	18.0
PBK	18.0
PHF21B	18.0
LINC01346	18.0
FRMD5	18.0
RP11-540E16.2	18.0
HES6	18.0
KRT9	17.9
RP11-796E10.1	17.9
RP11-55K22.2	17.9
NEK2	17.9
HNF1A	17.9
DDX25	17.9
MIR490	17.8
RP11-100K18.1	17.7
RP11-378I6.1	17.7
SNX18P7	17.7
LA16c-321D4.2	17.7
LINC01250	17.7
VWC2L	17.7
RP11-1L9.1	17.6
MAGEA6	17.6
GLYATL1P2	17.6
KRTAP4-1	17.5
TMEM178B	17.5
SCN2A	17.5
RP11-365D9.1	17.5
RP11-576D8.4	17.5
PROKR1	17.5
TMEFF2	17.5

Gene Name	Fold change relative to normal
PCDHGA1	17.4
UNC13A	17.4
CTD-2307P3.1	17.4
NR0B1	17.4
LINC01152	17.4
KIF14	17.4
UBE2SP2	17.4
CELF3	17.3
RP11-672L10.2	17.3
KIF18B	17.3
CA9	17.2
DPF1	17.2
RP11-268G13.1	17.2
PRSS29P	17.2
TSHR	17.2
FGF12-AS3	17.2
SRRM4	17.2
KCNJ3	17.2
MCM10	17.1
CTD-2034I21.2	17.1
RP11-115D19.1	17.1
NRCAM	17.1
SHD	17.0
CTD-2089N3.2	17.0
CD24P4	17.0
RFX4	17.0
SULT4A1	17.0
KIF5A	16.9
LINC00634	16.9
GPX2	16.9
C9orf53	16.9
RP11-379L18.1	16.9
ESPL1	16.9
CCNE2	16.8
GNGT1	16.8
TTK	16.7
MIR335	16.7
RP11-552E20.4	16.7
SATL1	16.7
CA6	16.6
E2F8	16.6
BHLHE23	16.6
SIX3	16.6
PCDHB17	16.6
OPTC	16.6
MTNR1B	16.6
RP11-616M22.7	16.6
A1CF	16.6
EYA2	16.6
ASF1B	16.6
DEPDC1B	16.6

**Gene Name**	**Fold change relative to normal**
HOXD4	16.6
FBLL1	16.5
ILDR2	16.5
STIL	16.5
CTD-3006G17.2	16.5
C6orf195	16.5
ADAMTS18	16.5
GPR158	16.4
AC105402.4	16.4
NOL4	16.4
HOXA10	16.4
CTD-2527I21.15	16.4
BARX1	16.4
CTSV	16.4
NKX2-4	16.3
RP11-776H12.1	16.3
VENTXP5	16.3
RP11-771K4.1	16.3
AVPR1B	16.3
RP11-474D1.4	16.2
CCKBR	16.2
RP11-353N14.2	16.2
LINC01079	16.2
U82695.5	16.1
CDCA8	16.1
ASTN1	16.0
RP11-65D17.1	16.0
RP11-685A21.1	16.0
ERVMER61-1	16.0
WNK3	16.0
PTCHD2	16.0
RP1-76B20.11	15.9
RELN	15.9
ADARB2-AS1	15.9
DNAJC22	15.9
CENPI	15.9
RP11-108K3.2	15.9
NKAIN1	15.9
ADD2	15.8
MAST1	15.8
CDC25C	15.8
PCDHGB1	15.8
KCNH8	15.8
SLC6A5	15.8
SLCO6A1	15.8
RNF144A-AS1	15.8
RP11-25H12.1	15.8
CTD-2234N14.2	15.8
TPX2	15.8
CPNE4	15.8
SLITRK6	15.7

**Gene Name**	**Fold change relative to normal**
RP4-676L2.1	15.7
RP11-656G20.1	15.7
EXO1	15.7
LINC00898	15.7
CHST8	15.7
RIPPLY3	15.7
SYN1	15.6
UHRF1	15.6
TMEM151B	15.6
NGEF	15.6
RP5-1028L10.2	15.6
ADCY1	15.6
RP11-567M16.1	15.6
RPS16P9	15.6
LINC00958	15.5
MYT1	15.5
KRT17P1	15.5
OR51E2	15.5
RP11-538I12.3	15.5
SNAP25	15.5
RP11-605F22.2	15.5
HIST1H3G	15.5
RP11-103J17.2	15.5
KIF2C	15.4
TUBB3	15.4
RHBDL3	15.4
HOXB-AS3	15.4
FGF12	15.4
RP11-284F21.10	15.4
PCDH19	15.4
CENPF	15.4
DDC	15.4
WNK2	15.4
TMPRSS4-AS1	15.3
MGAT5B	15.3
APLP1	15.3
CFHR3	15.3
RP11-450H6.3	15.3
RP11-375B1.3	15.3
CKAP2L	15.3
FMN2	15.3
CTC-338M12.9	15.3
EPHA8	15.2
RP11-794G24.1	15.2
KIF19	15.2
EEF1A2	15.2
RP5-827C21.2	15.1
CCDC129	15.1
SOHLH2	15.1
LINC01413	15.1
GINS2	15.1

Gene Name	Fold change relative to normal
RP11-402J6.1	15.0
AIRE	15.0
BRIP1	15.0
UBE2T	15.0
IGSF1	15.0
FOXE3	15.0
FAM72B	14.9
AC005624.2	14.9
FOXD1	14.9
AMPH	14.9
RP11-657O9.1	14.9
BHMT	14.9
LHX1	14.8
RP11-57A19.2	14.8
PCDHB1	14.8
TROAP	14.8
CAMK2N2	14.8
RBFOX3	14.8
KIRREL3-AS3	14.8
TRPA1	14.7
TOMM20P3	14.7
IQGAP3	14.7
MROH3P	14.7
COCH	14.7
UBE2QL1	14.7
AC006946.15	14.7
TENM3	14.7
MAP1B	14.7
DPP10-AS1	14.6
ACTL8	14.6
MUC6	14.6
SH3GL2	14.6
ANK1	14.6
MMP26	14.6
KCNJ4	14.6
GRM8	14.5
VSX1	14.5
GINS1	14.5
VWA5B2	14.5
RP11-680H20.2	14.5
THEG	14.5
GPIHBP1	14.5
NDC80	14.4
BSX	14.4
HERC2P5	14.4
ORC6	14.4
LHX3	14.4
RP11-672L10.1	14.4
AC067961.1	14.4
B4GALNT4	14.4
MNX1	14.4

Gene Name	Fold change relative to normal
FOXM1	14.3
KLHL14	14.3
CHRM4	14.3
NR5A1	14.3
CACNA1B	14.3
PPFIA4	14.3
PFN2	14.3
RP11-108M12.3	14.3
RP11-758M4.1	14.3
PCDH8P1	14.3
ESPNP	14.3
KCNH3	14.2
VWA5B1	14.2
SOX21	14.2
CDKN2C	14.2
LINC01116	14.2
ECT2	14.2
SCN5A	14.1
U91319.1	14.1
NUSAP1	14.1
HIST1H3F	14.1
CTA-254O6.1	14.1
LINC00470	14.1
MAGED4	14.0
HJURP	14.0
FAM72C	14.0
LHFPL5	14.0
FBXO43	14.0
TFF3	14.0
PKIB	14.0
MKI67	14.0
KIAA0087	13.9
TUBB2BP1	13.9
GP2	13.9
RP11-161I6.2	13.9
OTP	13.9
POU4F2	13.9
USH1C	13.9
GRM5	13.8
RP11-109M17.2	13.8
TRPM2-AS	13.8
RP11-666A20.3	13.8
RP1-40E16.9	13.8
HOXA10-AS	13.8
FEZF1	13.8
CTD-2568A17.1	13.8
DGCR5	13.8
RP11-279F6.1	13.8
GTSE1	13.8
ZNF711	13.8
CELSR3	13.8

Gene Name	Fold change relative to normal
SNCB	13.8
CCDC169	13.8
CTD-2377D24.6	13.7
DNAJC12	13.7
KRT8P30	13.7
LINC01208	13.7
CHD5	13.7
MS4A8	13.7
C12orf42	13.7
HIST1H4B	13.7
FAM57B	13.6
RP11-738B7.1	13.6
TLX1	13.6
CDC20	13.6
SP9	13.6
MIR196A1	13.5
SLC30A2	13.5
CTD-2587H24.10	13.5
CBX2	13.5
SRCIN1	13.5
BUB1B	13.4
RP11-284F21.9	13.4
RNF183	13.4
CDIPT-AS1	13.4
GSX1	13.4
AC018641.7	13.4
ANO4	13.4
RP11-496N12.6	13.3
GNAO1	13.3
CUX2	13.3
HIST1H4A	13.3
LINC01018	13.3
NCAPG	13.3
SLC8A2	13.3
HIST1H3C	13.3
B4GALNT1	13.3
RP4-694A7.2	13.3
SI	13.3
TYMSOS	13.2
RASAL1	13.2
SCML2	13.2
USP41	13.2
LINC01234	13.2
FZD9	13.2
ST8SIA6-AS1	13.2
NMU	13.2
CRMP1	13.2
SEC11C	13.2
XKR5	13.2
MCM2	13.2
UGT8	13.2

**Gene Name**	**Fold change relative to normal**
ORC1	13.2
SRRM3	13.1
MAPT	13.1
CASC5	13.1
AP001631.9	13.1
KC6	13.1
ALX3	13.1
HOXB5	13.1
FAM131C	13.1
KLHDC8A	13.1
DYNC1I1	13.1
CKMT1B	13.1
MMP16	13.1
TFF1	13.1
RP11-286B14.1	13.0
EFTUD1P1	13.0
ESCO2	13.0
DUSP26	13.0
GAS2	13.0
AC003102.3	12.9
AC008088.4	12.9
CCL25	12.9
CNTFR	12.9
AC009014.3	12.9
PCDHGA8	12.8
CTD-2318O12.1	12.8
RAB3C	12.8
RP11-480I12.4	12.8
CHRNA5	12.8
DIRAS2	12.8
TMEM8C	12.8
PCSK2	12.8
RP13-614K11.2	12.8
CCDC34	12.8
EMILIN3	12.8
CTD-2256P15.1	12.8
LINC01122	12.7
CDCA5	12.7
RP11-513G11.4	12.7
LYPD6	12.7
MAD2L1	12.7
RP11-209K10.2	12.7
SLC30A10	12.7
RP11-698N11.2	12.7
CACNG2	12.6
RP11-449P1.1	12.6
TMEM63C	12.6
PNMA6C	12.6
RRM2	12.6
SPC24	12.6
GREB1L	12.6

Gene Name	Fold change relative to normal
LRFN5	12.6
GS1-24F4.2	12.6
CDC45	12.6
WDR62	12.6
ADAM22	12.5
KIAA1549L	12.5
POLQ	12.5
PRSS3P2	12.5
CSAG1	12.5
RP11-373E16.3	12.5
AK5	12.5
ATP2B2	12.5
HIST1H2AE	12.5
HDAC1P2	12.5
NCAPH	12.5
CDC6	12.5
TAL2	12.4
WASF1	12.4
CECR6	12.4
NAT8L	12.4
DRP2	12.4
BVES-AS1	12.4
IGF2BP3	12.4
TP73	12.4
RPS27P25	12.4
CCNB2	12.4
UBE2SP1	12.4
KIAA2022	12.4
SCG5	12.3
NPTX1	12.3
PEG10	12.3
IGFL1P2	12.3
RP11-546J1.1	12.3
KCNT1	12.3
EBF3	12.3
RP11-715J22.6	12.2
KCNC4-AS1	12.2
E2F2	12.2
RP11-541G9.1	12.2
RP11-120K24.4	12.2
MUC2	12.2
ZACN	12.2
NKX3-2	12.2
BSN	12.2
PHGR1	12.2
UBE2C	12.2
SNORD113-3	12.1
PODXL2	12.1
SPATA16	12.1
SLC38A11	12.1
RP11-379L18.3	12.1

Gene Name	Fold change relative to normal
FAR2P3	12.1
RP11-461O7.1	12.1
SCG2	12.1
MPPED1	12.1
PDX1-AS1	12.1
SIX4	12.1
RP11-396O20.2	12.0
RP1-90L14.1	12.0
SLC38A8	12.0
PRC1	12.0
RP11-120J1.1	12.0
RP11-441F2.5	12.0
GRK1	12.0
RP5-827C21.6	12.0
KIF24	12.0
SGOL1	12.0
NETO2	11.9
MAFA	11.9
CNIH2	11.9
RP11-563K23.1	11.9
RP11-159H10.3	11.9
ARHGEF7-IT1	11.9
GPT2	11.9
AC005618.6	11.9
RAB9B	11.9
HIST1H3B	11.9
PATE2	11.9
SEMA5B	11.8
SPAG5	11.8
C20orf203	11.8
MEST	11.8
RP11-410N8.3	11.8
CTC-537E7.2	11.8
ERCC6L	11.8
LRRC10	11.8
FAM171B	11.8
LL0XNC01-16G2.	11.7
SRSF12	11.7
STYK1	11.7
RP11-36N20.1	11.7
C1QL3	11.7
CABYR	11.7
LINC00951	11.7
Metazoa_SRP	11.7
CLPSL2	11.7
FLJ46066	11.7
UBA52P6	11.7
AC009336.24	11.7
NEURL1	11.7
SRPK3	11.6
CHRNA3	11.6

**Gene Name**	**Fold change relative to normal**
TUBA3GP	11.6
CELF5	11.6
CENPA	11.6
RP11-482D24.2	11.6
C16orf59	11.6
FOXO6	11.6
EYA1	11.6
Six3os1_2	11.6
LSAMP-AS1	11.6
DEPDC1-AS1	11.5
OR2B6	11.5
KIF15	11.5
RP11-470P21.2	11.5
SCGB2A1	11.5
NWD2	11.5
ADAM23	11.4
PAK7	11.4
GRIK1	11.4
ZNF724P	11.4
GRIK5	11.4
TRIP13	11.4
RP11-482D24.3	11.4
GPR142	11.4
XRCC2	11.3
MANEAL	11.3
LECT1	11.3
CDC25A	11.3
CTD-2555A7.2	11.3
LRRC9	11.3
PNMA3	11.3
ZNF367	11.3
RP11-321L2.1	11.3
UBE2S	11.3
RP11-480I12.7	11.3
AC010729.1	11.2
RP11-80B9.1	11.2
KIRREL2	11.2
FAM83D	11.2
TICRR	11.2
LINC01397	11.2
KCNF1	11.2
DSCAML1	11.2
RAD51AP1	11.2
CDCA2	11.1
OR7E83P	11.1
PACSIN1	11.1
TRIM45	11.1
MGAT4C	11.1
RP11-78F17.1	11.1
GPR62	11.1
RP11-107C16.2	11.1

**Gene Name**	**Fold change relative to normal**
CD24	11.0
CACNA2D1	11.0
CTD-2231H16.1	11.0
STX1A	11.0
ITIH6	11.0
CDX2	11.0
SYP	11.0
RP4-616B8.6	11.0
BRSK1	11.0
TNNT1	11.0
IL23A	11.0
UMOD	11.0
OR2W6P	11.0
IGSF9	11.0
AC093702.1	10.9
CLPSL1	10.9
MAGED4B	10.9
KLF2P4	10.9
MAP7D2	10.9
KIFC1	10.9
RP11-97O12.2	10.9
RP11-220I1.2	10.9
AC053503.4	10.9
ARHGAP26-AS1	10.9
KIF20A	10.9
AC007326.9	10.9
PCDHA1	10.8
NEIL3	10.8
GABRA3	10.8
PNMAL1	10.8
RP11-439M11.1	10.8
LINC01134	10.8
PEX5L-AS2	10.8
POLE2	10.8
CCDC150	10.7
AURKB	10.7
TLX2	10.7
MESTP3	10.7
RP11-149I2.4	10.7
RAB26	10.7
MFI2	10.7
RP11-416N4.1	10.7
CCDC178	10.7
EVX2	10.7
RP11-485G7.6	10.7
AC096574.4	10.7
SEPT3	10.7
RP11-519M16.1	10.6
HPDL	10.6
ANKRD18B	10.6
RP11-157E16.1	10.6

Gene Name	Fold change relative to normal
RFC4	10.6
TOX3	10.6
MYOD1	10.6
VCX3A	10.6
RP11-715J22.2	10.6
RP5-944M2.3	10.6
KIF25-AS1	10.6
EZH2P1	10.6
RP11-575G13.2	10.6
GPR137C	10.6
SYT2	10.6
PTPRH	10.6
NKX6-1	10.6
NOVA1-AS1	10.6
RP11-346D19.1	10.6
SKA3	10.6
ASTN2	10.5
RP11-364C11.3	10.5
SAPCD2	10.5
LINC00866	10.5
SLCO5A1	10.5
SIX1	10.5
FSIP2	10.5
RP1-310O13.13	10.5
JPH3	10.5
VAT1L	10.5
RP11-187E13.1	10.5
HCN4	10.5
PCDHA4	10.5
GPR19	10.5
LINC00051	10.5
GPR149	10.4
FAM83F	10.4
FOXB2	10.4
RP11-390F4.3	10.4
RP11-713P17.5	10.4
CTD-2532D12.4	10.4
KLK1	10.4
GAP43	10.4
CSMD3	10.4
CDKN3	10.4
PLEKHG4B	10.4
RP11-255E6.6	10.4
LINC00354	10.4
HIST1H2BJ	10.4
RP3-497J21.1	10.4
TIGD3	10.4
DNAJC6	10.4
ALLC	10.4
CTD-2523D13.2	10.4
TBX10	10.4

Gene Name	Fold change relative to normal
FAM222A-AS1	10.4
RP11-485G7.5	10.3
CDK5R1	10.3
MLLT11	10.3
RP11-483C6.1	10.3
HIST1H2BO	10.3
TFAP2A-AS1	10.3
BUB1	10.3
AC234791.1	10.3
SPC25	10.3
PARPBP	10.3
SOX21-AS1	10.3
HIST2H3C	10.3
HMMR	10.3
GRIA2	10.3
OR7E85P	10.3
AGBL4	10.2
OIP5	10.2
DBH	10.2
HELLS	10.2
CACNA1G	10.2
PLK1	10.2
SMC1B	10.2
RP4-604G5.1	10.2
LINGO1	10.2
RAB39B	10.1
HOXD-AS2	10.1
AC090627.1	10.1
RP11-144N1.1	10.1
CLVS1	10.1
RP11-484D2.2	10.1
RP5-1092A11.5	10.1
HMGB1P1	10.1
MYBL2	10.1
RP11-168K9.2	10.1
CTC-480C2.1	10.1
C2orf70	10.0
SBK1	10.0
LINC00269	10.0
AC011322.1	10.0
TDRG1	10.0
RP11-117L5.4	10.0
DDX11-AS1	9.9
ASB15	9.9
REEP2	9.9
RAB39A	9.9
NRG1-IT1	9.9
LINC00884	9.9
LINC00846	9.9
CA5A	9.9
CTC-344H19.4	9.9

Gene Name	Fold change relative to normal
HIST3H2A	9.9
JAKMIP2	9.8
RP4-753M9.1	9.8
YBX2	9.8
RP11-579O24.3	9.8
RP11-254F7.4	9.8
CENPK	9.8
RP11-38M8.1	9.8
LINC00511	9.8
CCNA2	9.8
RP11-280K24.4	9.8
AC064850.4	9.7
C10orf71	9.7
AURKA	9.7
TCF19	9.7
RP11-561N12.6	9.7
U95743.1	9.7
CDH18	9.7
FAM222A	9.7
RASSF6	9.7
SCARNA22	9.7
LINC00303	9.7
LINC01117	9.7
CNKSR3	9.6
MIP	9.6
KLF14	9.6
TCEB3B	9.6
NCAM1-AS1	9.6
WDR72	9.6
HMSD	9.6
AC018685.1	9.6
AC012531.25	9.6
LRP4	9.6
CLDN11	9.6
HIST1H4J	9.6
CTD-2587H24.5	9.6
PAX9	9.6
KIF23	9.6
PPFIA2	9.6
LGALS17A	9.6
RP4-760C5.3	9.5
AC005481.5	9.5
CDCA3	9.5
RP1-159G19.1	9.5
NEB	9.5
RP11-111M22.5	9.5
NPAS3	9.5
AC093063.2	9.5
TTBK1	9.5
RP11-680F20.11	9.5
EZH2	9.5

Gene Name	Fold change relative to normal
CLCN3P1	9.5
ZFR2	9.5
KCNG3	9.5
AMER2	9.5
PDIA2	9.5
FAM171A2	9.5
HGFAC	9.5
LMNB1	9.5
PTTG1	9.4
RP11-170M17.1	9.4
LINC00871	9.4
RP11-1042B17.3	9.4
TRO	9.4
MTUS2	9.4
ACRV1	9.4
AK4P1	9.4
PCDHB8	9.4
AUNIP	9.4
RP11-159H10.1	9.3
ASS1P11	9.3
TCEAL2	9.3
LINC00391	9.3
GPC2	9.3
PAX3	9.3
NOVA1	9.3
ANKS4B	9.3
CPT1C	9.3
CCNB1	9.3
TFAP2A	9.3
MIR4697	9.3
MMD2	9.2
RP11-352G9.1	9.2
CDH4	9.2
RP11-133K1.11	9.2
ZNF667	9.2
RP5-928E24.2	9.2
PCDHA9	9.2
HIST1H2BI	9.2
NPTXR	9.2
RP11-120D5.1	9.2
TSSC1-IT1	9.1
RP11-713P17.3	9.1
CENPU	9.1
RP11-319E16.1	9.1
CCDC110	9.1
RASL10B	9.1
PTPRG-AS1	9.1
LGR4	9.1
NPM1P9	9.1
AC008074.4	9.1
RP11-430H10.1	9.1

Gene Name	Fold change relative to normal
RP11-108L7.15	9.1
TENM4	9.1
AC097713.3	9.1
RP11-423H2.3	9.0
KCNH2	9.0
CNTFR-AS1	9.0
MARK1	9.0
EN2	9.0
KIAA1524	9.0
STRA6	9.0
RP11-524C21.2	9.0
RP11-83M16.6	9.0
RNF157-AS1	8.9
RP11-64K12.10	8.9
RP11-650L12.2	8.9
KIF11	8.9
PSAT1	8.9
RP5-1178H5.2	8.9
FANCI	8.9
ABCC8	8.9
GJB4	8.9
BTBD7P1	8.9
C5orf34	8.9
C17orf104	8.9
CDH24	8.9
SLIT1	8.8
RP11-98G7.1	8.8
PGBD5	8.8
RP11-408N14.1	8.8
RP11-946L16.1	8.8
RP11-88E10.4	8.8
RNU4-1	8.8
MAPK8IP2	8.8
LINC01063	8.8
CHAF1B	8.8
RP11-66N24.7	8.8
CCNE1	8.8
RP1-102D24.5	8.7
KCND2	8.7
RP11-288C17.1	8.7
FOXD2-AS1	8.7
RP11-254F7.1	8.7
SNCAIP	8.7
SYT7	8.7
BRWD1-AS1	8.7
NPPC	8.7
HOXD8	8.7
IZUMO2	8.7
AC034110.1	8.7
BAI1	8.6
STMN1	8.6

Gene Name	Fold change relative to normal
RP11-500B12.1	8.6
HTR3E	8.6
RP11-368L12.1	8.6
LL0XNC01-116E7	8.6
BTBD11	8.6
LRRC16B	8.6
LINC01121	8.6
SERPINH1P1	8.6
AC090945.1	8.6
AC073043.1	8.6
RP11-238F2.1	8.6
VSX2	8.6
RP5-890O15.3	8.6
RP11-54D18.4	8.6
RNU6-438P	8.6
FAM71E2	8.5
RP4-669H2.1	8.5
MYO18B	8.5
LGSN	8.5
AC006262.5	8.5
RP11-831F12.3	8.5
WASIR2	8.5
NTN3	8.5
ADAMTS20	8.5
DGKI	8.5
RP11-536L3.4	8.5
CDHR2	8.5
LINC00471	8.5
RP11-564D11.3	8.5
CASKIN1	8.4
RP11-554D20.2	8.4
RP11-191L9.4	8.4
PRDM12	8.4
MTMR7	8.4
RP11-374A4.1	8.4
GPR173	8.4
RP11-1145L24.1	8.4
SMPD3	8.4
RNASEH2A	8.4
ANLN	8.4
CCDC168	8.4
GRIK2	8.4
RNF157	8.4
RP11-434D9.2	8.4
HCN2	8.4
RP11-324L17.1	8.3
MIAT	8.3
RP11-245D16.4	8.3
C7orf33	8.3
CEP41	8.3
CTD-2147F2.2	8.3

Gene Name	Fold change relative to normal
SYCE2	8.3
TYMS	8.3
LINC01248	8.3
RP11-32D17.4	8.3
NEK2P4	8.3
MCM4	8.2
ARHGAP33	8.2
RP11-384O8.1	8.2
RP11-65M17.3	8.2
MFI2-AS1	8.2
RP11-513G11.2	8.2
SHISA9	8.2
C1orf127	8.2
TAS2R2P	8.2
CHEK1	8.2
COL11A1	8.2
ZNF157	8.2
RP11-1055B8.9	8.2
DMC1	8.2
KCNA2	8.2
CDC7	8.2
KNTC1	8.2
UBL4B	8.2
MYCL	8.2
RP11-100G15.7	8.2
AC104655.3	8.2
AC097468.4	8.2
FAM212B-AS1	8.1
WDR76	8.1
FATE1	8.1
CTD-3099C6.9	8.1
TAC3	8.1
GRIN3A	8.1
C2CD4C	8.1
GNAZ	8.1
FHOD3	8.1
RP11-313C15.1	8.1
NTF3	8.1
HIST1H4C	8.1
XRCC6BP1	8.1
RP11-381E24.1	8.1
AC004540.4	8.1
RFC3	8.0
RP11-173M1.5	8.0
CST4	8.0
RP11-944C7.1	8.0
HIST2H3A	8.0
RP11-93H24.3	8.0
HOXB6	8.0
AP001046.6	8.0
RP11-646E18.4	8.0

Gene Name	Fold change relative to normal
DTL	8.0
ARHGEF39	8.0
RP5-983L19.2	8.0
ENPP7P4	8.0
RP4-565E6.1	7.9
PIF1	7.9
RP11-679B19.1	7.9
CENPE	7.9
CKAP2	7.9
AC084219.4	7.9
MTCL1-AS1	7.9
RNF182	7.9
MIR137HG	7.9
OTOF	7.9
HIST1H2BB	7.9
AHSG	7.9
MARCKSL1	7.9
RECQL4	7.9
FLJ35934	7.9
C17orf53	7.9
EME1	7.9
CKMT1A	7.8
PCDHB9	7.8
HOXB2	7.8
RP1-127B20.4	7.8
RP11-325N19.3	7.8
HIST1H2AD	7.8
AWAT1	7.8
TSACC	7.8
CDK1	7.8
PSRC1	7.8
RP11-521B24.3	7.8
TIMELESS	7.8
HTR1D	7.8
PGAM1P7	7.8
PRR11	7.8
ZWINT	7.8
RP11-496D24.2	7.8
ARG2	7.8
RP11-284F21.7	7.8
SYNGR3	7.8
HMGB3	7.8
GINS4	7.7
CEP55	7.7
FAM178B	7.7
SCAMP5	7.7
LRRIQ4	7.7
RP11-575L7.4	7.7
BVES	7.7
ACYP1	7.7
DMRT3	7.7

Gene Name	Fold change relative to normal
POF1B	7.7
AC008079.9	7.7
WDHD1	7.7
RP11-402G3.3	7.7
CTD-2540M10.1	7.7
RP11-390F4.10	7.7
PAFAH1B3	7.7
MYCNOS	7.7
RP11-680G10.1	7.7
ASPHD1	7.7
DKFZP434H168	7.6
RP3-407E4.4	7.6
RP11-158H5.8	7.6
MED12L	7.6
MRAP2	7.6
TRBV26OR9-2	7.6
MTHFD1P1	7.6
RPRM	7.6
TET1	7.6
GAL	7.6
ESPN	7.6
KRTAP10-6	7.6
RP11-304L19.3	7.6
Metazoa_SRP	7.6
RP5-850O15.3	7.6
CPSF4L	7.6
TMEM132D	7.6
TMSB15B	7.6
METTL11B	7.6
RACGAP1	7.6
SMIM24	7.6
SNRPFP2	7.5
Six3os1_1	7.5
snoU13	7.5
C1orf111	7.5
MYLK2	7.5
BMP8B	7.5
RP11-544I20.2	7.5
CXXC4	7.5
FANCB	7.5
SNORA70	7.5
AF038458.5	7.5
CALHM3	7.5
C2orf48	7.5
RP1-74M1.3	7.5
TMPRSS3	7.5
FLJ22184	7.5
SNORA2	7.5
PPIE	7.5
ZNF883	7.5
NLGN1	7.5

Gene Name	Fold change relative to normal
DBH-AS1	7.5
TMEM35	7.4
GPR37L1	7.4
MAP6D1	7.4
GCM1	7.4
MAPK8IP1	7.4
MKRN9P	7.4
AC092198.1	7.4
AC024592.9	7.4
CCDC150P1	7.4
RP11-96H17.3	7.4
GLYATL1P3	7.4
ATXN8OS	7.4
KHDC1	7.4
STARD6	7.4
CDCA7	7.4
AL160471.6	7.4
TDRD12	7.4
OR1H1P	7.3
RP11-746B8.1	7.3
RP11-457M11.5	7.3
MSL3P1	7.3
RP11-375B1.1	7.3
KCNH4	7.3
THEMIS3P	7.3
AC007966.1	7.3
CDT1	7.3
SKP2	7.3
CTD-2026G6.3	7.3
AC004593.3	7.3
KSR2	7.3
SPIN4	7.3
TMPO-AS1	7.3
Six3os1_4	7.3
CSTL1	7.3
PCBP3	7.3
EPHX4	7.3
ENHO	7.3
RMI2	7.3
SV2C	7.2
HIST2H2AB	7.2
AC078864.2	7.2
PKMYT1	7.2
BIK	7.2
NCAPG2	7.2
RP11-480I12.5	7.2
LINC01102	7.2
ATAD5	7.2
CIB2	7.2
CTC-340I23.2	7.2
SSX2IP	7.2

Gene Name	Fold change relative to normal
KCNIP3	7.2
CSMD2	7.2
CEACAM7	7.2
FTLP12	7.2
RP11-332J15.2	7.2
CCDC144CP	7.2
MT3	7.2
HYLS1	7.1
RP13-685P2.8	7.1
AC011242.6	7.1
GEMIN8P4	7.1
TBX15	7.1
DISP2	7.1
LINC00907	7.1
CDKAL1	7.1
GALNT8	7.1
RP11-26M5.3	7.1
KB-1836B5.1	7.1
KCNH1-IT1	7.1
LLNLR-268E12.1	7.1
RP11-5P18.10	7.1
PLK4	7.1
RP1-39G22.7	7.1
HIST1H1E	7.1
C3orf67	7.1
RP5-928E24.4	7.1
SV2B	7.1
RP11-21A7A.4	7.1
MTCL1	7.1
LRRN1	7.1
LINC00954	7.1
CTD-2165H16.4	7.1
RP11-111M22.4	7.0
HOXD9	7.0
RPL7AP26	7.0
PRKRIRP8	7.0
RP11-567G11.1	7.0
BDH1	7.0
RP11-22P6.3	7.0
BNIP3P27	7.0
ZNF92P3	7.0
SH2D6	7.0
RP11-70F11.8	7.0
FOXD2	7.0
HIST1H2AG	7.0
ZCCHC18	7.0
KPNA2	7.0
TUBB8	7.0
CARM1P1	7.0
SGOL2	7.0
FAM83C-AS1	7.0

Gene Name	Fold change relative to normal
STMN3	7.0
RP1-86C11.7	7.0
FANCA	7.0
LINC00661	6.9
AC003682.17	6.9
TMEM198	6.9
C10orf90	6.9
TCP10L	6.9
RP11-2E11.5	6.9
RP11-22B23.2	6.9
ENC1	6.9
DPY19L2P2	6.9
GREB1	6.9
AC092573.2	6.9
TTLL7-IT1	6.9
AL158069.1	6.9
PRC1-AS1	6.9
RP11-421G1.2	6.9
RP11-527L4.2	6.9
HIST3H2BB	6.9
CTD-2510F5.4	6.9
PCDHA8	6.9
KRT75	6.9
LRP4-AS1	6.9
NRIP3	6.9
PNMA5	6.9
RP11-423C15.3	6.9
ACTL6A	6.9
ADPRHL1	6.9
NUP62CL	6.9
SMC2	6.9
DCAF4L2	6.8
RP11-573M3.3	6.8
RPL39L	6.8
ZBTB20-AS4	6.8
FBXO5	6.8
RP11-438D14.3	6.8
PCSK1N	6.8
RP1-152L7.5	6.8
C1orf234	6.8
SYN2	6.8
HNRNPCP4	6.8
RASD2	6.8
PAQR4	6.8
AKAP8P1	6.8
RP3-407E4.3	6.8
HIST3H2BA	6.8
GGH	6.8
E2F1	6.8
RP11-829H16.3	6.8
IGFBP5	6.8

Gene Name	Fold change relative to normal
RP11-474P2.6	6.8
PAK3	6.8
RPL9P5	6.8
ZNF705G	6.8
DDN	6.8
LINC01280	6.8
SMCO2	6.7
RP1-101D8.1	6.7
RNFT2	6.7
RAD51	6.7
HOXB1	6.7
FAM72D	6.7
C3orf14	6.7
CTD-2377O17.1	6.7
RP11-91H12.4	6.7
CCDC28B	6.7
ENO2	6.7
HIST1H3I	6.7
STRCP1	6.7
HIST2H4A	6.7
SMKR1	6.7
TDGF1P7	6.7
WSCD1	6.7
HIST1H4L	6.7
AC079610.2	6.7
TMEM61	6.7
ADAMTS14	6.7
LINC01342	6.7
TRIT1	6.6
MESTP1	6.6
RP11-353N14.5	6.6
SPP1	6.6
HOXC4	6.6
RIMS4	6.6
RP11-114H23.2	6.6
FEN1	6.6
RP11-542G1.1	6.6
C2CD4A	6.6
RP5-1017F8.2	6.6
BCAN	6.6
AC133680.1	6.6
CTC-273B12.10	6.6
VANGL2	6.6
AC002310.17	6.6
TSPEAR	6.6
CCNF	6.6
FAM81A	6.6
FAR2P2	6.6
DFNB31	6.6
IGSF9B	6.6
WNT11	6.6

Gene Name	Fold change relative to normal
AC120042.1	6.6
AC104088.1	6.5
APOBEC3B	6.5
HMGB3P6	6.5
KIAA1211	6.5
ZNF878	6.5
RP11-30L15.4	6.5
LL09NC01-139C3	6.5
RP11-154J22.1	6.5
RP11-111M22.2	6.5
RP6-191P20.5	6.5
KIRREL3-AS2	6.5
RP4-621N11.2	6.5
RP11-410N8.4	6.5
RP11-166D18.1	6.5
C9orf163	6.5
CLIP3	6.5
MTL5	6.5
CENPW	6.5
CTD-2083E4.4	6.5
AC105402.2	6.5
PCDHB10	6.4
CENPV	6.4
RP11-586D19.1	6.4
RP11-146F11.5	6.4
HOXB3	6.4
RP11-565P22.2	6.4
MEX3B	6.4
SYT11	6.4
ARNTL2	6.4
VAX2	6.4
DDX12P	6.4
FBLN7	6.4
ARHGEF7-AS2	6.4
TXNDC16	6.4
RNF165	6.4
SLC35G2	6.4
RNU6-678P	6.4
FAM27E3	6.4
RP11-351C21.2	6.4
CPLX1	6.4
PCNA	6.4
RP11-484K9.4	6.4
ARHGAP19-SLIT1	6.4
SLC13A3	6.4
AL121578.2	6.3
SLC7A9	6.3
CCDC154	6.3
KALRN	6.3
RP11-269C23.5	6.3
RP11-317P15.4	6.3

Gene Name	Fold change relative to normal
HIST1H4K	6.3
FAM131B	6.3
CTD-2620I22.3	6.3
KRT80	6.3
C19orf57	6.3
ANKRD18A	6.3
ZNF252P-AS1	6.3
B4GALT6	6.3
GMNN	6.3
CDH8	6.3
CIART	6.3
CDCA4	6.3
MPP2	6.3
RP11-687F6.5	6.3
CTD-2340E1.2	6.3
RNU2-22P	6.3
RP11-480G7.2	6.3
RP11-44N12.5	6.2
RP11-687F6.1	6.2
EPHA10	6.2
OR1J1	6.2
KIAA0101	6.2
MTBP	6.2
RP11-132A1.6	6.2
HIST2H2BC	6.2
RP11-115C10.1	6.2
KIF18A	6.2
GAREML	6.2
VASH2	6.2
MAPK6PS3	6.2
RP11-34F20.7	6.2
GFRA4	6.2
SNAP25-AS1	6.2
RP4-738P11.4	6.2
GNRH2	6.2
AC097468.7	6.2
SCRT1	6.2
RP11-680F20.10	6.2
HOXD3	6.2
IL12RB2	6.2
RAD54B	6.2
ASPDH	6.2
SLC36A4	6.2
RP11-231I16.1	6.2
SDK2	6.2
MSI2	6.2
RP11-430E17.1	6.1
IPO9-AS1	6.1
TRIM36	6.1
PRTFDC1	6.1
MDK	6.1

Gene Name	Fold change relative to normal
FLJ12825	6.1
PSIP1	6.1
SLC16A14	6.1
CHRM5	6.1
RHEBL1	6.1
PDZD4	6.1
KLHL23	6.1
CKS1B	6.1
AC023593.1	6.1
DIRAS1	6.1
LINC01132	6.1
FANCG	6.1
ABLIM2	6.1
IZUMO4	6.1
OVOL3	6.1
AC073415.2	6.1
CTD-2349P21.6	6.1
GDAP1	6.0
RMI1	6.0
CTD-3105H18.9	6.0
SPTBN2	6.0
RBP1	6.0
OR13A1	6.0
RP11-174O3.3	6.0
HOXB7	6.0
KLF1	6.0
HFM1	6.0
C15orf41	6.0
NHS	6.0
C9orf84	6.0
FAIM2	6.0
TLL2	6.0
RP11-38J22.3	6.0
TMEM169	6.0
AC007204.2	6.0
LCT	6.0
HIST1H3D	6.0
AL353997.3	6.0
RP11-143K11.5	6.0
RP11-7I15.4	6.0
GRIP1	5.9
FAM72A	5.9
ARTN	5.9
MEGF11	5.9
RP11-634H22.1	5.9
AC004158.2	5.9
OXCT1	5.9
RP5-842K16.1	5.9
CTD-3222D19.12	5.9
CTD-3149D2.3	5.9
SNORA81	5.9

Gene Name	Fold change relative to normal
RP11-428P16.2	5.9
AK4	5.9
CTC-453G23.5	5.9
AP001626.2	5.9
FGF9	5.9
RP11-190P13.1	5.9
HOXA11	5.9
RP4-536B24.3	5.9
CRHR2	5.9
RP11-307O13.1	5.9
C19orf48	5.9
SPTBN4	5.9
HIST1H2AH	5.9
RP11-728F11.4	5.9
FAM3B	5.9
RP11-142E9.1	5.9
ASNS	5.9
RP11-977B10.2	5.8
FGF17	5.8
MCM6	5.8
SH2D7	5.8
RP11-192N10.2	5.8
RP11-161H23.10	5.8
CALHM1	5.8
RPL31P61	5.8
UCK2	5.8
RP11-895M11.2	5.8
RP11-98D18.9	5.8
RP11-973D8.5	5.8
RP11-95O2.1	5.8
ZFP69B	5.8
QPCT	5.8
TMEM132A	5.8
DSCC1	5.8
PLSCR2	5.8
TUBA1A	5.8
CTC-327F10.4	5.8
BRCA1	5.8
RP11-91I20.2	5.8
RP11-114H23.1	5.8
RP11-168F9.2	5.8
ATAD2	5.8
TBCAP2	5.8
RP11-142A23.1	5.8
NYAP1	5.7
GPR61	5.7
SALL4	5.7
IL17RD	5.7
MAD2L2	5.7
GSG2	5.7
TMEM179	5.7

**Gene Name**	**Fold change relative to normal**
RASL11B	5.7
RP11-159D12.11	5.7
TMEM106C	5.7
ARHGEF7-AS1	5.7
C1orf112	5.7
MYOM3	5.7
SOX4	5.7
SPTB	5.7
CTD-2256P15.2	5.7
RIMKLA	5.7
KIF3C	5.7
RP11-572C21.1	5.7
ADARB2	5.7
DNM1	5.7
TFDP1	5.7
ZNF840	5.7
AC093375.1	5.6
HNRNPA3P9	5.6
AC010980.2	5.6
Y_RNA	5.6
RP11-55K13.1	5.6
MND1	5.6
PDCL2	5.6
TARDBPP2	5.6
DNA2	5.6
GGTA2P	5.6
CKS1BP7	5.6
RP11-319E16.2	5.6
SNORA70	5.6
RP11-267D19.2	5.6
RP11-20I20.1	5.6
RP11-441F2.2	5.6
ACAN	5.6
CCDC136	5.6
FBXL16	5.6
AC092835.2	5.6
FZD3	5.6
RP4-758J18.13	5.6
AF038458.3	5.6
RP11-80H5.2	5.6
CRYGN	5.6
RNU2-11P	5.6
C16orf92	5.5
WIPF3	5.5
RP11-739L10.1	5.5
RP11-781A6.1	5.5
RHCE	5.5
CBFA2T2	5.5
AC003088.1	5.5
RP11-465B22.8	5.5
RETNLB	5.5

Gene Name	Fold change relative to normal
AC004381.6	5.5
RPS6KA6	5.5
RP11-707O23.5	5.5
RNA5SP323	5.5
DDX11	5.5
Six3os1_5	5.5
TCTEX1D2	5.5
RP11-421M1.8	5.5
AC006116.21	5.5
PRPH	5.5
FAM60CP	5.5
RRM1	5.5
LINC00594	5.5
NAALADL2-AS2	5.5
NPM2	5.5
AC105402.3	5.5
GPRC5B	5.5
RP11-173B14.4	5.5
NMNAT3	5.5
RP5-940J5.3	5.5
BARD1	5.4
DIAPH3	5.4
TRAIP	5.4
KCNB1	5.4
YEATS2-AS1	5.4
LINC00683	5.4
CENPL	5.4
MKRN7P	5.4
ARHGAP11A	5.4
RP11-133K1.7	5.4
HMGB2	5.4
MCM8	5.4
FGD1	5.4
CTD-2210P15.2	5.4
HRASLS	5.4
LINC01206	5.4
ARID3C	5.4
RP11-98D18.1	5.4
RP11-175P19.2	5.4
GS1-279B7.1	5.4
CDKN2B-AS1	5.4
CTD-2116N20.1	5.4
RP11-108M9.3	5.4
AC073321.4	5.4
C9orf40	5.4
RP11-18B3.2	5.4
HIF1A-AS1	5.4
RP11-369G6.2	5.4
RP5-1033H22.2	5.4
LINC00652	5.4
RP11-809H16.3	5.4

Gene Name	Fold change relative to normal
SLC12A5	5.4
RP11-168O16.1	5.4
CPE	5.3
RP5-1074L1.4	5.3
CTD-2582D11.1	5.3
AGPAT5	5.3
PPAT	5.3
RP11-230B22.1	5.3
FIGNL1	5.3
RP11-343N15.2	5.3
RNU7-75P	5.3
RP11-626I20.3	5.3
RP11-594N15.2	5.3
PYCR1	5.3
CKM	5.3
POLA1	5.3
PTGES3L	5.3
RP11-168A11.4	5.3
C12orf75	5.3
DONSON	5.3
MMS22L	5.3
GPSM2	5.3
IQCC	5.3
CEP97	5.3
RP6-65G23.3	5.3
LINC00925	5.3
RAC3	5.3
LL0XNC01-116E7	5.3
NT5M	5.3
PCLO	5.3
RP11-53B5.1	5.3
PCYT1B	5.3
AC011747.3	5.3
MRPL23-AS1	5.3
AC011747.4	5.3
RP11-53I6.4	5.2
FAM161A	5.2
RP11-406D1.2	5.2
RP1-228H13.5	5.2
RP11-266L9.1	5.2
MAP2	5.2
TAS2R38	5.2
CHN2	5.2
GLYATL1P4	5.2
TOPBP1	5.2
CTC-453G23.4	5.2
PHGDH	5.2
CTXN2	5.2
PRR19	5.2
RAB6B	5.2
BMP8A	5.2

Gene Name	Fold change relative to normal
CLTCL1	5.2
AC004076.5	5.2
AC093388.3	5.2
RP11-157D18.2	5.2
RP11-71H17.1	5.2
AC073621.2	5.2
RP11-188D8.1	5.2
BCYRN1	5.2
AC012360.4	5.2
FAM60BP	5.2
TMEM194A	5.2
RP11-210N13.1	5.2
AF064860.5	5.2
AC069257.6	5.2
HOXA-AS3	5.2
SMC4	5.1
ADAMTSL1	5.1
AC022182.2	5.1
PPP1R14D	5.1
RP11-814H16.2	5.1
RP11-307L3.4	5.1
CADM4	5.1
RP11-243J16.8	5.1
ZDHHC13	5.1
RPS6KL1	5.1
RP11-27N21.3	5.1
LL0XNC01-116E7	5.1
CRHR1-IT1	5.1
MURC	5.1
RP11-701I24.3	5.1
FMR1-AS1	5.1
RP11-434D11.4	5.1
APBA2	5.1
LINC01399	5.1
MAGEA8	5.1
NRTN	5.1
CCDC64	5.1
TMEM59L	5.1
ECE2	5.1
TMPO	5.1
MFSD6L	5.1
ZNF738	5.1
ADAM11	5.1
CHAF1A	5.1
HNRNPA1P16	5.1
RP11-696L21.2	5.1
AC068831.16	5.1
RP11-332J15.3	5.1
RP11-621H8.2	5.1
CACNA1C-AS1	5.0
RP11-334C17.6	5.0

**Gene Name**	**Fold change relative to normal**
RASGEF1C	5.0
RP11-421E14.2	5.0
SALL4P7	5.0
EMC3-AS1	5.0
RP11-63N3.1	5.0
AC020915.2	5.0
LINC00337	5.0
SLC7A4	5.0
FLJ37201	5.0
KLC3	5.0
CERS6	5.0
LAGE3P1	5.0
SPP2	5.0
MPHOSPH9	5.0
RP5-836N17.4	5.0
RP11-260M19.2	5.0
PROX1-AS1	5.0
CEP85	5.0
HMGA1P8	5.0
PLEK2	5.0
CTD-2529O21.1	5.0
BEX2	5.0
SNORA47	5.0
RP1-80N2.3	5.0
RP11-346J10.2	5.0
SLMO1	5.0
EIF3EP1	5.0
LINC00618	5.0
SNORA70	5.0
CTD-2562G15.3	5.0
PCDHB3	5.0
STK32A	5.0
PRIM1	5.0
HOXB-AS1	5.0
STAM-AS1	5.0
RP11-546B8.5	5.0
C16orf47	5.0
PSD	4.9
RP13-395E19.2	4.9
AC018705.5	4.9
RP5-1120P11.1	4.9
TEX30	4.9
C4orf46	4.9
RP5-908M14.10	4.9
LARP1P1	4.9
RP11-95G17.2	4.9
RP11-798M19.3	4.9
FRAS1	4.9
HIST1H2AB	4.9
AC078864.1	4.9
CTD-2521M24.6	4.9

Gene Name	Fold change relative to normal
PMAIP1	4.9
RP11-649A18.4	4.9
ZP3	4.9
CTD-2105E13.16	4.9
LIG1	4.9
SLFN13	4.9
EPHB2	4.9
RP11-404P21.3	4.9
RP11-599J14.2	4.9
NUAK1	4.9
AC140481.8	4.9
CTC-276P9.4	4.9
MYEF2	4.9
FRMPD3	4.9
ZNF530	4.9
IL22RA1	4.9
SRCRB4D	4.9
RP11-393I2.4	4.9
RAB19	4.9
ANO9	4.9
ATP8A2	4.9
C4orf50	4.9
SLC7A11	4.9
C21orf58	4.9
TRIM46	4.9
ZNF713	4.9
DLEU2	4.9
HOXB4	4.8
RFC5	4.8
FETUB	4.8
FAXC	4.8
HMGN2P4	4.8
ASS1P2	4.8
RP11-635N19.2	4.8
AC015933.2	4.8
GPR63	4.8
CGREF1	4.8
RP11-13K12.1	4.8
RP3-486I3.7	4.8
SUV39H2	4.8
GOLGA7B	4.8
FSD1L	4.8
ATP6V0E2-AS1	4.8
BRI3BP	4.8
RPL18P10	4.8
TMCC2	4.8
KATNAL2	4.8
ZNF625	4.8
NR0B2	4.8
MYRIP	4.8
ANKRD13B	4.8

Gene Name	Fold change relative to normal
NUP155	4.8
RP3-428L16.2	4.8
HIST1H2BE	4.8
CEP78	4.8
RNF6P1	4.8
SLC29A4	4.8
CELSR2	4.8
HSF2BP	4.8
SYNJ2	4.8
RP11-342A23.1	4.8
RP3-322G13.7	4.8
RP11-849F2.7	4.8
OLIG3	4.8
MESP1	4.8
YWHAZP4	4.8
RP11-489G11.3	4.8
RP11-383I23.2	4.8
LZTS1	4.8
AC159540.2	4.8
DNMT3B	4.8
DSP	4.8
CTHRC1	4.7
RP11-94H18.1	4.7
SKA2	4.7
INCENP	4.7
SRD5A1	4.7
RP11-312J18.5	4.7
GLP1R	4.7
ADCY5	4.7
POLE	4.7
CTB-92J24.2	4.7
CA8	4.7
NCAPD3	4.7
AC012363.8	4.7
LL0XNC01-116E7	4.7
PAX6	4.7
RP11-435J9.2	4.7
TUBB4BP2	4.7
AC002116.7	4.7
RP11-530N7.2	4.7
CTD-2574D22.2	4.7
RP5-1025A1.3	4.7
RBFADN	4.7
UNG	4.7
SUV420H2	4.7
RP11-535M15.2	4.7
AMOT	4.7
SLC38A1	4.7
RP11-281P23.2	4.7
SASS6	4.7
RP4-726N1.2	4.7

Gene Name	Fold change relative to normal
RP11-889L3.4	4.7
RP3-337H4.8	4.7
PSMC3IP	4.7
DUSP4	4.7
RP11-264C15.2	4.7
STRIP2	4.6
GUSBP8	4.6
RP11-1C8.5	4.6
CTD-2547G23.4	4.6
MCM7	4.6
BSN-AS2	4.6
RP11-77E14.2	4.6
AC007461.2	4.6
TNFRSF11A	4.6
COL6A4P1	4.6
RP11-196G18.22	4.6
RP4-777D9.2	4.6
HSD11B2	4.6
TUBB2A	4.6
IBSP	4.6
FBXO45	4.6
ERC2	4.6
XXbac-B562F10.1	4.6
CBX1	4.6
DBF4	4.6
LRRN2	4.6
NKPD1	4.6
RBBP4P4	4.6
AC011523.2	4.6
TTC9B	4.6
RP11-219B4.7	4.6
SEC61A2	4.6
HS3ST1	4.6
AC074117.13	4.6
TCP11X2	4.6
MNS1	4.6
ZMPSTE24	4.6
Y_RNA	4.6
GYLTL1B	4.6
RPL7AP2	4.5
CTB-102L5.8	4.5
DPYSL3	4.5
BRCA2	4.5
POLR3G	4.5
RP11-3B12.4	4.5
CENPN	4.5
AKR7L	4.5
RP11-87C12.5	4.5
SDK1	4.5
RP11-412D9.4	4.5
RP11-274H2.3	4.5

Gene Name	Fold change relative to normal
SSBP3-AS1	4.5
C1orf74	4.5
RP11-435O5.6	4.5
RP11-560J1.2	4.5
RP11-197P3.5	4.5
RP11-10O17.1	4.5
RP11-187A9.3	4.5
GBAP1	4.5
RP11-839D17.3	4.5
BAALC	4.5
ZC3HAV1L	4.5
RP4-751H13.7	4.5
DCLK2	4.5
FRMPD1	4.5
RP11-301O19.1	4.5
CBX8	4.5
CCDC87	4.5
NME1	4.5
H2AFX	4.4
CIT	4.4
ZNF8	4.4
FSTL4	4.4
AC114803.3	4.4
RP11-334G22.1	4.4
RP11-386G11.10	4.4
SATB2	4.4
MSH2	4.4
RN7SL381P	4.4
CDC20P1	4.4
RN7SL449P	4.4
RP11-25B7.1	4.4
RP11-181B11.1	4.4
PCA3	4.4
DBF4P1	4.4
RP11-70L8.4	4.4
MYADML2	4.4
UPF3B	4.4
RP5-943J3.2	4.4
TMEM246	4.4
CTPS1	4.4
MXD3	4.4
RP11-74C1.4	4.4
TUBBP2	4.4
AC083884.8	4.4
C18orf54	4.4
NCS1	4.4
CTC-444N24.13	4.4
SYTL4	4.4
RP11-325L7.1	4.4
RP11-506M12.1	4.4
RP11-332J15.1	4.4

**Gene Name**	**Fold change relative to normal**
IGFL1P1	4.4
NARF-IT1	4.4
ZGRF1	4.4
RP11-2E11.9	4.4
DIRC3-AS1	4.4
RP3-467N11.1	4.4
TRAPPC13P1	4.4
FNDC7	4.4
SLC16A1	4.3
ANKRD20A1	4.3
NAV1	4.3
RP11-544A12.4	4.3
AC006262.11	4.3
RP11-689C9.1	4.3
RP11-386G11.5	4.3
SLC4A8	4.3
PPM1H	4.3
USP49	4.3
POMC	4.3
RP11-469L4.1	4.3
KCTD21-AS1	4.3
HNRNPA3P3	4.3
RP11-272P10.2	4.3
RP11-401N16.2	4.3
IGLVIVOR22-1	4.3
RP11-559M23.1	4.3
CDK2	4.3
ANKRD32	4.3
HTR6	4.3
RGAG1	4.3
FDPSP8	4.3
RP11-305P22.9	4.3
RP11-435D7.3	4.3
MZT1	4.3
PHF6	4.3
CTD-3138B18.6	4.3
OXCT1-AS1	4.2
PGF	4.2
RP11-18H7.1	4.2
PLEKHG5	4.2
RP11-312B8.1	4.2
RP11-705O1.8	4.2
RP5-1024G6.8	4.2
STK24-AS1	4.2
PRUNE2	4.2
CHTF18	4.2
ZNF681	4.2
RP1-274L7.4	4.2
CALR4P	4.2
LINC01301	4.2
SEZ6L2	4.2

Gene Name	Fold change relative to normal
CYP4F8	4.2
SLC2A1-AS1	4.2
MCM3	4.2
ATP6V1C2	4.2
RP11-16N11.2	4.2
ZNF93	4.2
HOXB-AS2	4.2
MCF2L-AS1	4.2
DNAH14	4.2
RP11-1012A1.7	4.2
RNU6-658P	4.2
SNORD88A	4.2
C16orf87	4.2
RP11-376M2.2	4.2
AC010642.1	4.2
FAM155A	4.2
SPDL1	4.2
AC006042.6	4.2
CDK2AP2P2	4.2
TNFRSF25	4.2
HMGN2P3	4.2
SLC2A1	4.2
PTPRN2	4.2
AC139099.5	4.2
TSHZ2	4.2
RP11-831A10.1	4.2
SLC26A5	4.2
ZWILCH	4.2
LMNB2	4.2
RP11-466A19.3	4.2
RP11-466A19.8	4.2
KCNH1	4.2
LGI2	4.2
DHFRP1	4.2
CENPH	4.2
VN1R110P	4.2
RP11-152N13.5	4.2
U47924.29	4.2
PDK1	4.1
PCDHB14	4.1
IGFBP2	4.1
AC135050.5	4.1
KCND3	4.1
TK1	4.1
HAUS1P3	4.1
DBF4B	4.1
MCTS2P	4.1
NT5DC3	4.1
TUBG1	4.1
PI15	4.1
ZNF749	4.1

Gene Name	Fold change relative to normal
RP13-455A7.1	4.1
SMS	4.1
ZNF726	4.1
ZNF793	4.1
RNU2-69P	4.1
HAUS5	4.1
PLXNB3	4.1
PM20D2	4.1
VPS37D	4.1
LRP8	4.1
ZNF519	4.1
EGLN3	4.1
HMGN2	4.1
PLCB1	4.1
CACNB3	4.1
RP11-21B23.1	4.1
RCC2	4.1
PDCD6IPP2	4.1
RP11-335E6.3	4.1
RP11-398C13.6	4.1
LA16c-358B7.4	4.1
RP11-385M4.3	4.0
RP4-800J21.3	4.0
FLJ41941	4.0
AC093901.1	4.0
PVRL1	4.0
MDM1	4.0
KIF22	4.0
RP11-184M15.2	4.0
RP11-108M9.6	4.0
RP11-746P2.3	4.0
ZNF850	4.0
RP11-52A20.2	4.0
ZNF714	4.0
CTA-390C10.9	4.0
ICA1L	4.0
HIST1H2BL	4.0
USP1	4.0
GOLGA2P8	4.0
RP11-374M1.2	4.0
RP11-1E4.1	4.0
DNMT1	4.0
NEURL1B	4.0
HAUS1	4.0
CROCCP1	4.0
HYDIN2	4.0
SLC7A1	4.0
GKAP1	4.0
RP11-516A11.1	4.0
NARF	4.0
PSPH	4.0

**Gene Name**	**Fold change relative to normal**
RP13-735L24.1	4.0
NFYC-AS1	4.0
RP11-650J17.1	4.0
RP4-665J23.2	4.0
ICA1	4.0
C14orf37	4.0
MCOLN3	4.0
CTD-2349P21.11	4.0
OR7E14P	4.0
SETSIP	4.0
ZNF525	4.0
AC079753.5	4.0
RP4-730K3.3	4.0
SAAL1	4.0
RP11-481A20.11	4.0
SRRM5	4.0
RP11-110I1.12	4.0
TTLL7	4.0
CTD-2203K17.1	4.0
FAM60A	4.0
GMPS	4.0
CTD-2555O16.3	3.9
CAPN10-AS1	3.9
TPI1P3	3.9
RP11-376O6.2	3.9
TPBGL	3.9
MIR1289-1	3.9
GINS3	3.9
RP11-115C21.2	3.9
SLC7A5	3.9
LRRFIP1P1	3.9
LARGE-AS1	3.9
RP11-656D10.3	3.9
SEPT5	3.9
SLC4A3	3.9
RP1-168L15.5	3.9
HPRT1	3.9
HSF2	3.9
C2ORF15	3.9
MAMSTR	3.9
NUDT19P3	3.9
NBEA	3.9
HNRNPA3P10	3.9
PGAP1	3.9
GLT1D1	3.9
RP11-573D15.8	3.9
ITGB3BP	3.9
AC068831.17	3.9
RP11-2E11.10	3.9
RP11-12G12.7	3.9
ASB16	3.9

**Gene Name**	**Fold change relative to normal**
AC016831.6	3.9
CENPO	3.9
NCAPD2	3.9
FNBP1P1	3.9
SMAD9	3.9
HSPA4L	3.9
QSOX2	3.9
ANK2	3.9
RP11-613M5.1	3.8
RP11-398K22.12	3.8
MAFG-AS1	3.8
PLOD2	3.8
CHST1	3.8
LINC00449	3.8
SGSM1	3.8
RP11-118E18.2	3.8
POC1A	3.8
ZNF233	3.8
VN1R108P	3.8
OR7E116P	3.8
ZNF322	3.8
EMC9	3.8
CYP46A1	3.8
RP11-22C11.2	3.8
AK4P3	3.8
RP11-602N24.3	3.8
CCDC138	3.8
PAICS	3.8
AC012363.10	3.8
HDAC2	3.8
ZNF572	3.8
DTYMK	3.8
DLX2-AS1	3.8
AC024937.4	3.8
RAB3A	3.8
CEP57L1	3.8
UBAC2-AS1	3.8
XXYLT1	3.8
DHFR	3.8
AP001046.5	3.8
AL138935.1	3.8
KRT8P39	3.8
C3orf70	3.8
CKAP5	3.8
ZBED8	3.8
RPL12P37	3.8
SHCBP1	3.8
ZNF385C	3.7
HAUS6	3.7
CTB-55O6.10	3.7
ELOVL7	3.7

Gene Name	Fold change relative to normal
SRGAP1	3.7
TMTC4	3.7
GAPDHP46	3.7
CEP89	3.7
CTD-2349P21.9	3.7
RP11-262H14.5	3.7
ZNF551	3.7
PARP1	3.7
RP4-761J14.9	3.7
RP11-977G19.14	3.7
MTHFD2	3.7
RGAG4	3.7
RP11-644C3.1	3.7
DHRS13	3.7
IMMP1L	3.7
TRIM37	3.7
ZFP30	3.7
RUVBL1	3.7
ZDHHC15	3.7
SRRM2-AS1	3.7
ULBP1	3.7
RP11-348M3.2	3.7
XPOTP1	3.7
IPO5P1	3.7
G2E3	3.7
AP001619.3	3.7
BORA	3.7
RP11-134K13.2	3.7
NFASC	3.7
KIAA1024	3.7
KDM1A	3.7
SPATS2	3.7
COQ3	3.7
JAKMIP1	3.7
LYSMD1	3.7
RP11-552F3.12	3.7
ARHGAP39	3.6
LEF1-AS1	3.6
ZSCAN23	3.6
LPGAT1	3.6
ASRGL1	3.6
JADE3	3.6
POLA2	3.6
CEP19	3.6
HIST1H2BD	3.6
RP11-632L2.2	3.6
THOC7-AS1	3.6
OCIAD2	3.6
CTD-2619J13.16	3.6
FANCC	3.6
NCR3LG1	3.6

**Gene Name**	**Fold change relative to normal**
PIPOX	3.6
CCDC14	3.6
FLVCR1	3.6
GATS	3.6
MTFR2	3.6
PRKAA2	3.6
DDX39A	3.6
CTC-559E9.8	3.6
FA2H	3.6
TUSC3	3.6
RP11-409K20.7	3.6
DDIAS	3.6
RBM38	3.6
MAST2	3.6
SOGA1	3.6
RP4-739H11.3	3.6
RNU6-97P	3.6
RP11-375N15.2	3.6
PHF8	3.6
CREB3L4	3.6
JAM3	3.6
RP11-71H17.9	3.6
LINC01240	3.6
TSPY26P	3.6
LRR1	3.6
APOO	3.6
EFS	3.6
MSH6	3.6
PLEKHA6	3.6
EPB41	3.6
CHD7	3.6
WDR34	3.6
RAP1GAP2	3.6
ZEB1-AS1	3.6
SETBP1	3.6
RP1-167A14.2	3.6
PTOV1-AS1	3.6
ABCA5	3.6
PRDX2	3.6
KANTR	3.5
RP11-1023L17.1	3.5
ZNF43	3.5
ATP13A3	3.5
ERO1LB	3.5
USP37	3.5
TONSL	3.5
BOLA3-AS1	3.5
CNTLN	3.5
CBX5	3.5
PHF19	3.5
SDR42E1	3.5

Gene Name	Fold change relative to normal
BTBD3	3.5
HMGB1P14	3.5
PDRG1	3.5
HDGFL1	3.5
TDP1	3.5
RP11-3B12.2	3.5
RP11-706O15.1	3.5
SLC1A4	3.5
TOX	3.5
BLM	3.5
RP11-815I9.4	3.5
RP11-580I16.2	3.5
UBA2	3.5
HOOK1	3.5
PCDHB16	3.5
VRK1	3.5
RP11-761I4.5	3.5
DBNDD1	3.5
NANOS1	3.5
NNT-AS1	3.5
RP11-49C9.2	3.5
SMC1A	3.5
INTS7	3.5
FAM122B	3.5
GAS2L3	3.5
PRMT6	3.5
ETNK2	3.5
PTGER4P2	3.5
MAPK12	3.4
MCF2L2	3.4
CTD-2528A14.3	3.4
CKS2	3.4
NDC1	3.4
RP4-736L20.3	3.4
NAPB	3.4
TDGP1	3.4
DKC1	3.4
SHF	3.4
OVOL2	3.4
BHLHB9	3.4
RP11-278A23.4	3.4
SKAP1	3.4
CAMK1D	3.4
ZDHHC23	3.4
RP5-935K16.1	3.4
PAIP2B	3.4
NUP210	3.4
ZNF92	3.4
PTTG3P	3.4
DCLK1	3.4
PGM2L1	3.4

Gene Name	Fold change relative to normal
DEK	3.4
SHMT2	3.4
ACVR1C	3.4
SLC25A15	3.4
TUBAP2	3.4
LINC01002	3.4
CEP131	3.4
ALYREF	3.4
PRIM2	3.4
ROBO1	3.4
RNU4-39P	3.4
SLC22A17	3.4
GJC1	3.4
MAP1A	3.4
GUSBP2	3.4
BEND3	3.4
AC012512.1	3.4
TMEM201	3.4
PPP1R14B	3.4
PAICSP3	3.4
FAM24B	3.4
CDKN2D	3.4
ARL6IP6	3.4
CENPQ	3.4
MYL6B	3.3
HAUS8	3.3
CTC-137K3.1	3.3
RP11-433O3.1	3.3
AP001469.9	3.3
MIS18A	3.3
LDLRAD3	3.3
KIT	3.3
PPFIA3	3.3
UQCC2	3.3
RPL7P17	3.3
RP11-22B23.1	3.3
HMGA1P2	3.3
QSER1	3.3
RP11-65D24.2	3.3
HCN3	3.3
CMSS1	3.3
RP11-395L14.17	3.3
CEP72	3.3
ZC3H8	3.3
BTNL10	3.3
CTC-244M17.1	3.3
CDK4	3.3
RP11-138P22.1	3.3
KCTD1	3.3
IFT81	3.3
MCF2L	3.3

Gene Name	Fold change relative to normal
ZNF587	3.3
TOB2P1	3.3
TMOD2	3.3
MAP2K6	3.3
HIST2H2BE	3.3
ZNF543	3.3
RBBP4P1	3.3
PLCB4	3.3
GATSL2	3.3
PPP1R14BP3	3.3
TBCCD1	3.3
FBXO16	3.3
GLB1L2	3.3
MIR1302-3	3.3
NFIB	3.3
RCC1	3.3
RAB3IP	3.3
UBXN7	3.3
BCL2	3.3
NABP2	3.3
HLTF	3.3
TSPYL5	3.2
ZNF138	3.2
NONO	3.2
FADS2	3.2
RBBP7	3.2
C12orf73	3.2
MTND3P10	3.2
ZNF473	3.2
PNMA1	3.2
RP11-798M19.6	3.2
ZNF286A	3.2
WDR90	3.2
NASP	3.2
KNSTRN	3.2
RHPN2	3.2
PIGX	3.2
H2AFY2	3.2
URB2	3.2
RP4-680D5.8	3.2
TMEM44	3.2
PMEL	3.2
RN7SL396P	3.2
SUZ12P1	3.2
ZNF280C	3.2
H2AFZ	3.2
LINC00526	3.2
RFWD3	3.2
BOC	3.2
CCP110	3.2
SMIM17	3.2

Gene Name	Fold change relative to normal
RN7SL569P	3.2
DSN1	3.2
FBXO32	3.2
CCDC15	3.2
RBBP8	3.2
VAV2	3.2
KIAA0907	3.2
TMEM108	3.2
SH3BP4	3.2
YEATS4	3.2
LSM7	3.2
EID2B	3.2
TMEM150C	3.2
PAXBP1-AS1	3.2
SLC16A8	3.2
CTD-2368P22.1	3.2
RP11-440L14.1	3.2
PARD6G	3.2
IDH2	3.1
KDELC1	3.1
KLF12	3.1
NCBP1	3.1
DBN1	3.1
GFI1	3.1
ZNF620	3.1
SSBP3	3.1
PFKP	3.1
CCDC167	3.1
EFR3B	3.1
RSRC1	3.1
RNF219	3.1
RP11-598P20.3	3.1
PRPS2	3.1
ZNF680	3.1
KCNC4	3.1
KCNJ11	3.1
ZNF669	3.1
MRPS31P4	3.1
TDG	3.1
ILF2	3.1
ZNF606	3.1
POLR2H	3.1
GLS2	3.1
OLFM1	3.1
FKBP4	3.1
BSPRY	3.1
C19orf40	3.1
SPPL2B	3.1
KIAA1586	3.1
DNAJC3-AS1	3.1
ZNF253	3.1

Gene Name	Fold change relative to normal
ZNF544	3.1
CA14	3.1
RP11-728K20.3	3.1
GNN	3.1
RBBP4	3.1
SPICE1	3.1
RP11-292F9.1	3.1
ZNF550	3.1
DNAJC9	3.1
ZNF708	3.1
TIPIN	3.1
GPX7	3.1
CTD-3193O13.8	3.0
RHPN1	3.0
NARS2	3.0
CTD-2293H3.2	3.0
CTC-359D24.3	3.0
OPA1	3.0
PGK1P2	3.0
SS18L1	3.0
GPR180	3.0
CABLES2	3.0
CTD-2561J22.2	3.0
RP3-508I15.9	3.0
RPGRIP1L	3.0
FANCL	3.0
RP4-585I14.3	3.0
CTD-2583A14.8	3.0
WDR53	3.0
GS1-257G1.1	3.0
SEMA6C	3.0
AP3M2	3.0
WHSC1	3.0
RP11-160O5.1	3.0
TFAP4	3.0
FKBP3	3.0
TMEM97	3.0
ZRANB3	3.0
KPTN	3.0
INSR	3.0
FADS1	3.0
AC004987.9	3.0
RFC2	3.0
EPT1	3.0
TACC3	3.0
TMEM14A	3.0
TPD52	3.0
ZNF221	3.0
SENP1	3.0
C11orf84	3.0
PRKDC	3.0

Gene Name	Fold change relative to normal
MAGEF1	3.0
SRPK1	3.0
PCNXL2	3.0
MARCH6	3.0
SLC39A6	3.0
SNRPD1	3.0
DARS2	3.0
MARCKS	3.0
SORD	3.0
FOXRED2	3.0
METTL4	3.0
BRIX1	3.0
PARP2	3.0
CNKSR2	3.0
ARHGEF19	3.0
ENAH	3.0
LIN9	3.0
CEP76	3.0
ZFP37	3.0
SNRPB	3.0
XRCC3	2.9
ALMS1	2.9
NREP	2.9
HN1L	2.9
C17orf75	2.9
GLMN	2.9
ANP32E	2.9
TMEM38B	2.9
ZNF334	2.9
NAA40	2.9
SAE1	2.9
TBPL1	2.9
ZNF594	2.9
VBP1	2.9
PTBP2	2.9
FANCF	2.9
CTD-3138B18.5	2.9
RP11-15H20.6	2.9
ING1	2.9
NRSN2	2.9
ZNF137P	2.9
KIF21A	2.9
RP11-1033A18.1	2.9
APOA1BP	2.9
ATL1	2.9
LBR	2.9
C10orf35	2.9
BNIP3	2.9
TAF9B	2.9
CCDC18	2.9
CACYBP	2.9

**Gene Name**	**Fold change relative to normal**
SOX12	2.9
ZNF347	2.9
AC024560.3	2.9
RP11-27I1.4	2.9
NR2C2AP	2.9
TUBBP1	2.9
ZNF260	2.9
TTF2	2.9
TFDP2	2.9
POT1-AS1	2.9
PROSER3	2.9
TUBA1B	2.9
EFCAB7	2.8
IQCB1	2.8
ZNF227	2.8
IPO9	2.8
RNF168	2.8
SNAPC3	2.8
RAE1	2.8
RPRD1A	2.8
R3HDM1	2.8
C1orf109	2.8
TMEM237	2.8
MAP3K14-AS1	2.8
RHNO1	2.8
GEMIN2	2.8
LIG3	2.8
GDI1	2.8
ZNF491	2.8
EXOSC2	2.8
RAD51C	2.8
PLEKHH1	2.8
NAT14	2.8
GPR161	2.8
TBC1D30	2.8
SFXN1	2.8
ZNF81	2.8
RUSC1	2.8
ACVR2A	2.8
CASP8AP2	2.8
EXOSC5	2.8
LIN52	2.8
MTHFD1	2.8
NOP56	2.8
TMEM206	2.8
LMBRD2	2.8
PRPS1	2.8
AACS	2.8
RTTN	2.8
LSG1	2.8
PIAS2	2.8

Gene Name	Fold change relative to normal
SENP5	2.8
TTC5	2.8
STK39	2.8
CSTF3	2.8
PGBD1	2.8
FBXO41	2.8
TPI1P1	2.8
E2F3	2.8
CCDC23	2.8
ARHGEF7	2.8
CENPP	2.8
RIC8B	2.8
CCT5	2.8
TSPYL4	2.8
GNL3L	2.8
RCCD1	2.8
CEP152	2.8
ERH	2.7
SMYD3	2.7
CENPJ	2.7
NNT	2.7
ATG4D	2.7
LFNG	2.7
PXYLP1	2.7
DCTN3	2.7
ANAPC11	2.7
TBC1D31	2.7
CBX3P9	2.7
PPP5C	2.7
PIGCP1	2.7
KIAA1731	2.7
NUP205	2.7
DROSHA	2.7
RBL1	2.7
SAC3D1	2.7
WRAP73	2.7
THOP1	2.7
POU2F1	2.7
STRA13	2.7
ZNF121	2.7
BRWD3	2.7
GAPDH	2.7
CYB5RL	2.7
ZNF772	2.7
SLC25A14	2.7
SUV39H1	2.7
HN1	2.7
GAS5-AS1	2.7
PHF13	2.7
L2HGDH	2.7
KIF2A	2.7

Gene Name	Fold change relative to normal
DCTPP1	2.7
ASB16-AS1	2.7
DCAKD	2.7
RNF138	2.7
YEATS2	2.7
NDUFA7	2.7
SP4	2.7
STRBP	2.7
GTF3C5	2.7
AGAP1	2.7
POLD1	2.7
NUCKS1	2.7
CHML	2.7
TSEN54	2.7
SRP9	2.7
XPR1	2.7
ZBTB18	2.7
ZNF107	2.7
ANGPT2	2.6
PAPD7	2.6
ZNF480	2.6
MASTL	2.6
CCDC77	2.6
SNRNP25	2.6
ANKEF1	2.6
HTATSF1	2.6
TIMM50	2.6
XPO5	2.6
ZNF232	2.6
SUZ12	2.6
ZBTB39	2.6
PHKA1	2.6
YPEL1	2.6
FYTTD1	2.6
NUP85	2.6
NUP62	2.6
VANGL1	2.6
PKIA	2.6
DTX3	2.6
ZNF3	2.6
C11orf80	2.6
SLC25A13	2.6
SAMD1	2.6
GRAMD1B	2.6
HSP90AA1	2.6
PJA1	2.6
SNRPE	2.6
C17orf80	2.6
EIF4A3	2.6
TRERF1	2.6
POLE3	2.6

Gene Name	Fold change relative to normal
NT5DC2	2.6
TDRKH	2.6
LPHN1	2.6
TMEM176A	2.6
ATP5G1	2.6
MED14	2.6
PRPF4	2.6
TMEM260	2.6
INTS2	2.6
ZNF254	2.6
FAF1	2.6
NOL11	2.6
PIAS3	2.6
JAG2	2.6
RP1-283E3.8	2.6
MED27	2.6
GADD45G	2.6
ODF2	2.6
GPC6	2.6
AC019097.7	2.6
ARL6IP1	2.6
KIFC2	2.6
KPNB1	2.6
DHX36	2.6
CHEK2	2.6
TUFT1	2.6
ZNF611	2.6
EXTL3	2.6
KIF20B	2.6
CSE1L	2.6
PSD3	2.6
PER3	2.6
GGCT	2.5
SETP14	2.5
SOCS4	2.5
B4GALT2	2.5
SLX4IP	2.5
CDCA7L	2.5
U2SURP	2.5
EPCAM	2.5
ZMYM3	2.5
PGAM5	2.5
TMEM38A	2.5
C11orf30	2.5
LINC00680	2.5
C2CD3	2.5
ATP7B	2.5
SUPT16H	2.5
CAND1	2.5
ZNF417	2.5
KDM1B	2.5

Gene Name	Fold change relative to normal
DYRK2	2.5
DCUN1D2	2.5
CCT3	2.5
NUP107	2.5
VPS72	2.5
RTN3P1	2.5
LRRC8B	2.5
FUZ	2.5
BCOR	2.5
HDGFRP3	2.5
DLG3	2.5
USP11	2.5
RP11-156P1.3	2.5
PHTF1	2.5
BRD3	2.5
CSTF2	2.5
ZNF428	2.5
C12orf65	2.5
ZNF776	2.5
TBC1D16	2.5
GOLM1	2.5
TARS	2.5
SNRNP40	2.5
IRGQ	2.5
GTPBP3	2.5
DOLPP1	2.5
PTMA	2.5
KIAA1549	2.5
EIF1AXP1	2.5
TRIM28	2.5
WDR31	2.5
KIAA1467	2.5
SRM	2.5
FANCE	2.5
KIAA1841	2.5
RIF1	2.5
UBE2E3	2.5
CDC25B	2.5
BPHL	2.5
KDF1	2.5
CDK2AP1	2.4
HACE1	2.4
RQCD1	2.4
IFT122	2.4
IVNS1ABP	2.4
TIAM1	2.4
SMC3	2.4
AIF1L	2.4
ANKRD18EP	2.4
TRMT6	2.4
BCL9	2.4

Gene Name	Fold change relative to normal
OCRL	2.4
ACAP3	2.4
PROSER1	2.4
ACVR2B	2.4
LSM14B	2.4
GDF11	2.4
XPOT	2.4
TMED8	2.4
CARHSP1	2.4
DHX40	2.4
CTDSPL2	2.4
ALG6	2.4
TTLL5	2.4
PTOV1	2.4
STOML2	2.4
SUGP2	2.4
PHTF2	2.4
PPP1R3E	2.4
CLSTN1	2.4
OLA1	2.4
AHI1	2.4
SPIRE2	2.4
FBXL19-AS1	2.4
LSM4	2.4
HPSE	2.4
LRRC58	2.4
RPA2	2.4
NGFRAP1	2.4
CBX3	2.4
PDIK1L	2.4
CTPS2	2.4
ZNF184	2.4
TBCB	2.4
ERI3	2.4
IPP	2.4
POT1	2.4
MAZ	2.4
INPPL1	2.4
DVL3	2.4
SSRP1	2.4
MBNL3	2.4
EXOC5	2.4
DCAF16	2.4
FYN	2.4
HSPA14	2.4
C7orf73	2.4
ILF3	2.4
FARSB	2.4
PPME1	2.4
AP4M1	2.4
PGK1	2.3

Gene Name	Fold change relative to normal
PLEKHJ1	2.3
H2AFV	2.3
TAF2	2.3
TNPO2	2.3
SF3B3	2.3
CASP2	2.3
SIKE1	2.3
CKB	2.3
TADA1	2.3
FAM136A	2.3
ZNF48	2.3
SMG9	2.3
LRRC20	2.3
RBMX	2.3
MORF4L2	2.3
ZNF292	2.3
ZNF675	2.3
PTAR1	2.3
COIL	2.3
ARHGEF16	2.3
TUBGCP3	2.3
CEBPG	2.3
PRMT1	2.3
RANBP1	2.3
TBL1XR1	2.3
SHFM1	2.3
ZBTB41	2.3
DNMT3A	2.3
KNOP1	2.3
ZNF275	2.3
WDR5	2.3
CMIP	2.3
PYGO2	2.3
PRUNE	2.3
TCF3	2.3
BUB3	2.3
HDAC1	2.3
NAB1	2.3
USP18	2.3
SGPL1	2.3
ZNF664	2.3
SLC25A40	2.3
RALGPS1	2.3
CPSF6	2.3
KIAA1715	2.3
HSPD1	2.3
NUP43	2.3
ZNF124	2.3
PHC1	2.3
FLAD1	2.3
ST14	2.3

Gene Name	Fold change relative to normal
CDK8	2.3
DCAF12	2.2
NLN	2.2
UMPS	2.2
ATXN7L3B	2.2
HINFP	2.2
TPI1	2.2
H1FX	2.2
FMR1	2.2
NEDD1	2.2
PLEKHB1	2.2
RAD21	2.2
PPM1D	2.2
U2AF2	2.2
CDK19	2.2
PRMT5	2.2
AP1S1	2.2
IPO5	2.2
MRPS26	2.2
GPD2	2.2
RAD1	2.2
KDM5B	2.2
CCT2	2.2
KLHL42	2.2
PRPF19	2.2
BANF1	2.2
PDZD11	2.2
VPS35	2.2
UNK	2.2
STAG2	2.2
RAVER2	2.2
SET	2.2
PHIP	2.2
TXNL4A	2.2
CERS4	2.2
HNRNPR	2.2
ENOPH1	2.2
HAX1	2.2
PRDX4	2.2
HDGF	2.2
TSN	2.2
PKP4	2.2
FOXRED1	2.2
POMT2	2.2
SPAST	2.1
EXTL2	2.1
FAM127B	2.1
PCCB	2.1
RBBP5	2.1
ZCCHC11	2.1
BTF3L4	2.1

Gene Name	Fold change relative to normal
FXR1	2.1
RNF144A	2.1
PCNXL4	2.1
SUMO2	2.1
PPIF	2.1
DNAJB11	2.1
TROVE2	2.1
ZBTB33	2.1
PHLPP1	2.1
NUCB2	2.1
NUP188	2.1
KLHDC3	2.1
HNRNPA3	2.1
UBE2O	2.1
GSK3B	2.1
TOP3A	2.1
PNN	2.1
ELK1	2.1
HNRNPL	2.1
ARID2	2.1
VPS45	2.1
SLC44A1	2.1
PREP	2.1
TMEM183A	2.1
SLBP	2.1
WSB2	2.1
H3F3A	2.1
HCFC1	2.1
NAA50	2.1
MORC4	2.1
MRPL37	2.1
FUBP1	2.1
YWHAQ	2.1
COPG2	2.1
ZNF326	2.1
S100PBP	2.1
PAXIP1	2.1
ELAVL1	2.1
MAGED1	2.1
NUDT21	2.1
HYOU1	2.0
RECQL	2.0
NRAS	2.0
SNRPC	2.0
SUDS3	2.0
RTN3	2.0
APEX1	2.0
ZNF146	2.0
HSD17B10	2.0
FZR1	2.0
NELFCD	2.0

**Gene Name**	**Fold change relative to normal**
XPO1	2.0
TBL1X	2.0
CCT6A	2.0
FH	2.0
FAM102B	2.0
SEPTIN11	2.0
ABI2	2.0
MRPL3	2.0
DHX9	2.0
SMARCD1	2.0
FOXK2	2.0
RNF187	2.0
LDOC1L	2.0
STIP1	2.0
GTF2A1	2.0
NVL	2.0
TOMM70A	2.0
UBAP2L	2.0
FOXJ3	2.0
ATP9A	2.0
TULP4	2.0
CEP192	2.0
STK35	2.0
BAZ1B	2.0
SLC1A5	2.0
SMAD4	2.0
PTBP1	2.0
KIF1B	2.0
LRP3	2.0
TCP1	2.0
IARS	2.0
MTOR	2.0
SAMD4B	2.0
VDAC3	2.0
GPI	2.0
GTF3C4	1.9
SF3A2	1.9
XRCC5	1.9
PHF14	1.9
KHDRBS1	1.9
ZNF532	1.9
MEGF8	1.9
PSMD2	1.9
DNAJC10	1.9
SIGMAR1	1.9
SMG5	1.9
SLC35B2	1.9
HP1BP3	1.9
SMARCA4	1.9
NDUFB11	1.9
DCAF7	1.9

Gene Name	Fold change relative to normal
HUWE1	1.9
CDKN1B	1.9
ADNP	1.9
KHSRP	1.9
DENND1A	1.9
TRIM33	1.9
ZMYM2	1.9
CDK5RAP2	1.9
TPGS2	1.9
ITM2C	1.9
MTMR4	1.9
AGO1	1.9
SPIN1	1.9
KCTD3	1.9
AUTS2	1.8
GID8	1.8
GSE1	1.8
SMPD4	1.8
STRN4	1.8
CDC5L	1.8
TLK2	1.8
FAM168A	1.8
SMARCC1	1.8
MIS18BP1	1.8
ANKIB1	1.8
HNRNPUL1	1.8
NCOA6	1.7
PHACTR4	1.7

Supplementary Table 4: List of downregulated genes with fold change relative to normal

Gene Name	Fold change relative to normal
C8B	0.002
RP11-77A13.1	0.003
MCEMP1	0.003
KRT14	0.003
FABP4	0.004
CSF3	0.004
RETN	0.005
KRT1	0.008
CHIAP2	0.008
CXCL5	0.008
GPA33	0.008
CYP1A1	0.008
RP5-839B4.8	0.008
MARCO	0.009
SFTPC	0.009
CPB2	0.010
SLC19A3	0.011
HMGCS2	0.011
CYP1A2	0.011
GPIHBP1	0.011
LGI3	0.011
WIF1	0.012
HBG2	0.012
ITLN2	0.012
GPD1	0.012
FOSB	0.012
SLC46A2	0.013
DSG3	0.013
ANKRD1	0.014
C18orf63	0.014
RP11-613D13.8	0.014
TREM1	0.014
AGER	0.014
C10orf99	0.015
FGA	0.015
FOLR3	0.015
GDF10	0.016
HBA2	0.016
OLR1	0.016
CD300LG	0.016
CTB-134H23.3	0.016
CES1	0.017
CXCL2	0.017
PEBP4	0.017
RP11-635O16.2	0.017

Gene Name	Fold change relative to normal
PPBP	0.018
FPR2	0.018
SHH	0.018
CTD-3080P12.3	0.018
RP11-528A4.2	0.018
RASGRF1	0.018
ODAM	0.018
SERPIND1	0.019
ITLN1	0.019
GGTLC1	0.019
RP11-701P16.5	0.019
RP11-238K6.1	0.020
PGC	0.020
VSIG4	0.020
RP11-302F12.1	0.020
TMEM213	0.020
SFTPA1	0.020
SCGB3A1	0.021
ALOX15B	0.021
HCAR2	0.021
AC008268.1	0.021
AP002856.5	0.021
ADRB2	0.021
CYP2B7P	0.021
KRT16	0.021
CLDN18	0.021
PLA2G1B	0.022
RBP4	0.022
SFTPA2	0.022
S100A7	0.022
RP11-357D18.1	0.022
UPK3B	0.022
SPRR2G	0.022
MSLN	0.022
CXCL3	0.022
RP11-164O23.8	0.022
S100A8	0.022
C4BPA	0.023
ECEL1P2	0.023
RSPO2	0.023
STEAP4	0.023
CD5L	0.023
ANXA8L2	0.023
TNNC1	0.024
RPL13AP17	0.024
RP11-325F22.5	0.024
MRC1	0.024

Gene Name	Fold change relative to normal
RN7SL8P	0.024
RXFP2	0.024
XAGE2B	0.024
AC079630.4	0.024
FGG	0.024
AGRP	0.024
GKN2	0.025
LINC01272	0.025
OVCH2	0.025
ACADL	0.025
TCF21	0.025
CTD-2369P2.8	0.025
F11	0.025
VEPH1	0.026
RBPMS-AS1	0.026
HBA1	0.026
NAPSA	0.026
HHIP	0.026
LINC01314	0.026
SFTPD	0.026
RP11-20D14.6	0.027
KRT6A	0.027
LINC00844	0.027
CTD-2589M5.4	0.027
CEACAM8	0.027
CD36	0.027
GSTA3	0.027
RP11-544M22.1	0.027
SIGLEC11	0.027
C16orf89	0.027
RP11-88I21.2	0.027
RP11-141J13.5	0.027
CH17-360D5.2	0.027
KRT16P1	0.028
WNT7A	0.028
SLC11A1	0.028
CAV1	0.028
PF4	0.028
SFTA1P	0.028
RP11-422N16.3	0.028
SCEL	0.029
GSTA2	0.029
PPARG	0.029
AC002066.1	0.029
SLPI	0.029
XXyac-YM21GA2	0.029
EMR1	0.029

**Gene Name**	**Fold change relative to normal**
ADAMTS7P3	0.030
CHIA	0.030
OVCH1	0.030
SCGB1A1	0.030
TPSB2	0.030
TPSAB1	0.030
CNTN6	0.031
ADAMTS9-AS1	0.031
SUSD2	0.031
CCL23	0.031
NALCN-AS1	0.031
HP	0.031
ALOX5AP	0.031
MGC27382	0.031
AQP9	0.031
HBB	0.031
PLA2G4F	0.032
KRT79	0.032
CPAMD8	0.032
ANGPT4	0.032
RP11-476D10.1	0.032
SDPR	0.032
ANXA8	0.032
HYAL1	0.032
RP11-664D7.4	0.032
ADH1B	0.032
ZDHHC19	0.032
SLC34A2	0.033
CFTR	0.033
ALPP	0.033
CTD-2015H3.2	0.033
RP5-1103B4.3	0.033
EPGN	0.033
ABCA8	0.033
CCL20	0.033
WFDC5	0.033
SEC14L3	0.033
EDN3	0.034
SLC22A3	0.034
LEPREL1	0.034
AQP3	0.034
RP11-352D13.6	0.034
NTN4	0.034
RP11-357P18.2	0.034
RP11-133L19.3	0.034
MUC15	0.034
APOH	0.035

Gene Name	Fold change relative to normal
HBM	0.035
SYNC	0.035
SEC14L6	0.035
AC079630.2	0.035
PIGR	0.035
TRHDE	0.035
FGFBP2	0.035
ATOH8	0.035
CAMP	0.035
FCGR3B	0.035
SELE	0.035
ATP13A4	0.035
RP11-251M1.1	0.036
RSPO1	0.036
SGMS2	0.036
MT1JP	0.036
TNNI2	0.036
RP11-403A3.3	0.036
FPR1	0.036
SCARA5	0.036
SPRR3	0.036
MYO16-AS1	0.036
FCER1A	0.037
ALPPL2	0.037
CCDC141	0.037
RP11-789C1.1	0.037
CYP4B1	0.037
AC007970.1	0.037
LINC00656	0.037
CD300C	0.037
AQP4	0.037
AREG	0.037
GYPE	0.037
CYP4Z2P	0.037
FIGF	0.038
SFTPB	0.038
BTNL9	0.038
LRRK2	0.038
FOLR1	0.038
DLC1	0.038
CAV2	0.038
INMT	0.038
ADTRP	0.038
C2orf91	0.038
NTF4	0.038
AQP7	0.038
GPRC5A	0.038

**Gene Name**	**Fold change relative to normal**
AGBL1	0.038
WFDC12	0.038
DSG1	0.038
KRT6C	0.039
CHRNA2	0.039
TMPRSS2	0.039
AC092071.1	0.039
S100A9	0.039
KCNJ15	0.039
PTPRQ	0.039
CFD	0.039
KRT16P2	0.039
CLCA2	0.039
RP11-85G21.3	0.039
BTNL8	0.039
CHI3L2	0.039
WNT3A	0.039
ADRA1A	0.040
HAS1	0.040
TRIM71	0.040
S100A12	0.040
RP1-251M9.3	0.040
PRX	0.040
FCN3	0.040
CLEC3B	0.040
HSPB3	0.040
NPR1	0.040
PLA2G4E	0.040
RP1-35C21.1	0.040
EGR1	0.040
RP3-460G2.2	0.041
CLIC3	0.041
LINC01146	0.041
LINC00968	0.041
HCAR3	0.041
C2orf71	0.041
RP11-213H15.1	0.041
KRT24	0.041
RP11-720L2.4	0.041
FGF10	0.041
DMBT1	0.042
AKR1C3	0.042
ADH1A	0.042
ANKRD29	0.042
FGFR4	0.042
CLEC12A	0.042
IL1A	0.042

Gene Name	Fold change relative to normal
AWAT2	0.042
SCGB3A2	0.042
IL1RL1	0.042
FAM107A	0.042
CYS1	0.042
SOCS3	0.042
ALAS2	0.042
C11orf96	0.043
LAMP3	0.043
DPEP2	0.043
SLC1A1	0.043
CSF3R	0.043
SIRPB1	0.043
AMZ1	0.043
IL6	0.043
RP11-286H15.1	0.043
DEFA3	0.043
ALDH2	0.043
RP11-79H23.3	0.044
LILRB3	0.044
TDRD10	0.044
ST8SIA6	0.044
PRAM1	0.044
ANXA3	0.044
FAM150B	0.044
AC093110.3	0.045
CTSH	0.045
ZMYND15	0.045
MATN3	0.045
MS4A2	0.045
CH17-360D5.3	0.045
ZFP36	0.045
HHIP-AS1	0.045
SLC22A31	0.045
GADD45B	0.045
SLC39A8	0.045
AC090616.2	0.045
PLBD1	0.045
TBX2-AS1	0.046
GALNT5	0.046
HPR	0.046
CTD-3107M8.2	0.046
FMO2	0.046
MSR1	0.046
CLEC1B	0.046
SYT15	0.046
AC006273.5	0.046

**Gene Name**	**Fold change relative to normal**
PLA2G2A	0.046
CCIN	0.046
OR52K3P	0.046
VMO1	0.046
MT1A	0.047
MYOC	0.047
CTD-2135D7.5	0.047
SCTR	0.047
ADAMTS8	0.047
EDNRB	0.047
TMEM139	0.047
HPGDS	0.047
CTB-167B5.2	0.047
TMEM52B	0.047
SLC6A4	0.047
CXCR2	0.047
SFTA2	0.048
IRX6	0.048
TMEM150B	0.048
KNDC1	0.048
RP11-462G2.1	0.048
CPA3	0.048
FOS	0.048
PRG4	0.048
PTCRA	0.048
RP11-35J10.6	0.048
DCSTAMP	0.048
SERPINA1	0.048
SPINK6	0.048
RBMS3-AS3	0.048
GP9	0.049
CH25H	0.049
GPR116	0.049
ANPEP	0.049
RP11-359N11.1	0.049
C8A	0.049
SYNDIG1L	0.049
BNC1	0.049
RXFP1	0.049
C1orf162	0.049
EGR2	0.049
RP11-293P20.2	0.050
FGR	0.050
ALPL	0.050
GGTLC2	0.050
RASGRP4	0.050
CXCR1	0.050

Gene Name	Fold change relative to normal
MYRF	0.050
HOPX	0.050
CTC-573N18.1	0.050
CASP14	0.050
ABCC6	0.050
NXF3	0.051
GPX3	0.051
AGTR2	0.051
SDR16C5	0.051
RP11-125O18.1	0.051
ZNF812	0.051
FHL5	0.051
GSTM5	0.051
C1orf116	0.051
LRP2	0.051
MMP19	0.051
HK3	0.052
IYD	0.052
AMICA1	0.052
AC013264.2	0.052
ITIH5	0.052
NDNF	0.052
AC064834.3	0.052
LRRN4	0.052
NLRC4	0.052
RP11-434D9.1	0.052
CCND2-AS1	0.052
SIGLEC5	0.052
HPGD	0.053
DOK2	0.053
RP11-470M17.2	0.053
LINC00551	0.053
PTGDS	0.053
CRTAC1	0.053
NCF2	0.053
CSRNP1	0.054
RP11-354E11.2	0.054
HNF1B	0.054
FBP1	0.054
TMEM236	0.054
CDH19	0.054
FOSL1	0.054
AC098828.3	0.054
TNXA	0.054
DIO3OS	0.054
CATSPERD	0.054
VGLL1	0.054

Gene Name	Fold change relative to normal
RP11-246K15.1	0.054
COLEC12	0.054
ROS1	0.054
HBD	0.054
ZBED2	0.054
CCL17	0.054
SPRR1B	0.055
AC011899.9	0.055
GPR17	0.055
GPX1P1	0.055
PTPLA	0.055
SHE	0.055
GATA6	0.055
TEK	0.055
REG1A	0.056
ICAM1	0.056
CXCL16	0.056
SLC4A1	0.056
RP11-805I24.3	0.056
TPSD1	0.056
KHDRBS2	0.056
OR2S1P	0.056
AOC3	0.056
HSD17B6	0.056
ARHGAP31	0.056
CD52	0.056
RP1-186E20.1	0.056
DUSP1	0.056
RNASE4	0.057
SPI1	0.057
PLAC9	0.057
TGFBR2	0.057
STX11	0.057
PCDH15	0.057
CLEC12B	0.057
CYP1B1-AS1	0.057
S100A4	0.057
FGB	0.057
SCN7A	0.057
HLA-DRB5	0.057
PNMT	0.057
SIGLEC1	0.057
ADAMTSL4	0.057
GPR126	0.057
NFKBIZ	0.058
KANK3	0.058
RP11-371A19.2	0.058

**Gene Name**	**Fold change relative to normal**
CD33	0.058
RP3-462E2.3	0.058
DRAM1	0.058
RP11-1024P17.1	0.058
FGD5	0.058
CD163	0.058
EREG	0.058
LINC01108	0.058
SLC6A14	0.058
CTSLP8	0.059
SERPINB7	0.059
HBG1	0.059
KLF2	0.059
SLC5A9	0.059
RP11-206P5.2	0.059
EPAS1	0.059
RP11-750H9.5	0.059
SIGLEC17P	0.059
GSTA1	0.060
RP1-240B8.3	0.060
RRAD	0.060
SULT1A2	0.060
MALL	0.060
TMEM100	0.060
PADI4	0.060
ALDH3B1	0.060
SH3RF2	0.060
RP11-287F9.2	0.060
SPOCD1	0.060
AQP1	0.060
SELENBP1	0.061
CMTM5	0.061
AC079467.1	0.061
BMP3	0.061
GATA1	0.061
HLA-DRB1	0.061
OSCAR	0.062
LY86	0.062
WWC2-AS2	0.062
RP11-76E17.3	0.062
NLRP12	0.062
TACSTD2	0.062
RP11-1008C21.1	0.062
RP1-78O14.1	0.062
CLIC5	0.062
CTD-2306A12.1	0.063
FLRT3	0.063

**Gene Name**	**Fold change relative to normal**
RP11-274B18.2	0.063
ORM1	0.063
TMPRSS11D	0.063
ICAM5	0.063
ZNF385B	0.063
CA3	0.063
GLIPR2	0.063
RGCC	0.063
CA2	0.063
C1QA	0.063
AP001626.1	0.064
SLC6A13	0.064
GATA6-AS1	0.064
GYPC	0.064
CATSPER1	0.064
RP11-101E5.1	0.064
MFAP4	0.064
EDN1	0.064
ANGPT1	0.064
AC019117.1	0.064
ADAMTS9-AS2	0.065
CTA-363E6.2	0.065
FAM189A2	0.065
IL6R	0.065
C1QB	0.065
RP11-526F3.1	0.065
TMEM92	0.065
DPYS	0.065
KRT16P3	0.065
TLR2	0.065
CACNA2D2	0.065
LIPH	0.065
CYBRD1	0.065
LYVE1	0.065
RP11-35J10.7	0.065
ADH7	0.065
ITGAM	0.066
HBEGF	0.066
ADRB1	0.066
MIR223	0.066
RP11-815M8.1	0.066
EMR3	0.066
CSF2	0.066
CPM	0.066
C8orf34	0.066
THBD	0.066
GUCA2A	0.067

**Gene Name**	**Fold change relative to normal**
FFAR4	0.067
MNDA	0.067
LINC01186	0.067
PDLIM2	0.067
RP11-317J10.2	0.067
PLA2G12B	0.067
AC007182.6	0.067
BHLHE41	0.067
LINC00694	0.067
SPATA20P1	0.067
LINC00842	0.067
AC093787.1	0.067
CYP2S1	0.067
MICA	0.068
RP11-720L2.3	0.068
PROS1	0.068
TMEM132C	0.068
WWC2	0.068
RP11-452C13.1	0.068
RND1	0.068
SIGLEC22P	0.068
MAP1LC3C	0.068
KRT4	0.068
TNNT3	0.069
RP11-655M14.4	0.069
AHNAK	0.069
MFAP3L	0.069
YAP1	0.069
PDE1B	0.069
SLC7A7	0.069
CAPN8	0.069
SLC39A12	0.069
TSKS	0.069
SEMA3B	0.069
CXCL1	0.069
MYZAP	0.070
RP11-336K24.5	0.070
RP5-826L7.1	0.070
FGFBP1	0.070
CTD-2078B5.2	0.070
PTAFR	0.070
SLC16A5	0.070
ATP1A2	0.070
FCN1	0.070
C11orf97	0.070
ACP5	0.071
C1QTNF7	0.071

Gene Name	Fold change relative to normal
FENDRR	0.071
SPOCK2	0.071
AC013275.2	0.071
RP11-789C1.2	0.071
FGFR2	0.071
TLR8	0.071
C19orf33	0.071
APOC1	0.071
ZBTB16	0.071
GCSAML	0.071
MS4A7	0.071
ABCC13	0.071
CD97	0.071
SLCO2B1	0.071
PAPSS2	0.071
CYP4F32P	0.072
CYP17A1	0.072
TRPV2	0.072
PTH1R	0.072
ASPA	0.072
HSPB6	0.072
CHRDL1	0.072
MAP3K8	0.072
C15orf59	0.072
PRELID2P1	0.072
RP11-1008C21.2	0.073
LDHD	0.073
NEDD9	0.073
DPP4	0.073
SERPINA9	0.073
BCL2A1	0.073
ID1	0.074
LIMS2	0.074
CTD-3247F14.2	0.074
GCNT4	0.074
LINC00211	0.074
SOD3	0.074
APOBR	0.074
RP11-1149M10.2	0.074
CYBB	0.074
FCGR3A	0.074
HIGD1B	0.074
CTD-2530N21.5	0.074
ADCY8	0.074
RP11-395E19.6	0.074
DKK1	0.074
RP11-335L23.5	0.074

Gene Name	Fold change relative to normal
C5AR1	0.074
TYROBP	0.075
RP11-365O16.3	0.075
MIR27A	0.075
OSTN	0.075
IL34	0.075
SOSTDC1	0.075
ASZ1	0.075
DCN	0.075
TEKT4	0.075
HSPB8	0.076
C10orf115	0.076
PI3	0.076
RP11-272L13.4	0.076
RP11-44D19.1	0.076
ABCA3	0.076
ASPG	0.076
CH17-360D5.1	0.077
CTD-3032H12.1	0.077
VIPR1	0.077
LINC01166	0.077
CST6	0.077
ABCB1	0.077
PSCA	0.077
COL6A5	0.077
HLF	0.077
AGTR1	0.077
AC006129.1	0.077
DNAH17-AS1	0.078
ABI3BP	0.078
RP11-481H12.1	0.078
S100A3	0.078
RP11-64C12.8	0.078
LYPD2	0.078
LIF	0.078
PTPN13	0.078
LDB2	0.078
SIRPD	0.078
SIGLEC6	0.078
RP11-616M22.5	0.078
MLPH	0.078
LRG1	0.079
ZNF365	0.079
ADAMTSL3	0.079
TREM2	0.079
A2M	0.079
TIMP3	0.079

**Gene Name**	**Fold change relative to normal**
CPED1	0.079
RP1-229K20.5	0.079
MS4A14	0.079
FBLN5	0.079
RP11-356N1.2	0.079
FILIP1	0.079
AXL	0.079
STARD13-AS	0.079
ADAMTS1	0.079
RP11-280O1.2	0.079
GGTA1P	0.079
TBX2	0.080
RBP2	0.080
CTD-2003C8.2	0.080
SELPLG	0.080
PGAM1P5	0.080
NFE2	0.080
S1PR4	0.080
S100A10	0.080
FAM46B	0.080
VIM	0.081
FMO5	0.081
SLURP1	0.081
RP11-236L14.2	0.081
RP11-16K12.1	0.081
CLEC14A	0.081
RP11-95I16.6	0.081
NFAM1	0.081
CCDC69	0.081
SIRPB2	0.081
MT1M	0.081
PTPRB	0.081
GREM2	0.082
TBX4	0.082
EMCN	0.082
SRPX	0.082
RNASE1	0.082
PROSER2	0.082
NCF1	0.082
RP11-806O11.1	0.082
RBPMS	0.082
KRT13	0.082
C1QC	0.082
PSAPL1	0.083
SCN2B	0.083
SNTN	0.083
KLK7	0.083

Gene Name	Fold change relative to normal
EHD2	0.083
SPN	0.083
MALRD1	0.083
LRRC36	0.083
KRT27	0.084
CEBPA	0.084
HEMGN	0.084
AC016745.3	0.084
PHOSPHO1	0.084
C12orf54	0.084
EPDR1	0.084
LYZ	0.084
LINC00961	0.084
CD164L2	0.084
EPHA2	0.084
RP4-575N6.5	0.084
CCDC68	0.084
S1PR1	0.084
MS4A4A	0.085
AC079325.6	0.085
RP11-243M5.1	0.085
SLC15A3	0.085
MITF	0.085
SHROOM4	0.085
LINC00163	0.085
LRRC18	0.085
ANGPTL1	0.085
CTD-3035K23.7	0.085
FCGR1C	0.085
WDR38	0.085
ALOX5	0.085
EFEMP1	0.086
TUBB1	0.086
BTK	0.086
DEFA4	0.086
RP11-82L18.4	0.086
ANKRD2	0.086
SIGLEC7	0.086
DUOXA1	0.087
P2RX1	0.087
CMTM2	0.087
DAW1	0.087
VNN1	0.087
PDZD2	0.087
VWA3A	0.087
CD300LF	0.087
AC060834.2	0.087

Gene Name	Fold change relative to normal
SPARCL1	0.087
ODF3L1	0.087
CES1P1	0.087
SH3GL3	0.087
ZC3H12A	0.087
C15orf52	0.087
EGFL7	0.088
ARG1	0.088
PILRA	0.088
FAM162B	0.088
RN7SKP18	0.088
PDPN	0.088
NOSTRIN	0.088
PDE4C	0.088
UNC13D	0.088
P2RY12	0.088
JAM2	0.088
WFDC1	0.088
COL4A3	0.089
CFI	0.089
EVI2B	0.089
WTIP	0.089
RP11-276H7.3	0.089
FLVCR2	0.089
RAPGEF3	0.089
CTSG	0.089
SPTLC3	0.089
C7	0.089
CLEC1A	0.089
NNMT	0.090
MMP28	0.090
MAOA	0.090
SYT8	0.090
ACVRL1	0.090
RP11-622C24.2	0.090
CRISP2	0.090
TM4SF4	0.090
LINC00113	0.090
CD44	0.090
SIGLEC9	0.090
AC104654.2	0.090
IL1RN	0.090
CD1C	0.090
RP11-64B16.2	0.090
SPATS1	0.090
MAMDC2	0.090
RASGRP2	0.090

Gene Name	Fold change relative to normal
TMEM204	0.090
AC017060.1	0.090
AOC4P	0.091
AC105053.3	0.091
SLC4A4	0.091
PDZK1IP1	0.091
LINC00689	0.091
RP3-425P12.2	0.091
NPR3	0.091
WISP2	0.091
CYP27A1	0.091
CLEC7A	0.091
LINC01160	0.091
FOXF1	0.091
SOCS2	0.091
EVPLL	0.091
RP11-403A21.1	0.092
STAC	0.092
VNN3	0.092
IL33	0.092
BMP4	0.092
PCAT19	0.092
IL1R1	0.092
HSPA12B	0.092
JUNB	0.092
RBMS3	0.092
SPRR2C	0.092
DTX4	0.092
CD55	0.092
FSTL3	0.093
CLEC4E	0.093
ITGB6	0.093
RP11-144A16.8	0.093
CD1E	0.093
LRRC52	0.093
C10orf128	0.093
CYP4F29P	0.093
CYP2A6	0.093
ITGB2	0.093
SOWAHD	0.093
KLK5	0.093
MFSD7	0.093
TNS1	0.094
CASP12	0.094
ATP2C2	0.094
DAB2	0.094
SLC10A2	0.094

**Gene Name**	**Fold change relative to normal**
CYR61	0.094
GPR65	0.094
BST1	0.094
PECAM1	0.094
C3	0.094
LINC00504	0.094
AHCYL2	0.094
RP11-114G11.5	0.094
RP11-213O5.4	0.094
GPR133	0.094
ROBO4	0.094
RP11-312J18.6	0.094
ABCG2	0.094
CREB3L1	0.095
CATIP	0.095
C3orf55	0.095
TNFSF12	0.095
MGC32805	0.095
CTB-138E5.1	0.095
LCNL1	0.095
PTRF	0.095
KLK8	0.095
FABP3	0.095
RASSF9	0.095
REM1	0.096
F3	0.096
NCF1C	0.096
DUOX1	0.096
TREML1	0.096
RP11-470L19.2	0.096
CGNL1	0.096
RP3-369A17.4	0.096
RP11-320N7.2	0.096
EGF	0.097
BNIPL	0.097
TBX5-AS1	0.097
FCGR2A	0.097
BCAS1	0.097
IRX3	0.097
MIR221	0.097
AVPI1	0.097
PTGS2	0.097
LINC01081	0.097
MGAM	0.097
CDH16	0.097
CLEC4D	0.097
FXYD1	0.098

Gene Name	Fold change relative to normal
CTSW	0.098
FGFR3	0.098
TFPI	0.098
TIE1	0.098
CHP2	0.098
LAMA3	0.098
SLC47A1	0.098
LMOD3	0.098
IL7R	0.099
B3GNT7	0.099
GNG11	0.099
RP11-625L16.4	0.099
NPY4R	0.099
SYT15	0.099
FAM110D	0.099
CR1	0.099
RP11-212I21.2	0.099
PI16	0.099
SCIMP	0.099
RP11-134G8.8	0.099
KPNA7	0.099
C20orf85	0.099
PRODH	0.099
UPK2	0.099
LATS2	0.099
VENTX	0.099
RP11-327J17.9	0.099
SLC24A4	0.099
LIPN	0.099
TEPP	0.099
CCL2	0.099
RP11-95I16.2	0.099
MYL3	0.099
CEBPE	0.100
CAPNS2	0.100
GGT2	0.100
CELA3B	0.100
RP11-800A3.4	0.100
RP11-838N2.4	0.100
COX7A1	0.100
TGM4	0.100
CISH	0.100
CTSS	0.101
XXyac-YM21GA2	0.101
RP5-899E9.1	0.101
RNF128	0.101
OIT3	0.101

**Gene Name**	**Fold change relative to normal**
GPR87	0.101
AC003075.4	0.101
RP1	0.101
LEPREL1-AS1	0.101
ARRB1	0.101
RAMP3	0.101
RP11-676J12.7	0.101
RP11-403B2.7	0.101
Z84812.4	0.101
CYGB	0.101
IL13RA2	0.101
RP11-320G24.1	0.102
ARHGAP6	0.102
AC009238.8	0.102
ENPP3	0.102
RP11-44B19.1	0.102
SOX17	0.102
MYL9	0.102
HSPA8P20	0.102
SPINK7	0.102
NAPSB	0.102
TNFAIP8L2	0.102
SLC51B	0.102
ADCY7	0.103
CA4	0.103
ARHGAP40	0.103
ARHGEF15	0.103
NR3C2	0.103
TRPC6	0.103
GNG7	0.103
RP11-403A3.2	0.103
RP11-512N21.3	0.103
NCF1B	0.103
ERP27	0.103
RRAS	0.103
AP000438.2	0.103
RP11-63P12.6	0.103
CD93	0.103
GAPT	0.103
LINC01169	0.103
HKDC1	0.104
SLCO2A1	0.104
HHLA2	0.104
AP001189.4	0.104
EVA1A	0.104
MGAT3	0.104
LINC01165	0.104

**Gene Name**	**Fold change relative to normal**
GHR	0.105
TAL1	0.105
RN7SKP51	0.105
AC108868.5	0.105
EMP1	0.105
LINC01094	0.105
WDFY4	0.105
ESAM	0.105
CXCL17	0.105
RP13-580F15.2	0.105
SGK1	0.105
GMFG	0.105
TMEM150A	0.105
CAT	0.105
RP11-473M20.9	0.105
GRTP1-AS1	0.105
MYCT1	0.105
LINC00443	0.106
PAQR5	0.106
MYO1F	0.106
SEMA3G	0.106
DKK2	0.106
PPP1R42	0.106
C10orf54	0.106
LCN6	0.106
GPC5	0.106
LLNLR-470E3.1	0.106
CTD-2562J17.7	0.106
GIMAP8	0.106
RMDN2	0.106
TDRD9	0.106
VIM-AS1	0.107
FAT4	0.107
S100P	0.107
THEMIS2	0.107
LRRC32	0.107
KRT73	0.107
KMO	0.107
PTGER2	0.107
FCER1G	0.107
PLXDC2	0.107
BLVRB	0.107
GCSAML-AS1	0.107
LRRC25	0.107
ITGAX	0.107
SLC37A2	0.108
LIPA	0.108

Gene Name	Fold change relative to normal
NTRK3	0.108
TNFSF13	0.108
MPP7	0.108
KLF15	0.108
LDLR	0.108
PHLDA3	0.108
RIN3	0.108
METRN	0.108
LPAL2	0.108
AC004988.1	0.108
APOE	0.108
LRP1	0.109
SHC3	0.109
SERPING1	0.109
CAV3	0.109
RP11-1223D19.1	0.109
RPL23AP35	0.109
RP11-56A10.1	0.109
ANG	0.109
CTSD	0.109
PTPN21	0.109
AFF3	0.109
SGCA	0.109
CST5	0.109
HLA-DMA	0.109
RP11-408O19.5	0.109
PRSS57	0.109
RGN	0.110
CLDN1	0.110
TBC1D2	0.110
RP11-283G6.4	0.110
ACE2	0.110
PCDH20	0.110
C10orf107	0.110
PIH1D3	0.110
CYMP	0.110
AP002954.4	0.110
LINC01267	0.110
RP11-589N15.2	0.110
KCNA10	0.110
TGM2	0.110
SQRDL	0.111
KLB	0.111
RP11-548P2.2	0.111
P2RY13	0.111
PPP1R14A	0.111
G0S2	0.111

**Gene Name**	**Fold change relative to normal**
AC019117.2	0.111
ELF5	0.111
AC123886.2	0.112
CD69	0.112
FCGR1A	0.112
AADAC	0.112
SNTB1	0.112
HNMT	0.112
RP11-203P23.2	0.112
RP11-121A8.1	0.112
GLRX	0.112
SMG8	0.112
GATA5	0.112
CYP4A11	0.112
CEBPD	0.112
RP11-20J15.3	0.112
CTD-2626G11.2	0.113
TLR5	0.113
SEMA3C	0.113
AC116035.1	0.113
NUPR1	0.113
BX842568.1	0.113
KLF8	0.113
CD74	0.113
CEACAM6	0.113
CTD-2515H24.2	0.113
TSC22D3	0.113
CTD-2531D15.4	0.113
NCF4	0.113
PALMD	0.114
LAPTM5	0.114
EMR4P	0.114
GPR97	0.114
RP11-567J20.3	0.114
TGFB1	0.114
RP11-352D13.5	0.114
CTA-85E5.10	0.114
SH2D3C	0.114
AHR	0.114
FCGR1B	0.114
MYBPC3	0.114
CASS4	0.114
SIGLEC18P	0.115
IL4R	0.115
RP11-472G21.2	0.115
IL1B	0.115
WARSP1	0.115

Gene Name	Fold change relative to normal
AJUBA	0.115
P2RY2	0.115
KLF9	0.115
AC006273.4	0.115
RN7SL475P	0.115
PMP22	0.115
APOBEC3A	0.115
ATP13A4-AS1	0.115
HRCT1	0.115
IL18R1	0.115
S100A14	0.115
Metazoa_SRP	0.116
IL18	0.116
SLC16A11	0.116
RP4-666F24.3	0.116
GNGT2	0.116
AC109642.1	0.116
RTKN2	0.116
FAM124B	0.116
RP11-440I14.3	0.116
MYH2	0.116
SIGLEC16	0.116
RP11-1C1.7	0.116
RP1-151F17.2	0.116
HCK	0.116
CX3CR1	0.116
CX3CL1	0.116
CR2	0.116
LINC00890	0.116
RNU6ATAC18P	0.117
DENND3	0.117
FGD2	0.117
RP11-475O23.2	0.117
LGALS3	0.117
TRAV5	0.117
AC007381.3	0.117
ACOXL	0.118
RBMY1KP	0.118
DHRS9	0.118
FAM166B	0.118
GPR123	0.118
RP11-626H12.1	0.118
GABRE	0.118
CARD16	0.118
AOX1	0.118
ST6GALNAC1	0.118
MUC1	0.119

**Gene Name**	**Fold change relative to normal**
CTSC	0.119
SIRPA	0.119
TMEM119	0.119
CD37	0.119
NR4A1	0.119
C1orf186	0.119
CD302	0.119
KIAA1462	0.120
CLIP4	0.120
RP11-155N3.4	0.120
EMP2	0.120
AQP10	0.120
CORIN	0.120
KLRB1	0.120
ADPRH	0.120
OR5BA1P	0.120
ZNF727P	0.120
AC092652.1	0.120
PRELP	0.121
TRGJP2	0.121
PER1	0.121
RP11-93B14.10	0.121
AC083949.1	0.121
RP11-820L6.1	0.121
AC016735.2	0.121
ACSL5	0.121
SLC25A47P1	0.121
ABCC6P2	0.121
RP11-120M18.5	0.121
HMOX1	0.121
RP11-720N19.1	0.121
DLEC1	0.121
STOM	0.121
DAPK2	0.121
SLC6A12	0.121
CTC-436P18.3	0.121
RP11-350G8.5	0.121
ORM2	0.121
ASAH1	0.121
C3AR1	0.121
TBXAS1	0.121
ENG	0.122
RP11-277P12.10	0.122
C5AR2	0.122
ACKR4	0.122
RP11-732A21.3	0.122
PEG3	0.122

Gene Name	Fold change relative to normal
SIRPAP1	0.122
LAMB3	0.122
RP11-673E1.3	0.122
IL1R2	0.122
AC011516.2	0.123
TNIP3	0.123
ACOX2	0.123
RP11-800A3.7	0.123
RP11-44F14.1	0.123
LTA4H	0.123
RP11-641D5.2	0.123
PDE6G	0.123
KIF1C	0.123
TLR4	0.123
C6orf229	0.123
COL13A1	0.123
CNN2	0.123
OSMR	0.123
PKDCC	0.123
LAIR1	0.124
TCTEX1D4	0.124
RP11-671P2.1	0.124
SIDT2	0.124
DNASE2B	0.124
ABCC3	0.124
AC018647.3	0.124
TNXB	0.124
GGT1	0.124
MLC1	0.124
ALS2CL	0.124
CALCRL	0.125
STARD8	0.125
CD22	0.125
BHLHE40	0.125
BIN2	0.125
GLP2R	0.125
RP4-530I15.10	0.125
PRKCQ	0.125
PCP4L1	0.125
APBB1IP	0.125
MSRB3	0.125
TMBIM1	0.125
RP11-467L13.5	0.125
NPC2	0.125
RP11-1100L3.8	0.125
LINC01010	0.125
AC073130.1	0.125

**Gene Name**	**Fold change relative to normal**
RP11-164J13.1	0.126
GIMAP1	0.126
AKAP13	0.126
ATP13A5	0.126
RP11-446E9.2	0.126
HGF	0.126
APOC1P1	0.126
RP11-588K22.2	0.126
SNORA48	0.126
AP001434.2	0.126
RP11-370I10.2	0.126
GPER1	0.126
RP11-259K15.2	0.126
KCNJ8	0.127
TFEC	0.127
RP1-28O10.1	0.127
RPSAP47	0.127
RP11-373D23.3	0.127
RP11-736K20.5	0.127
LMTK3	0.127
LPL	0.127
TNFRSF10B	0.127
CDA	0.127
SMAD6	0.128
RP13-577H12.2	0.128
RP11-627G18.1	0.128
CARD6	0.128
KLRF1	0.128
OSGIN1	0.128
FERMT3	0.128
NEXN	0.128
ARHGAP18	0.128
IFITM2	0.128
ALDH1A1	0.128
FIBIN	0.128
BMP2	0.128
SPATC1	0.128
CTC-441N14.2	0.128
PDE4A	0.128
C19orf35	0.128
RP11-7F17.3	0.128
MROH9	0.129
GAS2L1	0.129
FAM20A	0.129
C1orf200	0.129
TMEM125	0.129
TRIB1	0.129

**Gene Name**	**Fold change relative to normal**
RP11-823E8.3	0.129
CSF2RB	0.129
CD53	0.129
CAPN11	0.129
SRGN	0.129
SECISBP2L	0.130
SLC2A9	0.130
ECSCR	0.130
OSMR-AS1	0.130
RP11-82L18.2	0.130
TPRG1	0.130
LIMCH1	0.130
SLC43A2	0.130
SASH3	0.130
DENND2C	0.130
LILRA5	0.130
ADAM29	0.130
CFP	0.130
RP11-93B14.9	0.131
CA12	0.131
VSIG2	0.131
RP11-70C1.1	0.131
ZYX	0.131
CCRL2	0.131
NIM1K	0.131
IGJ	0.131
AC007277.3	0.131
MS4A3	0.131
C8orf4	0.131
CETP	0.131
SH2D4B	0.131
GPSM3	0.131
CRIM1	0.131
RP11-359M6.1	0.131
ABCA6	0.132
GPR110	0.132
FTL	0.132
AC106788.1	0.132
MRGPRF	0.132
PRRG1	0.132
MYH11	0.132
EFEMP2	0.132
LINC00892	0.132
SULT1A1	0.132
ACE	0.132
KRT40	0.132
AC092569.2	0.132

**Gene Name**	**Fold change relative to normal**
SAMD4A	0.132
GPNMB	0.132
TLR7	0.132
RP11-85G21.2	0.133
CTB-43E15.2	0.133
SLC1A7	0.133
MIR24-2	0.133
ITPR1	0.133
MYO1G	0.133
LTBP4	0.133
USP30-AS1	0.133
RP11-432J24.5	0.133
RP4-575N6.4	0.133
PTGDR	0.133
RP13-213K19.1	0.133
TPK1	0.133
EVI2A	0.134
KLF4	0.134
RP11-300J18.1	0.134
MDFIC	0.134
SH2D1B	0.134
RNF144B	0.134
EMP3	0.134
RP11-202D1.3	0.134
SLC15A2	0.134
GSDMC	0.135
EPPIN	0.135
DEFB4A	0.135
C11orf21	0.135
CTD-2245E15.3	0.135
RP11-381N20.1	0.135
CD4	0.135
SLA	0.135
MMRN2	0.135
AC144831.1	0.135
LINC00702	0.135
RAB31	0.135
RENBP	0.135
KLHL6	0.135
OSR1	0.136
OMG	0.136
FAM89A	0.136
RPL31P40	0.136
LPXN	0.136
SHISA3	0.136
TENC1	0.136
TMEM71	0.136

Gene Name	Fold change relative to normal
RP11-309N17.4	0.136
ANKDD1B	0.136
RP11-159H3.1	0.136
SEMA3E	0.136
ZEB2	0.136
MPZL2	0.136
P2RX2	0.136
SLC16A4	0.136
TRPC2	0.136
RP1-120G22.11	0.137
CTD-3023J11.2	0.137
OGN	0.137
MTUS1	0.137
ABLIM3	0.137
RP11-432J24.2	0.137
CEACAM3	0.137
RP11-333A23.4	0.137
RP11-443B7.3	0.137
FAM3D	0.137
RP4-576H24.2	0.137
RGS18	0.137
ZNF300P1	0.137
GUCY1A3	0.137
SECTM1	0.138
COL4A4	0.138
RP11-459E5.1	0.138
OLFML1	0.138
RP1-29C18.9	0.138
SYNPO	0.138
LGALS4	0.138
PARM1	0.139
RP11-626A5.2	0.139
MRC2	0.139
ITPKC	0.139
TRBV28	0.139
SYDE1	0.139
TRAV18	0.139
FUOM	0.139
ADCY4	0.139
CITED2	0.139
TP63	0.139
CTRB1	0.139
CELF2-AS2	0.139
CCDC42B	0.139
HVCN1	0.139
PRKG1	0.140
AC083900.1	0.140

Gene Name	Fold change relative to normal
EDAR	0.140
VSTM4	0.140
GJA5	0.140
TMEM243	0.140
RP11-864I4.3	0.140
PCOLCE2	0.140
ACSL1	0.140
AGPAT9	0.140
AC147651.4	0.141
KRT87P	0.141
CTGF	0.141
TSPO	0.141
RP11-156K13.1	0.141
CD86	0.141
RAB11FIP1	0.141
ARAP2	0.141
UBTD1	0.141
ABCC9	0.141
RP11-264B14.1	0.141
DHRS3	0.142
VNN2	0.142
ITGA8	0.142
HNRNPA1P33	0.142
C20orf166-AS1	0.142
SVEP1	0.142
DNAAF1	0.142
IFFO1	0.142
AKR1C1	0.142
TSPAN32	0.142
UNC45B	0.142
ADAMTSL4-AS1	0.142
FRMD4B	0.142
RP11-541N10.3	0.142
LINC00982	0.143
FAH	0.143
CLEC2B	0.143
CDC42EP1	0.143
TRIM61	0.143
CDH5	0.143
HS3ST2	0.143
RP3-332B22.1	0.143
RCVRN	0.143
ADAMTSL5	0.143
CA1	0.143
SEMA4A	0.143
OR6K3	0.143
CAPN3	0.143

**Gene Name**	**Fold change relative to normal**
PLCD3	0.143
C5orf38	0.144
PTPRE	0.145
ITGAL	0.145
ALDH3A1	0.145
THSD1	0.145
TCL1A	0.145
AC008592.5	0.145
IL20RA	0.145
LRRC2	0.145
FKBP5	0.146
RAB27B	0.146
GPRIN2	0.146
FAM83E	0.146
DAAM2	0.146
CELF2	0.146
RILPL2	0.146
ZCCHC24	0.146
VWF	0.146
DNAJC5B	0.146
SLED1	0.147
AJ239322.1	0.147
TNFRSF10D	0.147
ELL2	0.147
PLCB2	0.147
UPB1	0.147
KRBOX1	0.147
ATP10A	0.147
LRRN4CL	0.147
CCR1	0.147
FCGRT	0.147
CASP10	0.148
CSF1R	0.148
CAPG	0.148
GJA4	0.148
CXCL8	0.148
FAM81B	0.148
NCKAP5	0.148
ZFPM2	0.148
RHOB	0.148
Y_RNA	0.148
CTD-3203P2.3	0.148
LRRC71	0.148
NHLRC4	0.148
ARHGDIB	0.148
FOXF2	0.149
LINC00472	0.149

Gene Name	Fold change relative to normal
IL1RAP	0.149
PRSS30P	0.149
FTLP3	0.149
RP4-555D20.2	0.149
ART4	0.149
MIR23A	0.149
RP11-793H13.3	0.149
RASL12	0.149
RP11-340F14.6	0.149
TMEM130	0.149
SLC31A2	0.149
RP11-597D13.9	0.149
ATP8B5P	0.149
ZEB2-AS1	0.149
TMC8	0.150
RP5-887A10.1	0.150
DOCK2	0.150
RP5-899B16.1	0.150
PGM5	0.150
GBGT1	0.150
BCAT1	0.150
MST1L	0.150
PYCARD	0.150
CD1B	0.150
IGFBP4	0.150
RP11-800A3.2	0.150
IL18RAP	0.151
LSP1	0.151
ITGA5	0.151
NKAPL	0.151
CRYAB	0.151
RASSF8	0.151
BOK	0.151
KCNQ1	0.151
HDC	0.151
TNFRSF10C	0.151
PLA2G15	0.151
TMEM163	0.151
EDNRA	0.152
FGF7	0.152
AC008063.2	0.152
CASP1	0.152
NRGN	0.152
VGLL3	0.152
CLDN5	0.152
RP11-276H19.1	0.152
LAMB2	0.152

Gene Name	Fold change relative to normal
MAGI2-AS3	0.152
RHOBTB2	0.152
DOCK8	0.152
FMO3	0.153
SNX20	0.153
MYOF	0.153
TMC5	0.153
MGP	0.153
SPDEF	0.153
SLAMF8	0.153
MEFV	0.153
SPRED1	0.153
CAMK1G	0.153
RTN4RL1	0.153
RP11-276H7.2	0.154
GPR85	0.154
KLHL4	0.154
SNCG	0.154
STARD13	0.154
NFKBIA	0.154
LEPR	0.154
SEC14L4	0.154
TMEM88	0.154
CATIP-AS1	0.154
C11orf88	0.154
LMO7	0.154
SELP	0.154
GS1-115G20.1	0.155
RP11-863P13.4	0.155
FAM216B	0.155
CD40LG	0.155
LHFPL3-AS1	0.155
RP11-8L8.2	0.155
RP11-557H15.4	0.155
CEBPB	0.155
CCND1	0.155
HSD11B1	0.155
GIMAP6	0.155
RP11-160E2.17	0.155
GALNT18	0.156
TNFRSF1A	0.156
WDR86-AS1	0.156
RP11-472F19.1	0.156
RPS6KA2	0.156
RNF125	0.156
RP11-283G6.3	0.156
OSM	0.157

**Gene Name**	**Fold change relative to normal**
NLRP3	0.157
TNFRSF12A	0.157
TWIST2	0.157
RP11-483P21.2	0.157
RP11-356K23.1	0.157
MLKL	0.157
FCRL1	0.157
TMEM37	0.157
CDHR4	0.157
TMEM144	0.157
PPP1R15A	0.157
PLTP	0.158
ROPN1L	0.158
DOK3	0.158
PLEKHO2	0.158
WFS1	0.158
PDE4D	0.158
VDR	0.158
CLCF1	0.158
TIMM8AP1	0.158
RP11-647P12.1	0.158
RP4-541C22.5	0.158
HAVCR2	0.158
AL365331.2	0.158
RFTN1	0.158
CLEC10A	0.158
LCN2	0.158
AC096558.1	0.158
EGR3	0.158
MMRN1	0.158
MVP	0.158
GLIPR1	0.159
PGM5P4	0.159
AMY1A	0.159
GPR39	0.159
IL16	0.159
S100A11	0.159
RP11-27M24.2	0.159
LGI4	0.159
ERG	0.160
PID1	0.160
STAP1	0.160
WAS	0.160
CHI3L1	0.160
CSTA	0.160
PALM3	0.160
CCDC17	0.160

Gene Name	Fold change relative to normal
EMILIN2	0.160
IGFALS	0.161
AP000439.2	0.161
CD101	0.161
OR7E47P	0.161
SPNS2	0.161
SNHG18	0.161
PARVG	0.161
CSGALNACT1	0.161
C10orf10	0.161
PDK4	0.161
IRX5	0.161
GSG1L	0.161
CXorf21	0.161
PCED1B-AS1	0.162
SLC9A3R2	0.162
NINJ2	0.162
SLC38A5	0.162
CTD-2547H18.1	0.162
XAGE1B	0.162
LRCOL1	0.162
C2orf54	0.162
PLEK	0.162
AMY1B	0.162
ARHGAP30	0.162
BMPR1B	0.162
WBSCR17	0.162
RP6-99M1.3	0.162
Y_RNA	0.162
AC098617.1	0.163
DEFA1	0.163
SCN1A	0.163
SSMEM1	0.163
CMKLR1	0.163
SOX7	0.163
CRTAP	0.163
MIR3677	0.163
PRSS8	0.164
IRF8	0.164
RP11-26P13.2	0.164
FUCA1	0.164
KCNE1	0.164
RP11-114M1.1	0.164
PPP1R14C	0.164
C4BPAP1	0.164
RP11-716O23.2	0.164
C16orf54	0.164

Gene Name	Fold change relative to normal
C4BPB	0.164
TRGV7	0.164
GRN	0.165
FZD4	0.165
CTB-60B18.18	0.165
MBL1P	0.165
RPL7P19	0.165
SCUBE1	0.165
METTL7B	0.166
PIEZO1	0.166
HLA-E	0.166
RP11-23D24.2	0.166
CD300E	0.166
CXorf36	0.166
KCNAB1	0.166
LRRC4B	0.166
AC006159.5	0.167
F10	0.167
SYNE1	0.167
TLR3	0.167
RP11-153M7.5	0.167
ATXN1	0.167
GUCY1A2	0.167
RP11-1334A24.6	0.167
SPRY4	0.167
AC093673.5	0.167
AC124861.1	0.168
GAS2L2	0.168
RP5-1091N2.9	0.168
CCL21	0.168
KIF17	0.168
NDRG2	0.168
RSPO3	0.168
WDR63	0.168
RAC2	0.168
HMGB3P10	0.169
CXCL13	0.169
SPATA18	0.169
DUSP6	0.169
GPR34	0.169
C1orf132	0.169
DAPP1	0.169
RP11-692N5.2	0.169
GCOM1	0.169
MIR126	0.169
ARSJ	0.169
DNAH12	0.170

**Gene Name**	**Fold change relative to normal**
MILR1	0.170
P2RX7	0.170
FTO-IT1	0.170
EVPL	0.170
RP11-169D4.1	0.170
RP11-779O18.1	0.171
HORMAD2	0.171
BCL6B	0.171
CA13	0.171
FCGR2B	0.171
CTSZ	0.171
SSPN	0.171
CTSB	0.171
TACC1	0.171
AC096921.2	0.171
AKR1C2	0.171
PEAR1	0.172
LAMC3	0.172
RP11-389C8.2	0.172
CTA-363E6.6	0.172
CTSE	0.172
PLCD1	0.172
POU1F1	0.172
ABI3	0.172
IL10RA	0.172
PKNOX2	0.172
CFH	0.172
AGR3	0.172
AKAP14	0.172
MRGPRX2	0.172
BANK1	0.172
KCNK6	0.173
LILRA2	0.173
MT1L	0.173
COMT	0.173
OR7E36P	0.173
ABCA4	0.173
OMD	0.173
PTPLAD2	0.173
TM4SF1	0.173
SOX8	0.174
RP11-59D5__B.2	0.174
NCKAP1L	0.174
TXNIP	0.174
FTCDNL1	0.174
HSPB7	0.174
SMIM1	0.174

**Gene Name**	**Fold change relative to normal**
RARA-AS1	0.174
RP11-3K16.2	0.174
SGMS1	0.174
CECR1	0.174
PLA2G5	0.174
RP11-368J21.3	0.175
DES	0.175
EFCC1	0.175
NR2F2-AS1	0.175
CXCR5	0.175
EDEM1	0.175
ACSM2B	0.175
VEGFC	0.175
JDP2	0.175
RNU1-38P	0.175
ARPC1B	0.175
CLEC4A	0.175
RP1-151F17.1	0.175
ERBB2	0.175
AC020594.5	0.176
CDKL2	0.176
CD109	0.176
ITPRIP	0.176
CD79B	0.176
ARL11	0.177
SLC16A12	0.177
NR4A3	0.177
LCP1	0.177
PSORS1C1	0.177
PROCR	0.177
IL7	0.177
NMUR1	0.177
AC061992.2	0.177
RAB43	0.178
IL15RA	0.178
KCNA3	0.178
CCDC64B	0.178
SCD	0.178
KCNQ3	0.178
ECM2	0.178
NTM	0.179
RP11-370F5.4	0.179
CTD-3224I3.3	0.179
LINC00035	0.179
C1orf158	0.179
MFAP5	0.179
RP11-378A13.1	0.179

Gene Name	Fold change relative to normal
RPL9P33	0.179
DIO3	0.179
LHFPL3	0.179
SAMHD1	0.180
AC092375.1	0.180
CARD9	0.180
DPYD	0.180
MMP25	0.180
FOLR2	0.180
RP11-561I11.3	0.180
IFNGR1	0.180
LINC00402	0.180
VTN	0.180
CYTIP	0.180
GPC4	0.180
PRICKLE1	0.180
AKAP7	0.181
RAB20	0.181
TBC1D10C	0.181
CCND2	0.181
SELM	0.181
BTBD19	0.181
CTD-3105H18.14	0.182
RP11-136H19.1	0.182
TOR4A	0.182
MAGI1-AS1	0.182
RP11-332H18.5	0.182
EIF4EBP3	0.182
TNRC6C-AS1	0.182
RAB40A	0.183
MT1X	0.183
HTR1F	0.183
MPEG1	0.183
DAPK1	0.183
RBMS2	0.183
SYNPO2	0.183
TYMP	0.183
MS4A6A	0.183
DEFB124	0.183
RP13-258O15.1	0.183
GSAP	0.183
RP5-1042I8.7	0.183
ITGB4	0.184
TSPAN4	0.184
ARID5B	0.184
C22orf34	0.184
IL12RB1	0.184

Gene Name	Fold change relative to normal
MBNL1-AS1	0.184
LYL1	0.184
CPNE8	0.185
RP11-342A23.2	0.185
RARA	0.185
RP11-231P20.2	0.185
LINC00996	0.185
HMHA1	0.185
RP11-13N12.1	0.185
MARCH2	0.186
MAPKAPK3	0.186
CDK15	0.186
CCL22	0.186
PCDP1	0.186
LUM	0.186
PABPC5	0.186
CD244	0.186
RP11-568K15.1	0.186
IQGAP2	0.186
TTN	0.186
PRKCD	0.186
RNASE6	0.186
LL22NC03-86G7.	0.187
IER2	0.187
GIMAP7	0.187
AC007405.4	0.187
EGFL6	0.187
MATK	0.187
RP13-884E18.2	0.188
RBM47	0.188
CPEB2	0.188
GIMAP5	0.188
SLC6A7	0.189
GAS1	0.189
RMDN3	0.189
DPT	0.189
GYPB	0.189
TMEM64	0.189
NOD2	0.189
DOK4	0.189
C19orf38	0.189
SERPINB1	0.190
RP11-354P11.4	0.190
NKD2	0.190
TNFSF14	0.190
RP4-728D4.2	0.190
RP11-845C23.2	0.190

Gene Name	Fold change relative to normal
SERTAD1	0.190
PPM1M	0.190
ENPP2	0.190
ATP11A	0.190
RP11-46D6.1	0.190
RP5-875H18.9	0.190
C7orf72	0.190
ARHGAP24	0.191
ARHGAP15	0.191
TRPM4	0.191
IGSF6	0.191
RP11-64B16.4	0.191
THRB	0.191
RP11-714G18.1	0.191
LRRC4	0.192
RGS5	0.192
TNS4	0.192
ACKR1	0.192
GIMAP2	0.192
SASH1	0.192
CTB-133G6.1	0.192
MKX	0.192
KCNA3	0.192
HCST	0.192
PHYHD1	0.192
TESPA1	0.192
TACC2	0.193
PXMP4	0.193
BMPER	0.193
KCNA5	0.193
PSAP	0.193
ANGPTL5	0.193
KDR	0.193
LY96	0.193
RP11-366M4.11	0.193
GRIN3B	0.193
COL28A1	0.193
CTA-445C9.15	0.193
RP5-978I12.1	0.193
HEG1	0.193
SLC5A5	0.193
LINC01290	0.193
RP4-782L23.1	0.193
CD34	0.193
RXRG	0.193
RP11-138H10.2	0.194
MXRA8	0.194

**Gene Name**	**Fold change relative to normal**
BTC	0.194
KCNS3	0.194
NCAM2	0.194
DYNLRB2	0.194
FAM71A	0.194
PRKCQ-AS1	0.194
PIK3IP1	0.194
CATIP-AS2	0.195
CD14	0.195
NCEH1	0.195
USP44	0.195
PDCD1LG2	0.195
RP11-815J21.1	0.195
OTUD1	0.195
LINC01336	0.195
PCDHGA10	0.195
PTPN14	0.195
CDKL1	0.196
IKZF1	0.196
SH3D19	0.196
DDR2	0.196
NPL	0.196
RP11-38P22.2	0.196
FAM198B	0.196
GFPT2	0.197
TSPAN19	0.197
LYN	0.197
SNX25	0.197
MIR6124	0.197
CTC-205M6.5	0.197
SH2D4A	0.198
TMCC3	0.198
MROH6	0.198
SNX33	0.198
LMNA	0.198
ARHGAP29	0.198
PRCD	0.198
HSD17B13	0.199
TRIP6	0.199
TM4SF18	0.199
LPCAT2	0.199
CBX7	0.199
FAM209B	0.199
C10orf67	0.199
ETV1	0.199
CARNS1	0.199
TMC6	0.199

Gene Name	Fold change relative to normal
RAMP2	0.199
CCL24	0.199
PLAGL1	0.200
GALNT15	0.200
CTD-3128G10.7	0.200
PTPRC	0.200
IGF1	0.200
FTH1	0.200
EFNA1	0.200
LINC01093	0.200
CXCL12	0.201
IQSEC2	0.201
REST	0.201
SLC26A9	0.201
EDA2R	0.201
PZP	0.201
COX4I2	0.201
CREG1	0.202
RP11-345J18.2	0.202
CPQ	0.202
ZNF750	0.202
RP11-122K13.7	0.202
PLIN5	0.202
RIN1	0.202
ANK3	0.202
TEKT1	0.202
PRICKLE2	0.202
TSPAN18	0.202
PCDH12	0.203
PTPN22	0.203
RP11-357N13.2	0.203
PLD1	0.203
RP11-582J16.4	0.203
RPL26P30	0.203
MGAT1	0.203
LDLRAD1	0.203
RNF149	0.203
ST3GAL5	0.203
RP11-434H14.1	0.203
RP11-473M20.16	0.203
PTGIR	0.203
CGN	0.203
RP11-151D14.1	0.204
NCMAP	0.204
MT-TN	0.204
GOLGA2P7	0.204
BIRC3	0.205

Gene Name	Fold change relative to normal
ERICH2	0.205
PLLP	0.205
GAB1	0.205
MIR222HG	0.205
QSOX1	0.205
MT1E	0.205
C1orf87	0.205
LGALS9	0.205
MYD88	0.205
CUBN	0.205
Y_RNA	0.206
AC026202.3	0.206
SMAGP	0.206
GIMAP4	0.206
DKK3	0.206
PCDHAC2	0.206
SNED1	0.206
RP11-347C12.3	0.206
GRK5	0.206
VILL	0.207
RNU6-917P	0.207
CYTH4	0.207
ZFP36L2	0.207
AMN	0.207
PPP1R1B	0.207
RP11-472N13.3	0.207
PTPRM	0.208
AC135178.7	0.208
LINC01140	0.208
GPR157	0.208
ALDH1A2	0.208
MT-TC	0.208
PNPLA6	0.209
CYP1B1	0.209
FTH1P15	0.209
EVA1B	0.209
GM2A	0.209
MUM1L1	0.209
EVA1C	0.210
RNU6-1010P	0.210
NABP1	0.210
CYP2G1P	0.210
HLA-DMB	0.211
LPAR6	0.211
PRDM6	0.211
MED4-AS1	0.211
F2RL3	0.212

**Gene Name**	**Fold change relative to normal**
SIAE	0.212
FILIP1L	0.212
MYH1	0.212
RCSD1	0.212
BEST1	0.212
APOD	0.212
CTB-11I22.2	0.212
LMF1	0.212
RBMS2P1	0.213
ANXA2	0.213
RHOF	0.213
H1FNT	0.213
LRP10	0.213
SEMA6B	0.213
RP11-6J21.2	0.213
S100A6	0.213
RP11-640B6.1	0.214
PARD6B	0.214
CELSR1	0.214
LAMA2	0.214
VAMP5	0.214
SPINK14	0.214
ACSS2	0.215
CD79A	0.215
FUT7	0.215
SLC16A6	0.215
HFE	0.215
FCRLA	0.216
CTSL	0.216
PRKCE	0.216
RAB32	0.216
ADAMTS10	0.216
AC026806.2	0.216
TRIM6	0.216
IL6ST	0.216
RP11-218M22.1	0.217
LMOD1	0.217
HAS2	0.217
PPM1J	0.217
CLIC2	0.217
TNFRSF8	0.217
VAMP8	0.217
SMIM3	0.217
GAA	0.217
MIR23B	0.218
FAXDC2	0.218
ITM2A	0.218

**Gene Name**	**Fold change relative to normal**
ANXA2P2	0.218
VWA5A	0.218
GPR4	0.218
SLC27A1	0.218
PLK3	0.219
CSF1	0.219
ALPK1	0.219
TEX14	0.219
CU639417.1	0.219
CSTB	0.219
ARHGEF6	0.219
ADAP2	0.219
LCP2	0.219
GLDN	0.219
GZMM	0.220
PDGFA	0.220
MAN2B1	0.220
ITGA10	0.220
TNFAIP3	0.221
P2RY14	0.221
MECOM	0.221
KLHDC8B	0.221
PITPNM3	0.221
RP11-503N18.3	0.222
PCYOX1	0.222
AL928768.3	0.222
SLC7A2	0.222
PARP15	0.222
RP11-532F6.3	0.222
EHD1	0.222
GAS7	0.223
GANC	0.223
PAG1	0.224
IRF5	0.224
QKI	0.224
SDC1	0.224
PPL	0.224
RP3-453C12.14	0.224
PRSS22	0.224
TKT	0.225
PQLC3	0.225
COL14A1	0.225
FAM46C	0.226
ADAMTS9	0.226
CD180	0.226
RP11-94C24.13	0.226
LINC00935	0.227

**Gene Name**	**Fold change relative to normal**
SLC40A1	0.227
ADAMTS4	0.227
HLX	0.227
LGALS9DP	0.227
PSTPIP2	0.227
EPHX2	0.228
KLF6	0.228
CD83	0.228
TTC7A	0.228
PARVB	0.228
ST8SIA1	0.228
AP001205.1	0.228
CCDC135	0.228
LAMC2	0.229
SDC4	0.229
KIF13A	0.229
POLR3H	0.229
ACAA1	0.229
KCTD11	0.230
SPTBN1	0.230
TNS3	0.230
RP11-1094H24.4	0.230
MANBA	0.230
AC079767.4	0.231
NOTCH2	0.231
STK10	0.231
ANXA11	0.231
SEPT10P1	0.231
TLR1	0.232
GABARAPL1	0.232
FEZ2	0.232
GVINP1	0.232
NDST1	0.232
EPHA1-AS1	0.232
RASAL3	0.232
ANO2	0.233
FAS	0.233
INPP5D	0.233
FMNL1	0.233
LACTB	0.233
CD1D	0.233
PHACTR2	0.233
ANKRD20A11P	0.233
FAM92B	0.233
TRGC2	0.234
LGALS1	0.234
RNF145	0.235

**Gene Name**	**Fold change relative to normal**
PXDC1	0.235
FAM179A	0.235
ORAI3	0.235
ZNF257	0.235
FASN	0.236
NAGPA	0.236
TOM1	0.237
RP11-723D22.3	0.237
CYSLTR1	0.237
CEACAM4	0.237
SLC2A3	0.238
PPP2R5A	0.238
SELL	0.238
WWTR1	0.238
ST6GALNAC6	0.238
FHOD1	0.238
RP5-1009N12.1	0.238
HCLS1	0.238
C1S	0.239
BMP1	0.239
PPAP2B	0.239
SOX18	0.239
FAM78A	0.239
RP11-53M11.3	0.239
SUMF1	0.239
RP11-111E14.1	0.239
TSPAN12	0.239
FXYD5	0.239
SCARF2	0.239
RP11-394B2.6	0.239
TNFRSF1B	0.240
PIK3CG	0.240
IQSEC1	0.240
ITGA3	0.240
TNFSF9	0.240
PLAUR	0.240
EEF1A1P6	0.241
AASS	0.241
HRASLS2	0.241
KRT7	0.241
THRB-IT1	0.241
CHP1	0.241
RP11-62H7.2	0.241
RP11-262D11.2	0.241
FCRLB	0.241
RGS19	0.241
MARVELD1	0.241

Gene Name	Fold change relative to normal
CLC	0.242
AIM1	0.242
RP11-556I13.2	0.242
ZDHHC3	0.242
EEF1DP1	0.242
MYADM	0.243
MYLK	0.243
TPST2	0.243
CCDC102A	0.243
PIK3AP1	0.243
B4GALT1-AS1	0.243
LIMD1	0.243
RP11-249C24.10	0.243
STK17B	0.244
ICAM3	0.244
RP11-473M20.5	0.244
ATP1B2	0.244
C8orf58	0.244
RP11-24F11.2	0.245
NEIL1	0.245
ST6GALNAC2	0.245
ACTN2	0.246
MT2A	0.246
RP11-367J11.2	0.246
CTC-510F12.4	0.246
RP11-245J9.6	0.247
MAN2B2	0.247
ALDH1A3	0.248
ANTXRLP1	0.248
RP1-93H18.7	0.248
VAMP7	0.248
PLEC	0.248
RP11-58E21.3	0.249
RP11-420A23.1	0.249
MS4A1	0.249
CTDSPL	0.249
C4orf32	0.249
FCHO2	0.249
ZBTB4	0.249
C1orf194	0.249
GBP3	0.249
OCLN	0.250
NUDT18	0.250
FHL1	0.250
KCNE3	0.250
ABCA9	0.250
MR1	0.250

**Gene Name**	**Fold change relative to normal**
RHOQP2	0.250
SH3KBP1	0.250
TNKS1BP1	0.250
ROBO3	0.251
MT-ND6	0.251
GUCY1B3	0.251
USHBP1	0.251
LBH	0.252
PFKFB3	0.252
TPP1	0.252
FAM212A	0.252
STAT4	0.252
GPD1L	0.252
ARHGAP9	0.252
RYR2	0.252
ODF3B	0.253
HOXA-AS2	0.253
PPM1F	0.253
GPCPD1	0.253
PIK3R1	0.253
RPS20P35	0.253
EFCAB1	0.254
FYCO1	0.254
HPCAL1	0.255
RHOQ	0.255
OLFML3	0.255
CMTM7	0.256
GCSHP4	0.256
RP4-647J21.1	0.256
PTK2B	0.257
SEPTIN10	0.257
RP11-318K15.2	0.257
CST3	0.257
RNF180	0.257
CNN1	0.257
EPB41L2	0.257
RIN2	0.257
ARHGAP25	0.257
TST	0.257
MYO1C	0.257
HYAL2	0.258
MAFF	0.258
BCRP3	0.258
SAMSN1	0.258
AC011513.4	0.258
WI2-1896O14.1	0.258
REL	0.258

Gene Name	Fold change relative to normal
CTD-3252C9.4	0.258
AC093484.4	0.259
LINC00963	0.259
PMM1	0.259
B2M	0.260
RARRES1	0.260
STXBP6	0.260
UNC93B1	0.260
SH3TC1	0.260
APOL3	0.260
RP11-385D13.1	0.260
SNORD89	0.260
APLP2	0.261
ST3GAL6	0.261
MAFK	0.261
TMEM232	0.261
UPP1	0.262
KIAA0513	0.262
FLNA	0.262
TRIM22	0.263
DOCK11	0.263
RP11-452N17.1	0.263
KCNAB2	0.263
CTSA	0.263
ITIH3	0.263
RAB37	0.263
PPAP2A	0.263
PTGES	0.264
CLDN16	0.265
PCED1B	0.265
MYC	0.266
GLUL	0.266
TRADD	0.266
IL2RG	0.266
SPRYD3	0.266
C15orf26	0.266
CD40	0.266
KRT19	0.266
KIAA1217	0.267
USP25	0.267
IL15	0.267
SLC46A3	0.267
PDGFRB	0.267
IL32	0.267
BCL9L	0.267
ENTPD3	0.267
MEF2C	0.268

Gene Name	Fold change relative to normal
SOD2	0.268
RP11-286E11.1	0.268
FZD5	0.268
USP54	0.268
CD300LB	0.269
KIF16B	0.269
RP3-416H24.1	0.269
S100A16	0.269
SEPP1	0.269
PTP4A1P7	0.270
CD247	0.271
GALC	0.271
LAMA5	0.271
PHLDB2	0.271
PON2	0.271
BATF3	0.271
SLC16A3	0.272
ARID5A	0.272
SERPINB6	0.272
ARHGEF17	0.272
CCDC78	0.273
SPATA13	0.273
CD59	0.273
HIC1	0.273
MARCH2	0.273
PLA1A	0.274
CCPG1	0.274
LGMN	0.274
HSBP1L1	0.274
PLSCR4	0.274
CHPT1	0.275
EHBP1L1	0.276
ADAP1	0.276
VCL	0.276
POLD4	0.277
LAMA4	0.277
CACNB4	0.277
DTHD1	0.277
CD48	0.277
ATF7IP2	0.278
FBXO2	0.278
STAC3	0.278
PLK2	0.278
PDXK	0.279
SAT1	0.279
GPX1	0.279
RP4-607J23.2	0.279

Gene Name	Fold change relative to normal
ZBTB42	0.279
RP11-665G4.1	0.280
ARL6IP5	0.280
ZBTB47	0.280
CIB1	0.280
MCOLN1	0.280
ICAM2	0.281
ANXA4	0.281
NOS3	0.281
MXD4	0.281
MYL12A	0.281
SUCLG2	0.282
IQSEC3	0.282
TPT1P4	0.282
PLCE1	0.282
HSD17B11	0.282
FAM101B	0.282
PDGFB	0.283
AK1	0.283
RUNX1	0.283
RAB27A	0.283
SPRY1	0.284
CD58	0.284
CACHD1	0.284
IRAK2	0.285
ITPKB	0.285
IFITM3	0.286
GPR68	0.287
DOK7	0.287
FOSL2	0.288
TXNDC11	0.288
ITGB2-AS1	0.289
CAST	0.289
LINC00324	0.289
SFXN3	0.289
FAM13A	0.289
MOB3C	0.289
WDR11-AS1	0.290
RETSAT	0.290
AGR2	0.290
FTH1P23	0.290
NR1H3	0.291
HEXB	0.291
AGPAT2	0.291
AC010733.5	0.292
FTH1P10	0.292
TAGAP	0.292

Gene Name	Fold change relative to normal
AMT	0.293
AXIN2	0.293
TGFB2	0.293
NCOA4	0.293
ICOS	0.294
UTRN	0.294
LINC-PINT	0.294
C10orf11	0.294
LURAP1L	0.294
IL17RA	0.294
STAT6	0.294
TIMP2	0.294
SH2B3	0.294
ACTG2	0.294
EHD4	0.294
HSD17B4	0.295
PRKCZ	0.295
CD81	0.296
TCF7L2	0.296
TK2	0.297
WBP1L	0.297
FGD4	0.297
TRIOBP	0.297
IL17RE	0.297
NAGLU	0.297
FLI1	0.298
EEF1A1P13	0.298
ATG7	0.298
NR4A2	0.298
RNPEPL1	0.298
RAI2	0.298
BCL3	0.298
EDEM2	0.298
BTG2	0.299
RASL11A	0.299
JADE2	0.299
ABHD14B	0.299
MARCH1	0.300
UBA7	0.300
TMEM173	0.300
GLB1	0.301
ACCS	0.301
EPHX1	0.302
NAMPT	0.302
ARRDC4	0.302
EOGT	0.302
VAT1	0.302

Gene Name	Fold change relative to normal
RASSF2	0.303
ERAP1	0.303
RP11-469M7.1	0.303
TRIM8	0.304
TXK	0.304
SDC2	0.304
FAM21A	0.304
ZDHHC7	0.304
LGALSL	0.305
LRPAP1	0.305
SLC23A1	0.305
FTH1P2	0.305
NHSL1	0.305
NMRK1	0.305
SHANK3	0.305
GALM	0.306
RP5-1148A21.3	0.306
C20orf194	0.306
ERRFI1	0.306
SNX22	0.307
SLC22A4	0.307
RP11-507E23.1	0.307
RNASET2	0.308
AP5B1	0.308
TMEM63B	0.308
SPG20	0.308
BAIAP2	0.308
RP11-493K19.3	0.309
USP53	0.309
TNFSF13B	0.309
SQSTM1	0.309
ARHGAP23	0.310
CYB5A	0.310
MAP3K7CL	0.310
PPFIBP1	0.310
TPT1	0.310
SAT2	0.311
DNAH1	0.311
SNX24	0.311
SMAD7	0.312
HEXA	0.312
TMEM2	0.312
DNASE1L3	0.312
SESN2	0.312
TCIRG1	0.313
BAG3	0.313
PEAK1	0.313

Gene Name	Fold change relative to normal
RAB11FIP5	0.313
PHACTR1	0.313
PHYKPL	0.313
CTDSP1	0.313
EPS8L1	0.313
TGM1	0.314
CCR2	0.314
CCND3	0.314
COL4A3BP	0.314
IGFBP6	0.314
SLC6A6	0.314
DDX60L	0.314
TOM1L2	0.314
CRY2	0.315
EEF1A1P5	0.315
CFLAR	0.315
UBL3	0.315
YBX3	0.316
CYBA	0.316
SNX18	0.316
CDS1	0.316
JUN	0.317
ST3GAL1	0.318
CTBS	0.319
CCDC88B	0.319
CAPN2	0.319
TAOK3	0.319
METTL7A	0.319
AC016712.2	0.319
MAST4	0.320
RAB8B	0.320
SPATS2L	0.321
REEP5	0.321
CYB5R3	0.321
SNX30	0.322
ARRB2	0.322
MSMO1	0.323
GRINA	0.324
DHCR24	0.324
DOCK9	0.324
CD151	0.324
NAB2	0.325
FAM129B	0.325
CYSTM1	0.326
PAPLN	0.326
NFKB1	0.326
ADCY9	0.326

Gene Name	Fold change relative to normal
RP5-1159O4.2	0.327
PRR29	0.327
RARB	0.327
SMCO3	0.328
SEPT4	0.328
MTMR14	0.328
RNF130	0.328
NEDD4L	0.329
ITM2B	0.329
C10orf32	0.331
TAGLN	0.331
MT-ND5	0.331
MCL1	0.332
ARAP1	0.332
CYP4V2	0.332
MID1IP1	0.333
NEBL-AS1	0.333
ASL	0.334
SPSB2	0.334
LXN	0.335
NCOA7	0.335
ATP6V0E1	0.335
RP11-29G8.3	0.335
ENTPD3-AS1	0.336
SNX2	0.336
PTPN6	0.336
SSFA2	0.337
LRRFIP1	0.337
SIPA1	0.337
IL5RA	0.338
ALAS1	0.338
ARSA	0.338
MAP2K3	0.338
OSTF1	0.339
ERICH2	0.339
STAT5A	0.340
FBXW4	0.340
MPST	0.341
ELTD1	0.342
PDE4B	0.342
KIAA0930	0.343
PIK3CD	0.343
TNFSF10	0.343
ABLIM1	0.343
DOK1	0.344
FOXO1	0.344
PNRC1	0.345

**Gene Name**	**Fold change relative to normal**
TPT1P9	0.346
SNAP23	0.346
CYTH1	0.346
FAM65A	0.346
GNAI2	0.347
RHOG	0.347
TOB2	0.348
SCAMP2	0.349
UBE2Q2P6	0.349
DCUN1D3	0.349
ANXA5	0.349
SDCBP	0.350
PLEKHB2	0.350
IDUA	0.351
TMEM87A	0.351
RBKS	0.351
HDAC7	0.352
STK17A	0.352
STXBP2	0.352
RASSF5	0.352
MMP24-AS1	0.353
NFIL3	0.354
MKNK2	0.355
OBFC1	0.355
NFKB2	0.356
CASP7	0.356
LRP2BP	0.356
TAX1BP3	0.356
BLVRA	0.357
RGS10	0.358
MYO5C	0.359
RECK	0.359
EFCAB4A	0.359
BRI3	0.359
CD9	0.360
TRIP10	0.360
LITAF	0.361
GLTSCR2	0.361
SMARCD3	0.361
TMEM43	0.361
FNIP2	0.361
CC2D2A	0.362
TBC1D9	0.363
LMCD1	0.363
SFT2D1	0.363
XBP1	0.363
SHISA5	0.363

**Gene Name**	**Fold change relative to normal**
RAPGEF2	0.364
NEK5	0.365
NR3C1	0.365
EFR3A	0.366
CDHR3	0.366
TMEM120A	0.366
SUSD3	0.368
IGFBP7	0.368
PIM3	0.369
TBC1D22A	0.370
WIPF1	0.371
MT-ND3	0.371
IMPDH1	0.371
CAB39L	0.371
ANKRD44	0.373
SESN1	0.373
DESI1	0.373
SP100	0.374
OSTM1	0.374
DUSP5	0.375
ZBTB38	0.375
PRNP	0.377
CRIP1	0.377
GGA2	0.378
ERGIC1	0.378
RBMS1	0.379
CTD-3157E16.2	0.379
RPS27L	0.380
ETS1	0.380
ACSL4	0.381
CD63	0.381
ADD3	0.381
FGF2	0.382
SNX10	0.382
TSPAN13	0.383
TRIM16	0.384
SLC25A37	0.384
VASP	0.385
B4GALT1	0.386
SMIM14	0.386
AC114498.1	0.386
TES	0.387
PRR13	0.388
DNAJB12	0.389
IQGAP1	0.389
EBF1	0.389
FAIM3	0.390

Gene Name	Fold change relative to normal
SH3BGRL3	0.391
IFIT5	0.391
TNIP2	0.391
CASP4	0.391
GSTK1	0.392
AKNA	0.392
DUSP3	0.393
MBNL1	0.393
PAPSS1	0.393
TPPP3	0.395
PNKD	0.397
PDLIM1	0.397
NR1H2	0.397
JUND	0.398
MIR6723	0.399
PDLIM5	0.400
SACM1L	0.401
NRBF2	0.401
MED15	0.402
PACSIN2	0.404
SGPP1	0.405
C12orf49	0.407
SOCS1	0.407
RAP1A	0.409
SKI	0.409
CCDC85B	0.410
ISG20	0.412
LAP3	0.413
ARHGEF1	0.413
SBDS	0.413
ARRDC3	0.414
TMSB4XP1	0.416
ARPC2	0.418
SWAP70	0.418
EPB41L5	0.418
CTD-2287O16.1	0.420
HIPK3	0.420
AC022431.1	0.420
SLC20A2	0.421
YPEL5	0.421
ELK3	0.422
C4orf3	0.423
PDCD4	0.423
GSTO1	0.424
SNX1	0.425
ARL8B	0.425
ISCU	0.427

Gene Name	Fold change relative to normal
CLTB	0.427
TNIP1	0.428
EML3	0.428
PTEN	0.431
PUS10	0.433
GLTP	0.435
SYPL1	0.436
CYFIP1	0.438
TSC22D1	0.438
EIF3L	0.439
GLUD1	0.440
LRRC23	0.442
STX12	0.443
PRDX5	0.447
RPL13	0.447
TRAM1	0.447
TMEM159	0.449
RPL13P12	0.449
FBXL5	0.451
RPL3	0.453
ETF1	0.454
CRLS1	0.454
PTPN1	0.456
ATP6V1E1	0.457
DSTN	0.459
NUMB	0.460
EZR	0.461
ANAPC16	0.466
FBXO7	0.467
WSB1	0.472
RPL9	0.473
RRM2B	0.474
TMOD3	0.475
ACTB	0.476
RPL34	0.477
RPS12	0.483
EEF1A1	0.487
CFL2	0.491
RPS20	0.503
RPS14	0.507
RPL14	0.515
PTP4A1	0.518
RPS24	0.521
RPS27A	0.530
RPS25	0.548

Supplementary Table 5: List of Notch pathway genes enriched in GSEA

Gene Name	Gene ID	P-value	FC relative to normal	Expression in cancer	Expression in normal	FDR
ASCL1	ENSG00000139352	3.64E-10	46.951	52381.507	1114.694	5.97E-08
DLL3	ENSG00000090932	8.05E-08	32.311	3720.250	114.170	5.47E-06
DNER	ENSG00000187957	8.35E-05	29.658	4387.691	146.976	1.84E-03
ONECUT1	ENSG00000169856	2.88E-04	26.944	35.679	0.361	5.06E-03
RIPPLY2	ENSG00000203877	6.14E-05	21.680	386.241	16.862	1.43E-03
HOXD3	ENSG00000128652	4.09E-03	6.186	23.146	2.903	4.10E-02
HOXD3	ENSG00000278500	1.32E-02	4.291	4.952	0.387	9.84E-02
MDK	ENSG00000110492	1.06E-06	6.130	17448.795	2845.605	4.86E-05
NR0B2	ENSG00000131910	4.55E-03	4.792	1334.924	277.785	4.44E-02
MESP1	ENSG00000166823	1.13E-04	4.759	476.771	99.402	2.34E-03
NEURL1B	ENSG00000214357	1.05E-05	4.011	6762.319	1685.068	3.29E-04
DTX3	ENSG00000178498	1.03E-03	2.613	3870.078	1480.639	1.41E-02
JAG2	ENSG00000184916	3.34E-03	2.570	4545.401	1767.954	3.51E-02
CDKN1B	ENSG00000111276	2.96E-03	1.876	11237.719	5989.367	3.20E-02
RPS27A	ENSG00000143947	3.84E-03	0.530	28169.443	53194.604	3.91E-02
ANXA4	ENSG00000196975	1.06E-03	0.281	3441.288	12244.611	1.43E-02
KRT19	ENSG00000171345	4.66E-06	0.266	5675.278	21299.344	1.67E-04
MYC	ENSG00000136997	4.75E-03	0.266	1587.685	5982.610	4.59E-02
NOTCH2	ENSG00000134250	7.90E-04	0.231	2419.812	10489.328	1.14E-02
KCNA5	ENSG00000130037	3.89E-03	0.193	16.980	92.361	3.95E-02
TP63	ENSG00000073282	2.95E-04	0.139	116.928	847.205	5.16E-03
BMP2	ENSG00000125845	2.31E-06	0.128	625.451	4885.299	9.32E-05
TGFB1	ENSG00000105329	1.14E-08	0.114	960.771	8447.230	1.06E-06
DTX4	ENSG00000110042	2.12E-07	0.092	606.659	6579.331	1.25E-05
CEBPA	ENSG00000245848	2.48E-04	0.084	237.201	2850.436	4.48E-03
TBX2	ENSG00000121068	1.30E-10	0.080	319.376	4018.305	2.55E-08
TMEM100	ENSG00000166292	2.68E-04	0.060	475.436	7962.384	4.78E-03
TGFBR2	ENSG00000163513	4.05E-12	0.057	2044.803	36069.602	1.47E-09
HNF1B	ENSG00000275410	8.81E-07	0.054	10.757	217.205	4.16E-05
ANGPT4	ENSG00000101280	6.30E-11	0.032	12.074	411.815	1.42E-08
CNTN6	ENSG00000134115	7.03E-09	0.031	29.971	1013.935	7.11E-07

Supplementary Table 6: List of genes from 4 pathways enriched in GSEA

Gene Name	P-value	FC relative to normal	Expression in cancer	Expression in normal	FDR	Pathway Name
CER1	2.43E-05	63.59812582	245.4751249	2.875509251	6.71E-04	BMP
DAND5	1.62E-04	22.27023927	103.6516828	3.699171911	3.15E-03	BMP
SOX11	6.92E-04	19.1967872	4754.919073	246.7455745	1.02E-02	BMP
VWC2L	3.88E-04	17.66795201	48.12948802	1.780712105	6.43E-03	BMP
FOXD1	1.14E-06	14.88642125	165.6873326	10.19727367	5.13E-05	BMP
RNF165	1.46E-04	6.398912653	1216.898998	189.329055	2.88E-03	BMP
ACVR2A	8.40E-04	2.811726766	2195.170946	780.0755199	1.19E-02	BMP
CTDSPL2	4.75E-03	2.413768643	6533.891156	2706.339485	4.59E-02	BMP
UBE2O	3.60E-03	2.101327927	3802.820356	1809.198355	3.72E-02	BMP
SMAD4	5.28E-03	1.962241921	9544.92196	4863.803804	4.96E-02	BMP
TRIM33	5.20E-03	1.87338004	6752.454372	3603.956937	4.91E-02	BMP
SKI	4.17E-03	0.408880836	2914.365452	7129.110288	4.15E-02	BMP
SMAD7	1.85E-03	0.311593115	1079.763007	3467.507336	2.23E-02	BMP
SPG20	4.20E-04	0.307679151	1749.649315	5688.853568	6.85E-03	BMP
ITGA3	6.62E-06	0.239882717	4792.315481	19980.91259	2.25E-04	BMP
BMPER	1.98E-03	0.192536381	94.98074748	497.5070711	2.35E-02	BMP
SMAD6	7.90E-06	0.12755741	244.9537311	1927.180666	2.60E-04	BMP
ENG	7.56E-09	0.12153824	1301.794102	10718.21154	7.55E-07	BMP
C10orf54	1.41E-09	0.106184152	785.945086	7410.135034	1.82E-07	BMP
CYR61	5.81E-08	0.093959076	2452.86674	26115.33544	4.18E-06	BMP
FSTL3	1.50E-07	0.092506673	537.6995642	5822.358968	9.35E-06	BMP
BMP4	1.55E-06	0.092096173	74.86617106	822.7711615	6.65E-05	BMP
ACVRL1	2.26E-08	0.089740073	723.2839672	8069.909028	1.86E-06	BMP
GREM2	4.62E-07	0.081567049	13.80578629	180.5167574	2.42E-05	BMP
SOSTDC1	1.15E-03	0.074859534	75.09637506	1015.522161	1.52E-02	BMP
DKK1	1.03E-07	0.074398197	10.50903103	153.6950263	6.78E-06	BMP
CHRDL1	5.62E-09	0.072361521	255.5510287	3544.406805	5.93E-07	BMP
GATA6	3.86E-12	0.055390683	150.4598025	2733.391297	1.41E-09	BMP
CAV1	2.49E-14	0.028013781	1790.150817	63937.20274	2.40E-11	BMP
ATP2B3	4.24E-05	88.48315779	1886.82196	20.33538187	1.06E-03	Calcium signaling
ADCY2	4.53E-06	49.25120083	3977.901353	79.7879054	1.63E-04	Calcium signaling
TACR3	6.69E-05	48.19854992	344.0985651	6.159936673	1.54E-03	Calcium signaling
CACNA1E	1.52E-08	45.04869609	1463.196723	31.50253282	1.35E-06	Calcium signaling
PRKCG	1.54E-04	34.52551166	217.9491251	5.341661993	3.02E-03	Calcium signaling

Gene Name	P-value	FC relative to normal	Expression in cancer	Expression in normal	FDR	Pathway Name
GRIN2C	5.49E-06	31.4288865	2868.582127	90.30397054	1.92E-04	Calcium signaling
CAMK2B	1.35E-06	28.7765234	4862.31382	168.0028275	5.91E-05	Calcium signaling
CACNA1A	5.36E-07	25.31665836	18718.79936	738.4261555	2.75E-05	Calcium signaling
AVPR1B	1.42E-06	16.27624179	36.49483494	1.303654334	6.14E-05	Calcium signaling
CCKBR	2.17E-04	16.24010675	262.4584671	15.22270538	4.02E-03	Calcium signaling
ADCY1	1.33E-10	15.56470948	6555.77866	420.2593026	2.58E-08	Calcium signaling
CACNA1B	1.25E-04	14.31484136	2330.635069	161.8823547	2.54E-03	Calcium signaling
GRM5	2.05E-04	13.84816353	20.85409833	0.578122491	3.83E-03	Calcium signaling
SLC8A2	2.35E-04	13.2897702	215.4779276	15.28906477	4.29E-03	Calcium signaling
ATP2B2	3.10E-04	12.4825988	218.7251819	16.60251895	5.36E-03	Calcium signaling
CACNA1G	7.01E-04	10.20324002	341.3202919	32.55015575	1.03E-02	Calcium signaling
MYLK2	2.94E-03	7.513037678	113.1414351	14.19244812	3.19E-02	Calcium signaling
CHRM5	9.01E-04	6.113588908	62.29108902	9.352526148	1.26E-02	Calcium signaling
HTR6	4.05E-03	4.275786626	17.93552147	3.428546869	4.07E-02	Calcium signaling
PLCB1	5.75E-05	4.080241973	3451.020423	845.0332612	1.36E-03	Calcium signaling
PLCB4	2.36E-03	3.284126522	2993.590456	910.8377248	2.69E-02	Calcium signaling
PHKA1	3.28E-04	2.622452374	1684.97018	641.8983025	5.62E-03	Calcium signaling
VDAC3	3.28E-03	1.951512874	8988.52414	4605.438553	3.47E-02	Calcium signaling
ADCY9	3.75E-03	0.326094091	1515.653839	4649.970011	3.84E-02	Calcium signaling
ITPKB	8.45E-04	0.285264487	1255.889974	4405.05133	1.20E-02	Calcium signaling
PLCE1	1.55E-03	0.28199756	532.1449996	1889.601465	1.93E-02	Calcium signaling
NOS3	7.91E-04	0.281163422	116.2067425	415.863408	1.14E-02	Calcium signaling
PDGFRB	3.46E-03	0.267184114	1567.228511	5868.467636	3.61E-02	Calcium signaling
PTK2B	1.48E-05	0.256548502	1893.11674	7382.074651	4.42E-04	Calcium signaling
RYR2	3.71E-03	0.252452596	142.6686407	568.091557	3.81E-02	Calcium signaling
MYLK	1.47E-04	0.242869953	2504.970921	10317.15955	2.89E-03	Calcium signaling
CYSLTR1	5.13E-03	0.236807884	228.3488493	967.5017473	4.86E-02	Calcium signaling
ERBB2	1.70E-07	0.175307804	1478.896253	8440.702077	1.04E-05	Calcium signaling
PLCD1	3.10E-06	0.171893018	237.1138877	1384.24467	1.18E-04	Calcium signaling
P2RX7	2.86E-04	0.169651456	317.9699405	1879.148559	5.03E-03	Calcium signaling
EDNRA	4.40E-05	0.151501404	296.7559273	1964.367441	1.09E-03	Calcium signaling
PLCB2	6.67E-06	0.146826443	697.9084062	4759.098997	2.26E-04	Calcium signaling
PLCD3	2.68E-05	0.143413198	166.3047334	1165.592305	7.25E-04	Calcium signaling
ADCY4	5.82E-06	0.138862546	159.2100246	1152.731005	2.01E-04	Calcium signaling
P2RX2	5.43E-04	0.136138147	8.850588404	71.35729726	8.39E-03	Calcium signaling
ITPR1	1.64E-09	0.132984195	573.8332887	4321.568463	2.04E-07	Calcium signaling
ADCY7	7.86E-05	0.102552507	251.3862942	2460.044604	1.75E-03	Calcium signaling

Gene Name	P-value	FC relative to normal	Expression in cancer	Expression in normal	FDR	Pathway Name
CHP2	3.16E-03	0.098109029	1.63919136	25.90059612	3.37E-02	Calcium signaling
BST1	1.76E-06	0.094032416	125.4151298	1343.378191	7.39E-05	Calcium signaling
P2RX1	5.90E-06	0.086699975	55.0601569	645.5994596	2.04E-04	Calcium signaling
AGTR1	1.36E-03	0.07722643	44.4762131	587.868514	1.74E-02	Calcium signaling
ADCY8	1.87E-05	0.07432983	12.74810857	183.9608498	5.38E-04	Calcium signaling
PTAFR	4.45E-08	0.070121139	507.1372672	7245.563234	3.29E-06	Calcium signaling
PDE1B	7.78E-08	0.06907475	72.72806923	1066.36643	5.31E-06	Calcium signaling
ADRB1	3.35E-06	0.065750018	97.69029337	1499.992653	1.26E-04	Calcium signaling
EDNRB	2.21E-10	0.047159822	497.4197741	10567.73745	3.90E-08	Calcium signaling
ADRA1A	1.44E-04	0.039706627	4.530461314	138.2830814	2.85E-03	Calcium signaling
TNNC1	1.06E-11	0.023613888	108.7571655	4646.992055	3.31E-09	Calcium signaling
ADRB2	2.58E-15	0.021057942	63.66013541	3069.581947	3.82E-12	Calcium signaling
OR51E1	2.50E-06	711.6332197	3689.053809	4.18533102	9.91E-05	GPCR
CALCA	5.17E-08	229.0152202	6783.963002	28.6266903	3.77E-06	GPCR
GABRG3	1.83E-05	170.6492226	693.0152637	3.066911369	5.30E-04	GPCR
BRS3	4.52E-06	108.0217973	125.1348097	0.167679236	1.63E-04	GPCR
GABRG2	4.95E-04	69.28928869	68.28928869	0	7.81E-03	GPCR
GRIK3	3.31E-05	69.10508064	6196.398153	88.68078897	8.64E-04	GPCR
ADCY2	4.53E-06	49.25120083	3977.901353	79.7879054	1.63E-04	GPCR
TACR3	6.69E-05	48.19854992	344.0985651	6.159936673	1.54E-03	GPCR
GRP	2.43E-06	45.58272122	57450.96108	1259.389015	9.66E-05	GPCR
CHGA	6.13E-07	43.30900625	794.5530103	17.369228	3.07E-05	GPCR
SORCS3	1.88E-03	42.70274275	91.10761109	1.156948363	2.26E-02	GPCR
PTH2	6.29E-05	42.39289966	41.39289966	0	1.46E-03	GPCR
GNG8	2.94E-03	37.77048715	614.4630874	15.29481465	3.19E-02	GPCR
TMEM145	7.16E-07	37.75653705	2096.442185	54.55176265	3.51E-05	GPCR
OPRD1	2.62E-04	35.69142893	128.1711461	2.619108285	4.70E-03	GPCR
KCNK2	1.12E-03	35.26482113	451.1890469	11.82266668	1.50E-02	GPCR
GPR6	1.94E-09	34.54562614	139.935248	3.079684283	2.38E-07	GPCR
HTR1E	2.87E-03	32.15388368	111.0523842	2.484878695	3.13E-02	GPCR
GNG4	2.57E-09	31.95374889	22053.53578	689.2018242	3.03E-07	GPCR
GNG13	4.04E-04	30.27736041	55.0927795	0.852631099	6.64E-03	GPCR
GABBR2	6.08E-05	29.98677811	961.165294	31.08631785	1.43E-03	GPCR
SSTR2	1.74E-05	29.48916593	2061.029585	68.9249884	5.07E-04	GPCR
DRD2	8.97E-08	29.45011355	1670.706103	55.76399515	6.01E-06	GPCR
ATRNL1	5.54E-04	29.35589029	632.0192374	20.56361913	8.52E-03	GPCR
TAAR1	3.33E-03	26.30907184	213.8238158	7.165389382	3.51E-02	GPCR

Gene Name	P-value	FC relative to normal	Expression in cancer	Expression in normal	FDR	Pathway Name
RGS7	2.39E-07	26.20801139	736.1335603	27.12626831	1.38E-05	GPCR
CACNA1A	5.36E-07	25.31665836	18718.79936	738.4261555	2.75E-05	GPCR
GHRH	2.20E-03	25.30988857	1831.406573	71.39884	2.55E-02	GPCR
SUCNR1	2.24E-04	24.27737334	14957.10695	615.1336624	4.13E-03	GPCR
DGKB	2.74E-05	23.85254444	1227.913917	50.52129241	7.38E-04	GPCR
CALY	1.13E-03	23.77278384	246.2843432	9.401993506	1.50E-02	GPCR
GABRR1	3.41E-04	23.14226193	102.3846838	3.467354319	5.81E-03	GPCR
PRLHR	2.87E-03	23.13996384	46.62286215	1.058035288	3.13E-02	GPCR
PENK	1.06E-04	22.82017322	2793.760114	121.4688388	2.22E-03	GPCR
NPPA	7.14E-06	22.2380525	691.291468	30.13093955	2.39E-04	GPCR
GRM4	3.84E-06	21.14186168	1035.389741	48.02074174	1.42E-04	GPCR
ENTPD2	2.45E-05	20.93010894	1485.32739	70.01383915	6.74E-04	GPCR
GPR144	9.87E-06	19.95137976	87.37020096	3.429277675	3.13E-04	GPCR
GRM2	8.96E-07	19.49790241	620.2748492	30.8636762	4.22E-05	GPCR
BAI2	4.08E-07	18.68223462	3469.838436	184.782831	2.17E-05	GPCR
GPR139	1.99E-03	18.60076829	17.60076829	0	2.35E-02	GPCR
ADCYAP1	9.86E-05	18.48414372	3958.682879	213.22052	2.09E-03	GPCR
NXPH4	2.07E-06	18.28273731	2659.577056	144.5239995	8.47E-05	GPCR
PROKR1	3.97E-03	17.46281696	115.8276887	5.690082647	4.01E-02	GPCR
TSHR	1.80E-03	17.2198513	435.1092617	24.32595979	2.18E-02	GPCR
GNGT1	1.47E-03	16.78703429	157.8445103	8.462333105	1.85E-02	GPCR
MTNR1B	6.06E-04	16.59779283	25.19335017	0.578122491	9.17E-03	GPCR
GPR158	3.18E-05	16.42598739	624.0676906	37.05358397	8.36E-04	GPCR
AVPR1B	1.42E-06	16.27624179	36.49483494	1.303654334	6.14E-05	GPCR
CCKBR	2.17E-04	16.24010675	262.4584671	15.22270538	4.02E-03	GPCR
CENPI	1.70E-13	15.88355388	1467.793091	91.47257276	1.07E-10	GPCR
ADCY1	1.33E-10	15.56470948	6555.77866	420.2593026	2.58E-08	GPCR
OR51E2	2.40E-03	15.51186049	25.94273255	0.736911737	2.72E-02	GPCR
APLP1	6.45E-17	15.31645822	5537.082685	360.5772396	2.02E-13	GPCR
GRM8	2.19E-04	14.53420597	1069.856582	72.6783684	4.06E-03	GPCR
CHRM4	1.40E-06	14.31998943	1066.968353	73.57885069	6.08E-05	GPCR
GRM5	2.05E-04	13.84816353	20.85409833	0.578122491	3.83E-03	GPCR
CELSR3	4.97E-10	13.78528819	4882.936799	353.2861586	7.70E-08	GPCR
GNAO1	3.77E-09	13.33402434	4436.961868	331.829891	4.26E-07	GPCR
FZD9	3.41E-06	13.21699065	1650.341281	123.9407921	1.28E-04	GPCR
NMU	4.67E-05	13.19619084	1021.389136	76.4760799	1.14E-03	GPCR
CCL25	5.06E-03	12.89260751	55.60985476	3.390877074	4.81E-02	GPCR

Gene Name	P-value	FC relative to normal	Expression in cancer	Expression in normal	FDR	Pathway Name
SCG5	2.01E-05	12.34042787	29.35671516	1.459940244	5.72E-04	GPCR
ZACN	5.21E-03	12.18374925	152.2773419	11.5804741	4.91E-02	GPCR
GRK1	4.22E-05	11.98359683	70.87789107	4.998023137	1.06E-03	GPCR
OR2B6	3.80E-05	11.53627388	36.44640191	2.24597026	9.71E-04	GPCR
GPR142	1.38E-06	11.35681305	54.11686535	3.853198262	6.02E-05	GPCR
GPR62	2.35E-05	11.07301628	52.93312019	3.870680114	6.53E-04	GPCR
OR2W6P	1.90E-04	10.97108683	9.971086835	0	3.59E-03	GPCR
GABRA3	8.56E-04	10.79247616	73.24029926	5.87889398	1.21E-02	GPCR
GPR19	5.27E-05	10.46257386	613.860875	57.76764968	1.26E-03	GPCR
GPR149	1.05E-03	10.44950739	11.47315034	0.193659172	1.43E-02	GPCR
GAP43	3.12E-03	10.411848	230.2961198	21.21470384	3.34E-02	GPCR
CDK5R1	3.95E-06	10.31356696	3354.502097	324.3483601	1.45E-04	GPCR
LGR4	7.49E-09	9.117346579	7688.772347	842.4221821	7.50E-07	GPCR
BAI1	3.03E-04	8.647167094	974.5701991	111.8196308	5.27E-03	GPCR
HTR3E	4.70E-03	8.637291207	10.65683114	0.349593392	4.55E-02	GPCR
DGKI	1.29E-04	8.472715039	143.9244771	16.10484496	2.61E-03	GPCR
GPR173	2.93E-08	8.420663415	1507.897862	178.1899032	2.35E-06	GPCR
TAC3	7.68E-04	8.10810137	54.27513361	5.817272144	1.11E-02	GPCR
GNAZ	5.45E-07	8.094189242	1943.085012	239.1827971	2.78E-05	GPCR
HTR1D	5.09E-03	7.798264142	76.50903028	8.939267107	4.84E-02	GPCR
GAL	4.66E-03	7.581370772	75.81451413	9.132008636	4.52E-02	GPCR
GPR37L1	4.30E-03	7.430433697	71.08278941	8.701020472	4.25E-02	GPCR
PCSK1N	2.18E-03	6.828050969	4274.688985	625.1946498	2.53E-02	GPCR
OR1J1	4.11E-03	6.230121405	7.647543872	0.388021727	4.11E-02	GPCR
CHRM5	9.01E-04	6.113588908	62.29108902	9.352526148	1.26E-02	GPCR
OR13A1	1.20E-03	6.013866636	5.013866636	0	1.58E-02	GPCR
CRHR2	8.40E-04	5.903483664	97.1416411	15.62436058	1.19E-02	GPCR
GPR61	1.66E-03	5.742880426	52.80112437	8.368317007	2.04E-02	GPCR
FZD3	9.48E-07	5.567284651	2894.657354	519.1202265	4.40E-05	GPCR
GPRC5B	5.05E-04	5.463753799	4975.696895	909.8567255	7.93E-03	GPCR
CPE	7.16E-06	5.341826908	29060.35666	5439.340384	2.40E-04	GPCR
GPSM2	1.07E-07	5.298408349	4111.462845	775.1694786	7.05E-06	GPCR
TAS2R38	5.21E-03	5.215921828	8.256366248	0.77463669	4.91E-02	GPCR
GPR63	2.17E-03	4.830263767	259.0556294	52.83880507	2.52E-02	GPCR
CELSR2	1.66E-07	4.772849405	6233.699397	1305.284541	1.02E-05	GPCR
GLP1R	1.68E-03	4.728882258	69.63566645	13.93707447	2.06E-02	GPCR
ADCY5	3.43E-05	4.728219703	1585.701116	334.5810888	8.92E-04	GPCR

Gene Name	P-value	FC relative to normal	Expression in cancer	Expression in normal	FDR	Pathway Name
POMC	1.34E-03	4.315204783	391.0959036	89.86379983	1.73E-02	GPCR
HTR6	4.05E-03	4.275786626	17.93552147	3.428546869	4.07E-02	GPCR
PLCB1	5.75E-05	4.080241973	3451.020423	845.0332612	1.36E-03	GPCR
GPR180	4.78E-04	3.040659268	3646.769524	1198.664021	7.60E-03	GPCR
INSR	1.20E-04	2.989932709	10505.6718	3513.016141	2.46E-03	GPCR
GPR161	2.23E-03	2.815386213	2780.120165	986.8290063	2.57E-02	GPCR
XPR1	1.20E-03	2.651384853	8189.522283	3088.14878	1.57E-02	GPCR
LPHN1	6.08E-04	2.587376667	6114.213804	2362.480309	9.19E-03	GPCR
GSK3B	1.86E-03	2.101031884	9220.488597	4388.028394	2.24E-02	GPCR
SIGMAR1	4.45E-03	1.915994641	4696.996297	2450.988224	4.37E-02	GPCR
EZR	6.72E-04	0.461082981	17993.38626	39025.35104	9.95E-03	GPCR
RAPGEF2	2.63E-03	0.364485275	1827.828016	5016.563514	2.93E-02	GPCR
RGS10	4.76E-03	0.358164849	655.3916918	1831.652463	4.59E-02	GPCR
GNAI2	1.69E-04	0.346710603	10982.63363	31678.54352	3.25E-03	GPCR
ELTD1	5.11E-03	0.341657063	523.6567962	1534.624031	4.85E-02	GPCR
PTPN6	2.96E-04	0.336038603	3110.360638	9257.938139	5.18E-03	GPCR
ADCY9	3.75E-03	0.326094091	1515.653839	4649.970011	3.84E-02	GPCR
ARRB2	1.13E-03	0.322314411	3612.975507	11211.5781	1.51E-02	GPCR
CCR2	3.21E-03	0.313660147	105.84957	339.6539558	3.41E-02	GPCR
GPR68	2.96E-03	0.286536012	180.7415567	633.2712574	3.20E-02	GPCR
PLCE1	1.55E-03	0.28199756	532.1449996	1889.601465	1.93E-02	GPCR
CACNB4	2.66E-03	0.277385296	258.962507	936.1892111	2.96E-02	GPCR
FZD5	1.53E-03	0.268460755	1184.425217	4414.636903	1.92E-02	GPCR
PDGFRB	3.46E-03	0.267184114	1567.228511	5868.467636	3.61E-02	GPCR
PPAP2A	7.28E-04	0.263373686	423.9666268	1612.550057	1.06E-02	GPCR
FLNA	7.74E-04	0.261882208	20396.33912	77886.45678	1.12E-02	GPCR
APLP2	2.66E-05	0.260731784	22662.86823	86923.07147	7.21E-04	GPCR
ACTN2	4.86E-03	0.24561065	42.38229671	175.6303564	4.66E-02	GPCR
RGS19	1.71E-05	0.241135346	606.3391404	2517.664937	5.00E-04	GPCR
PIK3CG	3.03E-03	0.239627683	248.4117623	1039.830338	3.26E-02	GPCR
CYSLTR1	5.13E-03	0.236807884	228.3488493	967.5017473	4.86E-02	GPCR
P2RY14	1.25E-04	0.220932878	94.95557034	433.3200126	2.54E-03	GPCR
GPR4	2.88E-03	0.218431406	139.2174637	640.9290448	3.14E-02	GPCR
CELSR1	6.19E-04	0.213715622	1819.444915	8517.071329	9.31E-03	GPCR
F2RL3	2.66E-03	0.211622216	165.4805527	785.687502	2.96E-02	GPCR
LPAR6	5.73E-05	0.210692634	428.4854267	2037.445384	1.35E-03	GPCR
GPR157	8.70E-05	0.208015478	258.9437035	1248.636357	1.90E-03	GPCR

Gene Name	P-value	FC relative to normal	Expression in cancer	Expression in normal	FDR	Pathway Name
GRK5	2.81E-04	0.206459494	1264.77568	6129.866918	4.95E-03	GPCR
PTGIR	1.92E-04	0.203476496	82.32939603	408.5283609	3.62E-03	GPCR
CXCL12	1.64E-04	0.200652326	697.4309425	3479.801627	3.18E-03	GPCR
CCL24	3.59E-03	0.199185533	26.9236971	139.1893834	3.72E-02	GPCR
RAMP2	1.55E-04	0.199132822	644.9994394	3243.063103	3.03E-03	GPCR
PSAP	1.58E-05	0.192643148	49748.80753	258247.5179	4.67E-04	GPCR
ENPP2	4.44E-04	0.190091358	1312.369965	6908.151363	7.17E-03	GPCR
IQGAP2	1.54E-03	0.186147471	730.4669569	3928.502514	1.93E-02	GPCR
CCL22	7.90E-04	0.185699616	50.57326467	276.7241321	1.14E-02	GPCR
HTR1F	3.95E-04	0.182673979	6.659729319	40.9311462	6.52E-03	GPCR
NMUR1	9.69E-04	0.177074523	33.18724696	192.0670004	1.34E-02	GPCR
CXCR5	4.09E-03	0.174776802	4.999095653	33.32432444	4.10E-02	GPCR
MRGPRX2	3.35E-03	0.172336605	0.317388498	6.644275556	3.52E-02	GPCR
AKR1C2	3.35E-06	0.17142009	116.4894201	684.3888584	1.26E-04	GPCR
GPR34	9.20E-04	0.169062933	263.6150121	1564.186455	1.28E-02	GPCR
CXCL13	4.94E-03	0.168824895	930.2673929	5515.173388	4.72E-02	GPCR
RAC2	7.65E-06	0.168411644	1078.143354	6406.771641	2.54E-04	GPCR
CCL21	3.42E-04	0.16780788	595.0201273	3550.800589	5.81E-03	GPCR
FZD4	6.00E-05	0.164539055	678.1093557	4126.34445	1.41E-03	GPCR
CMKLR1	1.62E-03	0.162874345	642.7451923	3951.403884	2.00E-02	GPCR
PLEK	9.75E-06	0.162218108	961.7193348	5933.72175	3.09E-04	GPCR
SPNS2	1.08E-05	0.160942235	350.7421499	2184.517985	3.37E-04	GPCR
GPR39	1.71E-03	0.159013347	58.02680117	370.2065832	2.09E-02	GPCR
PDE4D	4.41E-07	0.157804785	615.1573996	3903.554606	2.32E-05	GPCR
GPR85	7.89E-04	0.153578719	69.78103737	459.8779001	1.13E-02	GPCR
EDNRA	4.40E-05	0.151501404	296.7559273	1964.367441	1.09E-03	GPCR
CCR1	1.92E-05	0.147302415	435.4729579	2962.107956	5.50E-04	GPCR
OR6K3	1.80E-03	0.143339323	0.335573012	8.317561876	2.18E-02	GPCR
ADCY4	5.82E-06	0.138862546	159.2100246	1152.731005	2.01E-04	GPCR
RGS18	1.08E-04	0.137298312	98.33678587	722.5106105	2.25E-03	GPCR
DEFB4A	7.92E-04	0.134731248	0.547327491	10.48454803	1.14E-02	GPCR
PTGDR	2.92E-05	0.133206197	16.11536108	127.4877241	7.80E-04	GPCR
MRGPRF	7.38E-05	0.131820839	37.36223265	290.0179685	1.66E-03	GPCR
GPR110	7.25E-04	0.131563217	182.1656015	1391.225012	1.06E-02	GPCR
CCRL2	9.03E-07	0.130719697	188.6452516	1449.777935	4.25E-05	GPCR
AKAP13	4.64E-06	0.125693817	4684.776692	37278.29359	1.66E-04	GPCR
GLP2R	1.64E-05	0.124628622	4.686255491	44.62559861	4.83E-04	GPCR

Gene Name	P-value	FC relative to normal	Expression in cancer	Expression in normal	FDR	Pathway Name
CALCRL	2.15E-08	0.12450597	840.4512505	6757.32049	1.80E-06	GPCR
PDE6G	8.27E-05	0.122805909	45.38404742	376.7020803	1.82E-03	GPCR
C3AR1	2.22E-04	0.121366019	474.0822873	3913.458848	4.10E-03	GPCR
GABRE	2.10E-06	0.118033733	80.77075243	691.7744313	8.56E-05	GPCR
GPR123	1.17E-03	0.117918677	1.915311202	23.7230657	1.54E-02	GPCR
CX3CL1	1.16E-06	0.116410788	901.8914946	7755.080938	5.21E-05	GPCR
CX3CR1	4.67E-05	0.116305481	67.51466213	588.0922893	1.14E-03	GPCR
GNGT2	3.99E-06	0.115696425	58.96788749	517.321008	1.46E-04	GPCR
P2RY2	4.16E-08	0.114792769	52.71556626	466.9351039	3.13E-06	GPCR
GPR97	2.81E-03	0.113721878	29.14239541	264.0536197	3.08E-02	GPCR
P2RY13	9.77E-05	0.110822794	127.3190005	1156.875521	2.07E-03	GPCR
TGM2	1.24E-06	0.110335439	4155.951192	37674.57585	5.54E-05	GPCR
LRP1	1.05E-05	0.108523414	4884.78407	45019.55269	3.30E-04	GPCR
APOE	2.11E-04	0.108428591	8993.325896	82950.60749	3.92E-03	GPCR
PTGER2	3.19E-04	0.107052915	98.8264416	931.496246	5.48E-03	GPCR
GNG7	5.21E-09	0.102819188	101.7380867	998.2112253	5.53E-07	GPCR
ADCY7	7.86E-05	0.102552507	251.3862942	2460.044604	1.75E-03	GPCR
RAMP3	5.74E-07	0.101263172	275.5727976	2730.227879	2.91E-05	GPCR
ARRB1	4.01E-08	0.101262636	784.1072287	7752.177871	3.03E-06	GPCR
GPR87	4.67E-03	0.100914384	9.765956694	105.6840647	4.53E-02	GPCR
CISH	3.28E-09	0.100398571	395.4461499	3947.723037	3.75E-07	GPCR
CCL2	3.03E-06	0.099173725	506.3546535	5114.817259	1.16E-04	GPCR
GNG11	1.29E-12	0.098547542	606.2022685	6160.516111	5.73E-10	GPCR
GPR133	5.74E-06	0.094314595	413.1050478	4389.67831	1.99E-04	GPCR
C3	3.93E-07	0.094097715	5792.498703	61567.96256	2.10E-05	GPCR
GPR65	4.21E-08	0.093990621	138.0711542	1478.627989	3.15E-06	GPCR
NPR3	9.00E-05	0.090797592	283.6899312	3134.434805	1.95E-03	GPCR
P2RY12	3.25E-05	0.088054186	23.80967131	280.7545931	8.51E-04	GPCR
S1PR1	9.16E-09	0.084489982	701.978193	8319.25497	8.86E-07	GPCR
PSAPL1	4.81E-04	0.08259737	1.537772567	29.72461727	7.64E-03	GPCR
S1PR4	7.12E-09	0.080365574	96.27153758	1209.36325	7.19E-07	GPCR
AGTR1	1.36E-03	0.07722643	44.4762131	587.868514	1.74E-02	GPCR
VIPR1	6.41E-06	0.076979703	377.2993572	4913.274084	2.19E-04	GPCR
C5AR1	1.56E-09	0.07447867	576.8912597	7758.151141	1.98E-07	GPCR
ADCY8	1.87E-05	0.07432983	12.74810857	183.9608498	5.38E-04	GPCR
PTH1R	6.72E-08	0.07214764	36.92080028	524.6000102	4.68E-06	GPCR
CD97	1.76E-10	0.071348975	1030.750738	14459.62449	3.25E-08	GPCR

Gene Name	P-value	FC relative to normal	Expression in cancer	Expression in normal	FDR	Pathway Name
FCN1	7.45E-06	0.070440908	95.50807235	1369.057185	2.48E-04	GPCR
PTAFR	4.45E-08	0.070121139	507.1372672	7245.563234	3.29E-06	GPCR
CXCL1	1.67E-07	0.069441608	246.1928501	3558.722423	1.03E-05	GPCR
EMR3	5.60E-08	0.066045654	11.33786491	185.808126	4.06E-06	GPCR
ADRB1	3.35E-06	0.065750018	97.69029337	1499.992653	1.26E-04	GPCR
EDN1	2.25E-08	0.064152357	332.1849327	5192.650645	1.86E-06	GPCR
CA2	3.25E-12	0.063333849	364.6746923	5772.763915	1.20E-09	GPCR
EREG	9.71E-08	0.058157334	13.44561684	247.3885671	6.43E-06	GPCR
GPR126	7.52E-11	0.057377071	198.6929291	3479.361163	1.64E-08	GPCR
GPR17	9.42E-07	0.054760409	3.807133874	86.78484186	4.38E-05	GPCR
CCL17	2.30E-06	0.054491839	10.82089854	215.9297035	9.27E-05	GPCR
HPGD	1.85E-06	0.052719659	429.3862921	8162.677425	7.72E-05	GPCR
AGTR2	7.00E-04	0.050786953	94.28686608	1875.20758	1.03E-02	GPCR
CXCR1	1.06E-04	0.050106683	21.73033688	452.6388296	2.22E-03	GPCR
RXFP1	2.92E-06	0.049163169	36.46078899	760.9685649	1.12E-04	GPCR
GPR116	2.17E-12	0.048644244	1645.319099	33843.0682	8.98E-10	GPCR
CXCR2	5.23E-06	0.047199277	30.67709832	670.1352429	1.84E-04	GPCR
EDNRB	2.21E-10	0.047159822	497.4197741	10567.73745	3.90E-08	GPCR
SCTR	1.46E-07	0.047037072	95.96995065	2060.564339	9.14E-06	GPCR
AKR1C3	6.39E-14	0.041505004	132.2111541	3208.52036	4.77E-11	GPCR
HCAR3	4.29E-05	0.041104458	6.893260068	191.0292952	1.07E-03	GPCR
NPR1	2.42E-11	0.040331036	183.1349477	4564.589345	6.48E-09	GPCR
ADRA1A	1.44E-04	0.039706627	4.530461314	138.2830814	2.85E-03	GPCR
GPRC5A	1.82E-08	0.03825198	2108.71817	55152.1754	1.58E-06	GPCR
AREG	8.00E-13	0.037203463	120.2324825	3257.634384	3.83E-10	GPCR
FPR1	1.63E-08	0.036030132	215.8592327	6017.82977	1.43E-06	GPCR
CCL20	8.00E-06	0.033219699	99.22113059	3015.918703	2.63E-04	GPCR
CCL23	9.69E-05	0.030908794	3.833424906	155.3770129	2.06E-03	GPCR
EMR1	5.86E-11	0.029194362	26.74805354	949.4593268	1.33E-08	GPCR
PPARG	7.55E-20	0.028812268	157.1469696	5487.876039	5.92E-16	GPCR
PF4	6.29E-07	0.028055575	3.575422655	162.084256	3.13E-05	GPCR
CAV1	2.49E-14	0.028013781	1790.150817	63937.20274	2.40E-11	GPCR
AGRP	2.16E-15	0.024486733	8.474594595	385.9276672	3.48E-12	GPCR
RXFP2	2.14E-06	0.0240853	0.434539449	58.56078805	8.73E-05	GPCR
CXCL3	1.64E-13	0.022183877	59.49505964	2725.983245	1.05E-10	GPCR
ADRB2	2.58E-15	0.021057942	63.66013541	3069.581947	3.82E-12	GPCR
HCAR2	3.32E-13	0.020911719	30.80985792	1520.149806	1.80E-10	GPCR

Gene Name	P-value	FC relative to normal	Expression in cancer	Expression in normal	FDR	Pathway Name
FPR2	2.07E-09	0.017991581	46.06683192	2615.047597	2.54E-07	GPCR
PPBP	1.09E-08	0.017752394	7.609364591	483.9692134	1.03E-06	GPCR
CXCL2	1.08E-15	0.016868272	306.4248645	18224.03623	1.93E-12	GPCR
CXCL5	3.89E-13	0.008187224	28.64560614	3619.959441	2.07E-10	GPCR
FGF5	3.98E-06	250.0843804	1502.933697	5.013705035	1.46E-04	MAPK
CACNG5	2.68E-07	114.323245	239.568702	1.104285108	1.50E-05	MAPK
CACNA1E	1.52E-08	45.04869609	1463.196723	31.50253282	1.35E-06	MAPK
PRKCG	1.54E-04	34.52551166	217.9491251	5.341661993	3.02E-03	MAPK
CACNA1A	5.36E-07	25.31665836	18718.79936	738.4261555	2.75E-05	MAPK
FGF12	2.27E-08	15.3934378	2161.531901	139.484012	1.87E-06	MAPK
CACNA1B	1.25E-04	14.31484136	2330.635069	161.8823547	2.54E-03	MAPK
MAPT	1.83E-05	13.14448227	80.31690602	5.186390942	5.29E-04	MAPK
CACNG2	1.72E-03	12.64625778	63.00438713	4.061132568	2.10E-02	MAPK
CACNA2D1	7.51E-06	11.03474552	10628.59772	962.2843543	2.49E-04	MAPK
CACNA1G	7.01E-04	10.20324002	341.3202919	32.55015575	1.03E-02	MAPK
MAPK8IP2	5.70E-10	8.793468974	2464.539791	279.3830659	8.70E-08	MAPK
STMN1	6.52E-14	8.643312775	92106.78894	10655.53776	4.81E-11	MAPK
NTF3	9.88E-04	8.065565436	477.0552766	58.27114229	1.36E-02	MAPK
MAPK8IP1	9.84E-10	7.412878483	4631.668366	623.9486453	1.36E-07	MAPK
FGF9	9.64E-04	5.914575834	705.0320854	118.3715501	1.33E-02	MAPK
FGF17	3.33E-03	5.84373832	128.8429145	21.21915278	3.51E-02	MAPK
RPS6KA6	4.69E-03	5.514252496	1809.662792	327.3605154	4.54E-02	MAPK
RAC3	6.99E-06	5.283828579	1819.478089	343.5376892	2.36E-04	MAPK
DUSP4	2.58E-04	4.65670764	4090.987628	877.7297688	4.63E-03	MAPK
CACNB3	1.93E-05	4.076029334	3515.297815	861.6772595	5.54E-04	MAPK
MAPK12	1.89E-03	3.447943949	1229.604331	355.9096102	2.26E-02	MAPK
MAP2K6	1.08E-03	3.290546242	750.7035341	227.4433887	1.45E-02	MAPK
PPP5C	2.60E-05	2.722659607	9428.847311	3462.46906	7.07E-04	MAPK
GADD45G	3.53E-03	2.56738265	3635.258831	1415.329129	3.66E-02	MAPK
CDC25B	6.99E-05	2.457148456	11801.22489	4802.220116	1.59E-03	MAPK
ELK1	2.53E-03	2.08927206	4478.93334	2143.255612	2.84E-02	MAPK
NRAS	2.89E-03	2.039024506	9092.775873	4458.865905	3.15E-02	MAPK
RAP1A	9.38E-04	0.408570877	4369.919817	10697.06993	1.30E-02	MAPK
JUND	3.31E-04	0.397638012	10257.24327	25796.94425	5.66E-03	MAPK
DUSP3	2.65E-03	0.392789884	3326.185629	8469.649995	2.94E-02	MAPK
FGF2	5.18E-03	0.381772691	253.6959132	666.14021	4.90E-02	MAPK
DUSP5	2.60E-03	0.374634559	549.6021628	1468.704674	2.90E-02	MAPK

Gene Name	P-value	FC relative to normal	Expression in cancer	Expression in normal	FDR	Pathway Name
RAPGEF2	2.63E-03	0.364485275	1827.828016	5016.563514	2.93E-02	MAPK
NFKB2	3.39E-04	0.355578403	1075.890319	3027.559409	5.77E-03	MAPK
MKNK2	8.46E-04	0.35464169	5979.074516	16861.29984	1.20E-02	MAPK
MAP2K3	4.36E-05	0.338402115	1668.146923	4931.436447	1.09E-03	MAPK
NFKB1	1.27E-04	0.326074618	1733.616549	5318.69204	2.58E-03	MAPK
ARRB2	1.13E-03	0.322314411	3612.975507	11211.5781	1.51E-02	MAPK
TAOK3	1.44E-03	0.318981979	1507.25747	4727.34695	1.82E-02	MAPK
JUN	5.13E-03	0.316521107	17513.33224	55332.85249	4.87E-02	MAPK
TGFB2	3.14E-03	0.292979251	405.5352152	1386.590463	3.36E-02	MAPK
PDGFB	2.65E-03	0.282601253	638.2594819	2261.054659	2.94E-02	MAPK
CACNB4	2.66E-03	0.277385296	258.962507	936.1892111	2.96E-02	MAPK
MEF2C	2.85E-04	0.267936904	708.5218874	2647.093175	5.02E-03	MAPK
PDGFRB	3.46E-03	0.267184114	1567.228511	5868.467636	3.61E-02	MAPK
MYC	4.75E-03	0.265506185	1587.685439	5982.609917	4.59E-02	MAPK
FLNA	7.74E-04	0.261882208	20396.33912	77886.45678	1.12E-02	MAPK
FAS	1.38E-04	0.232672564	573.1946693	2466.822847	2.75E-03	MAPK
MECOM	2.52E-04	0.221360985	1561.326634	7056.822909	4.55E-03	MAPK
PDGFA	8.80E-06	0.219837675	368.1708183	1678.288222	2.84E-04	MAPK
CD14	4.64E-04	0.19451088	1354.723693	6968.911872	7.43E-03	MAPK
MAPKAPK3	3.12E-06	0.185630936	1051.170181	5667.075599	1.19E-04	MAPK
PLA2G5	3.85E-04	0.17426102	36.36384319	213.4130872	6.40E-03	MAPK
DUSP6	1.82E-07	0.169032279	2219.752443	13137.03761	1.10E-05	MAPK
RAC2	7.65E-06	0.168411644	1078.143354	6406.771641	2.54E-04	MAPK
RPS6KA2	1.32E-07	0.156055416	2063.889672	13230.77194	8.43E-06	MAPK
TNFRSF1A	3.39E-08	0.155998109	1589.905288	10197.23443	2.63E-06	MAPK
FGF7	3.60E-04	0.151571224	208.5338344	1381.411707	6.07E-03	MAPK
IL1R2	1.34E-03	0.122429152	51.64373102	428.9934287	1.72E-02	MAPK
NR4A1	2.34E-08	0.119400186	1837.467844	15396.5291	1.92E-06	MAPK
IL1B	2.11E-06	0.114573885	96.55690242	850.4759016	8.62E-05	MAPK
TGFB1	1.14E-08	0.113842842	960.7705508	8447.230363	1.06E-06	MAPK
RRAS	7.09E-11	0.103232344	697.7812849	6768.014987	1.57E-08	MAPK
ARRB1	4.01E-08	0.101262636	784.1072287	7752.177871	3.03E-06	MAPK
CHP2	3.16E-03	0.098109029	1.63919136	25.90059612	3.37E-02	MAPK
FGFR3	1.91E-05	0.09778406	348.0096079	3568.187133	5.48E-04	MAPK
EGF	8.66E-05	0.096658351	22.70470755	244.2422094	1.89E-03	MAPK
IL1R1	1.86E-08	0.092138537	1432.833859	15560.71727	1.61E-06	MAPK
RASGRP2	9.90E-09	0.090296033	166.4773197	1853.758331	9.44E-07	MAPK

Gene Name	P-value	FC relative to normal	Expression in cancer	Expression in normal	FDR	Pathway Name
MAP3K8	7.39E-13	0.07245303	291.4702115	4035.687124	3.62E-10	MAPK
FGFR2	1.37E-05	0.070806438	532.1341088	7528.458097	4.14E-04	MAPK
PLA2G12B	1.39E-03	0.066917413	13.0708473	209.2718349	1.77E-02	MAPK
CACNA2D2	7.13E-07	0.065156218	1482.145782	22761.92004	3.50E-05	MAPK
TGFBR2	4.05E-12	0.05671664	2044.803339	36069.6018	1.47E-09	MAPK
DUSP1	2.11E-16	0.056434711	4875.34923	86405.91379	5.50E-13	MAPK
RASGRP4	2.10E-08	0.049980307	39.15465542	802.4095391	1.78E-06	MAPK
FOS	1.09E-13	0.04796214	5301.259636	110549.9394	7.49E-11	MAPK
PLA2G2A	2.20E-05	0.046046185	31.71178169	709.412416	6.18E-04	MAPK
GADD45B	1.27E-12	0.04524149	770.4117456	17049.98004	5.71E-10	MAPK
IL1A	2.81E-08	0.04208595	9.26061916	242.8015345	2.26E-06	MAPK
FGFR4	4.61E-10	0.04179513	105.8142219	2554.661902	7.26E-08	MAPK
FGF10	9.14E-10	0.041493664	2.948929182	94.16944938	1.30E-07	MAPK
PLA2G4E	2.86E-04	0.0403535	33.52375272	854.5330378	5.03E-03	MAPK
NTF4	1.23E-09	0.038190112	5.671362936	173.6882272	1.63E-07	MAPK
PLA2G1B	1.29E-10	0.021556552	18.50079271	903.6341308	2.53E-08	MAPK
RASGRF1	2.25E-12	0.018242058	83.48843375	4630.518818	9.11E-10	MAPK
